# Virtual Screening Guided Design, Synthesis and Bioactivity Study of Benzisoselenazolones (BISAs) on Inhibition of c-Met and Its Downstream Signalling Pathways

**DOI:** 10.3390/ijms20102489

**Published:** 2019-05-20

**Authors:** Siqi Zhang, Qiaoling Song, Xueting Wang, Zhiqiang Wei, Rilei Yu, Xin Wang, Tao Jiang

**Affiliations:** 1 Key Laboratory of Marine Drugs, Chinese Ministry of Education, School of Medicine and Pharmacy, Ocean University of China, 5 Yushan Road, Qingdao 266003, China; zhang-siqi@qq.com (S.Z.); sql_simm@163.com (Q.S.); 2 Center for Innovative Marine Drug Screening & Evaluation, Qingdao National Laboratory for Marine Science and Technology, Qingdao 266100, China; 3 Center for High Performance Computing & System simulation, Qingdao National Laboratory for Marine Science and Technology, Qingdao 266100, China; joanwang0814@outlook.com (X.W.); weizhiqiang@ouc.edu.cn (Z.W.); 4 Laboratory for Marine Drugs and Bioproducts, Qingdao National Laboratory for Marine Science and Technology, Qingdao 266003, China

**Keywords:** virtual screening, benzoisoselenone, c-Met inhibition, docking, molecular dynamics simulation

## Abstract

c-Met is a transmembrane receptor tyrosine kinase and an important therapeutic target for anticancer drugs. In this study, we designed a small library containing 300 BISAs molecules that consisted of carbohydrates, amino acids, isothiourea, tetramethylthiourea, guanidine and heterocyclic groups and screened c-Met targeting compounds using docking and MM/GBSA. Guided by virtual screening, we synthesised a series of novel compounds and their activity on inhibition of the autophosphorylation of c-Met and its downstream signalling pathway proteins were evaluated. We found a panel of benzisoselenazolones (BISAs) obtained by introducing isothiourea, tetramethylthiourea and heterocyclic groups into the C-ring of Ebselen, including **7a**, **7b**, **8a**, **8b** and **12c** (with IC_50_ values of less than 20 μM in MET gene amplified lung cancer cell line EBC-1), exhibited more potent antitumour activity than Ebselen by cell growth assay combined with in vitro biochemical assays. In addition, we also tested the antitumour activity of three cancer cell lines without MET gene amplification/activation, including DLD1, MDA-MB-231 and A549. The neuroblastoma SK-N-SH cells with HGF overexpression which activates MET signalling are sensitive to MET inhibitors. The results reveal that our compounds may be nonspecific multitarget kinase inhibitors, just like type-II small molecule inhibitors. Western blot analysis showed that these inhibitors inhibited autophosphorylation of c-MET, and its downstream signalling pathways, such as PI3K/AKT and MARK/ERK. Results suggest that bensoisoselenones can be used as a scaffold for the design of c-Met inhibiting drug leads, and this study opens up new possibilities for future antitumour drug design.

## 1. Introduction

The proto-oncogene Met-encoded c-Met is a highly binding receptor tyrosine kinase which is the only known receptor for hepatocyte growth factor (HGF) and belongs to the RON subfamily [1]. c-Met induces a series of biological effects by binding to HGF, or by other means, to activate tyrosine kinase and regulates cell growth, migration, proliferation and survival. HGF/c-Met signalling pathways have been implicated in a wide variety of solid tumours such as liver, breast, pancreas, lung, kidney, bladder, ovary, brain and prostate cancers [2,3,4,5]. HGF or MET are expressed at abnormally high levels in neoplastic tissue compared with normal surrounding tissue, especially at the invasive front [6,7]: c-Met is therefore an important therapeutic target for the development of anticancer drugs [8].

**Figure 1:**
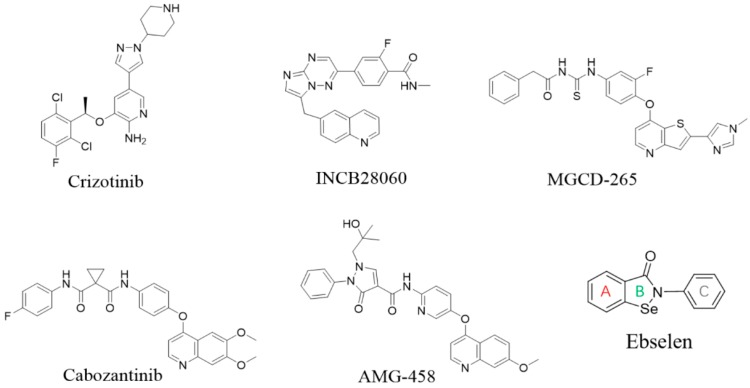
Structure of the known c-Met inhibitors and Ebselen.

The prevalence of HGF/Met pathway activation in human malignancies has driven rapid growth in drug development programmes and many molecules, such as c-Met kinase inhibitors, have been subject to clinical study in the last 10 years [9]. Most Met TKIs competitively antagonise occupancy of the intracellular ATP binding site, preventing phosphorylation, TK activation and downstream signalling. These inhibitors are classified as type-I or type-II inhibitors depending on their mechanism of action. Type-I inhibitors adopt a U-shaped conformation, usually interact with residue MET1121 and form π–π stacking with residue TYR1230. These inhibitors bind to the ATP binding site when the kinase has a ‘DFG-in’ conformation, in which the conserved DFG motif of the activation loop being in an ‘in’ conformation [10]. In contrast, type-II inhibitors bind to the kinase when it has a ‘DFG-out’ conformation, in which the conserved DFG motif of the activation loop being in an ‘out’ conformation. These inhibitors bind to c-Met in a more extended conformation than type I inhibitors, stretching from the ATP-binding site to a deep hydrophobic pocket. Most of the type-II inhibitors are nonspecific inhibitors, which have inhibitory effects on multiple kinase targets and are superior to type-I inhibitors [10]. Several drug candidates targeting c-Met have progressed into clinical trials, such as Crizotinib (type-I), INCB28060 (type-I), Cabozantinib (type-II), AMG-458 (type-II) and MGCD-265 (type-II) (Figure 1).

The research and development strategies adopted for new drugs mainly include the following aspects; separation and extraction from natural products, optimisation of existing drugs, drug design based on pathological mechanism, drug screening, etc. Virtual screening is attracting increasing levels of interest in the pharmaceutical industry as a productive and cost-effective method used in the search for novel lead compounds. Pioneering works are substantially useful to guide people to select appropriate methods for docking or virtual screening as well as deciphering protein recognition at the molecular level [10].

Benzisoselenazolones (BISAs) were not studied widely until they were found to have very good glutathione peroxidase (GSH-Px) antioxidant activity [11]. BISAs have been reported to have a broad spectrum of biological activities such as antioxidant, anti-inflammatory, antitumour activity, neuroprotective and anti-Alzheimer’s disease effects, and Ebselen is the best-known example [12,13,14]. Ebselen is a hydrophobic selenoorganic compound that potently inhibits lipid peroxidation through glutathione peroxidase-like action [15]. Moreover, Ebselen inhibits enzymes such as cyclooxygenases, lipoxygenases and indoleamine 2,3-dioxygenase, which play a broad functional role in cancer signalling as well as the regulation of the immune response [16,17,18]. The modest anticancer activity of Ebselen is in part due to its lower uptake by cancer cells and limited biodistribution [19]. In the past few years our group has synthesised a variety of sugar-containing Ebselen-derivatives inhibiting adhesion kinase (FAK), AKT-1 and protein kinase C-α (PKC-α), which are all associated with c-Met [20,21]. However, until recently, no BISAs directly interacting with c-Met have been reported, and fewer of selenium-containing c-Met drugs have been identified as preclinical candidates and entered clinical trials. Therefore, we want to verify if BISAs can inhibit c-Met, and elaborate their structure–activity relationship against the c-Met.

Preliminary studies have suggested that it is feasible to design antitumour molecules using BISA as a scaffold. Remarkably, isothiouronium-modified compounds have a wide variety of significant pharmacological activities, such as antimicrobial and antitumoural effects [22,23,24,25]. Compound MGCD-265 (Figure 1), with its acyl thiourea linkage (a key structural motif), showed potent inhibition of MET phosphorylation (with an IC_50_ of 8.7 nM) in epidermoid carcinoma cells (A431) stimulated by human recombinant HGF. Based on these pioneering studies, in this study, we intended to use the strategy of virtual screening to handle a molecular library generated using BISA as the scaffold, and carried out virtual screening against c-Met. In silico 300 BISAs compounds were designed and according to the predicted binding affinities of these benzisoselenazolone derivatives given by docking and MM/GBSA, four series of benzoisoselenazol-3(2H)-one derivatives were synthesised (Section 2.2) and their biological activity was evaluated. We hope that these compounds can provide guidance for the design of BISAs antineoplastic compounds inhibiting c-Met signalling pathways.

## 2. Results

### 2.1. Virtual Screening and Binding Energy Calculations

The poor water solubility of Ebselen is an important reason for its lower uptake by cancer cells and limited biodistribution. We hope to overcome this shortcoming by introducing some functional groups, such as isothiourea hydrobromide, improving the antitumourtumour activities and water solubility of compounds at the same time. To ensure structural diversity, a variety of carbohydrates, amino acids, isothiourea, tetramethylthiourea, guanidine and heterocyclic groups were introduced to benzisoselenazolone, and a library containing 300 compounds with distinctly different structures were established. In our previous studies, we found that conformation of the binding site significantly affected the accuracy of virtual screening [10]. In consideration of the influences of the conformation of the crystal structure to virtual screening, crystal structures bound with type-I inhibitor (PDB Code: 3ZZE) [26] and type-II inhibitor (PDB Code: 3U6I) [27], respectively, were selected for virtual screening and the predicted binding affinities are shown in Table S1. We have used some other c-Met PDB crystals for docking, such as 2KWM (type-I) and 5T3Q (type-II) and the PDB structures with the same type of inhibitors had similar scoring results. Therefore we chose these two representative cocrystals for this study. Furthermore, in previous docking studies these two crystals have been selected for docking and produced good correlation with experimental data [10]. Our group has previously studied efficient docking strategies and structure–activity relationship of the c-Met type-II inhibitors. MM/GBSA was used to re-score the binding affinities of the c-Met type II inhibitors in induced fit docking based on London dG score to further improve the correlation between the predicted binding affinities and the binding affinities derived from the experimental data. The results revealed that compounds with potential inhibitory effects on c-Met performed well in docking and MM/GBSA [10]. Virtual screening against 3U6I resulted in lower predicted binding energies than that given against 3ZZE. Thus, the predicted binding energies obtained from virtual screening against 3U6I were used for selection of candidate compounds. Compounds with predicted binding energies below −9 kcal/mol were selected and subjected to another round of binding affinity evaluation based on the MM/GBSA method. Binding free energy calculation was performed using the MM/GBSA script in AMBER 16 as previously described [28]. Before binding energy calculation, molecular dynamics simulations were performed for considering the flexibility of the protein and the ligand: because of its better performance in dealing with the flexibility of the protein, the MM/GBSA method gave a better ranking of the small ligand binding affinities compared to the docking scores [29]. The calculated binding affinities using MM/GBSA are shown in Table S2.

Preliminary virtual screening and calculation using MM/GBSA revealed that introducing glycosyl, isothiourea, tetramethylthiourea and heterocyclic compounds into the C ring of Ebselen could improve the binding affinity of the designed molecules to c-Met. Besides, we found that the predicted binding affinities of these ligands were related to their own conformation, and an extended conformation was usually more constructive to their binding with the c-Met.

The antitumour activity of the glycosyl modified BISAs derivatives that may bind well to c-Met have been studied and is shown to target multiple kinases capable of inhibiting cancer progression to metastases [15,16], therefore, we selected several new BISAs compounds containing isothiourea, tetramethylthiourea and heterocyclic groups for chemical synthesis and evaluated their biological activities. Meanwhile, compounds from the same series, but with poorly predicted binding affinities, were also selected for synthesis for a better understanding of the structure–activity relationship of these compounds.

### 2.2. Chemistry

**Scheme 1:**
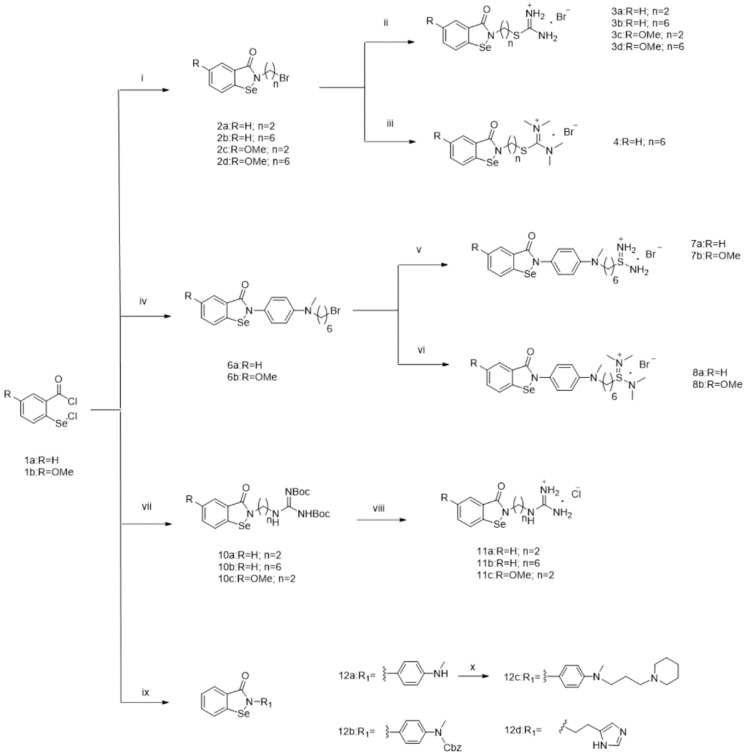
Reagents and conditions: (i) NH_2_(CH_2_)*_n_*Br, Et_3_N, THF, rt, 5 h, 50–70%; (ii) thiourea, THF, 70 °C, overnight, 40–60%; (iii) tetramethylthiourea, THF, 70 °C, overnight, 55%; (iv) *N*^1^-(6-bromohexyl)-*N*^1^-methylbenzene-1,4-diamine, Et_3_N, THF, rt, 5 h, 35–37%; (v) thiourea, THF, 70 °C, overnight, 45–53%; (vi) tetramethylthiourea, THF, 70 °C, overnight, 45–55%; (vii) **9a**~**b**, Et_3_N, THF, rt, 5 h, 50–70%; (viii) HCl, DCM, 3 h, 90–95%; (ix) R_1_NH_2_, Et_3_N, THF, rt, 5 h, 50–65%; (x) 1-(3-bromopropyl) piperidine, K_2_CO_3_, THF, rt, overnight, 40%.

We first designed and synthesised a series of benzoisoselenazolone analogues based on the performance of these benzoisoselenone derivatives in MOE [30] and MM/GBSA. The synthesis routes of the compounds are shown in Scheme 1.

For the benzoisoselenazolone compounds of alkyl isothiourea and 1,1,3,3-tetramethyl-isothiouronium hydrobromide (compounds **3a**~**d**–**4**), the starting compounds (**1a**~**b**) were obtained from the appropriate methyl anthranilate according to the procedure reported previously [31]. Subsequently, **1a** and **1b** were reacted individually with appropriate bromamine in the presence of Et_3_N to obtain compounds **2a**~**d** at a 50% to 70% yield. Then these compounds were reacted with thiourea or tetramethylthiourea in boiling THF overnight to obtain target compounds **3a**~**d**–**4** at a yield of 40% to 60%. For compounds with alkyl thiourea and tetramethylthiourea on the C ring of Ebselen (compounds **7a**~**b** and **8a**~**b**), the starting material *N*-methyl-4-nitroaniline derived from p-fluoronitrobenzene [32] was treated with sodium hydrogen and 1,6-dibromohexane to obtain **5** [33]. Afterwards, **6a**~**b** were obtained from treatment of the reduction products of compound **5**, which was deoxidised by Fe/HCl (Scheme S1), at a yield of 35% to 40% (a two-step process). Finally, the compounds were allowed to react with thiourea or tetramethylthiourea in boiling THF overnight to obtain target compounds **7a**~**b** and **8a**~**b** at a 45% to 60% yield. Compounds containing guanidine hydrochloride (compounds **11a**~**c**) can be synthesised by the following methods. **9a**~**9b** were prepared from the appropriate aliphatic diamine [34] (Scheme S2). Hereafter, these molecules were reacted with **1a**~**b** to produce **10a**~**c**. Compounds **11a**~**c** can be obtained through deprotection by HCl at high yield. Finally, for other groups or heterocyclic modified benzoisoselenones (compounds **12a**~**d**), *N*-methyl-4-nitroaniline and its derivative, protected by CbzCl in Scheme S1, were reduced by Fe/HCl and then reacted with **1a** to obtain **12a** or **12b**, respectively. 1-(3-Bromopropyl) piperidine was treated with **12a** to produce compound **12c** at a yield of 40%. Compound **12d** was made by reaction between **1a** and histamine with a high resultant yield.

### 2.3. Biology Assays

To evaluate the biological activity of these synthesised compounds, we first examined the growth inhibition activity of the compounds against the c-Met amplified cancer cell line EBC-1. Subsequently, we selected compounds with high antitumour effects to test their inhibitory effects on other cancer cell lines including A549, MDA-MB-231, SK-N-SH and DLD1. Finally, the inhibition of compounds for phosphorylation of c-Met and its downstream signalling pathways were studied by Western blot assay.

#### 2.3.1. EBC-1 Cell Viability Inhibition

**Figure 2:**
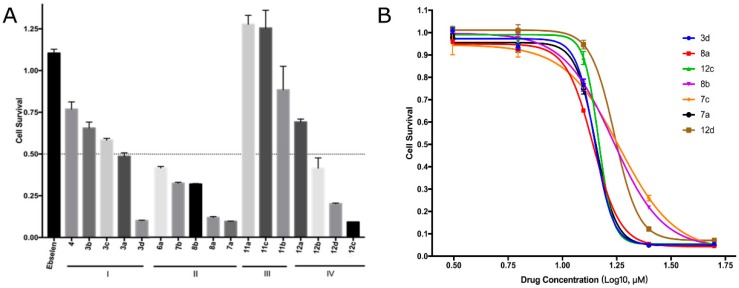
Effects of the tested compounds on cell viability of EBC-1 cancer cell line. (**A**) Percentage of viable cells (EBC-1) after 72 h exposure to the compounds at a concentration of 25 μM compared to the compound-free control received an equal volume of DMSO (100% viability). Each value was calculated from two independent experiments. The competitive inhibitory activity was expressed as an inhibition rate. (**B**) Growth inhibition effect of compounds **3d**, **7a**, **7b**, **8a**, **8b**, **12c** and **12d** on EBC-1 cells. EBC-1 cells were seeded into 96-well cell culture plates and allowed to grow overnight. Thereafter, cells were treated with vehicle control (DMSO) or the compounds at the indicated concentration for 72 h. After treatment, 10 μL resazurin were added to the culture medium. After incubation at 37 °C for 5 h, fluorescence intensity was measured at a 530 nm excitation wavelength and a 590 nm emission wavelength using a Synergy HT photometer.

We carried out a preliminary cell viability test: EBC-1 cells were treated with 25 μM concentrations of each compound for 72 h. Cell viability was measured as a basis for testing the antitumour effects of compounds (Figure 2A). The compounds from Scheme 1 (**3d**, **6a**, **7a**, **7b**, **8a**, **8b**, **12b**, **12c** and **12d**) could potently inhibit the growth of EBC-1 cells (by more than 50%). Subsequently, we treated EBC-1 cells with different concentrations of compounds to obtain their IC_50_ (Table 1). Most of the compounds containing isothiourea and tetramethylthiourea (**3a**~**d**, **4**, **7a**~**7b** and **8a**~**b**) showed different degrees of antitumour cytotoxic activity; in particular, the IC_50_ values of **3d**, **7a**, **7b**, **8a** and **8b** are lower than 20 μM, and all of them were generally long chain-length compounds. The results revealed that the antitumour activity is independent of the methylation of thiourea, but **7b** and **8b** showed decreased potency due to the modification of methoxy groups. Compounds **11a**~**c**, containing guanidinium, did not inhibit EBC-1 cells at 25 μM and had higher IC_50_ (IC_50_ > 50 μM) values compared to other compounds. The antitumour effects were also enhanced by introducing other group-modified compounds at C-ring of Ebselen, and **12c** had stronger cytotoxicity (**12c** > **12b** > **12a**). This is also consistent with the result of virtual screening. **12d** inhibited EBC-1 cell growth with an IC_50_ value of 17.28 μM indicating that the antitumour effects of Ebselen can be improved by introducing a heterocyclic structure. Indeed, as shown in Figure 2B, compounds **3d**, **7a**, **7b**, **8a**, **8b**, **12c** and **12d** inhibited the cell viability of the cancer cell in a concentration-dependent manner. These data demonstrated that introducing isothiourea, tetramethylthiourea or heterocyclic groups to Ebselen could inhibit the growth of the c-Met amplified cancer cells EBC-1 and in general, the length of the molecule increased the activity of the compounds.

#### 2.3.2. Survival Inhibition of Four Other Cancer Cell Lines

**Figure 3:**
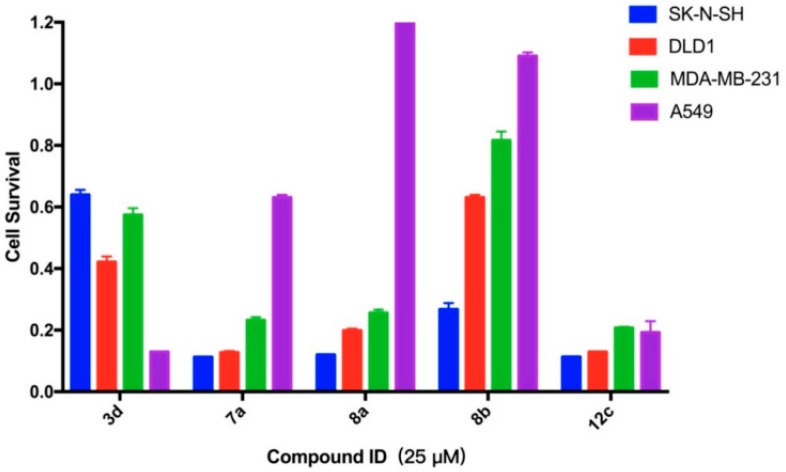
Effects of compounds **3d**, **7a**, **8a**, **8b** and **12c** on cell viability of four cancer cell lines. Percentage of viable cells (SK-N-SH, DLD1, MDA-MB-231 and A549) after 72 h exposure to the compounds at a concentration of 25 μM. Each value was calculated from two independent experiments. The competitive inhibitory activity was expressed as an inhibition rate.

**Figure 4:**
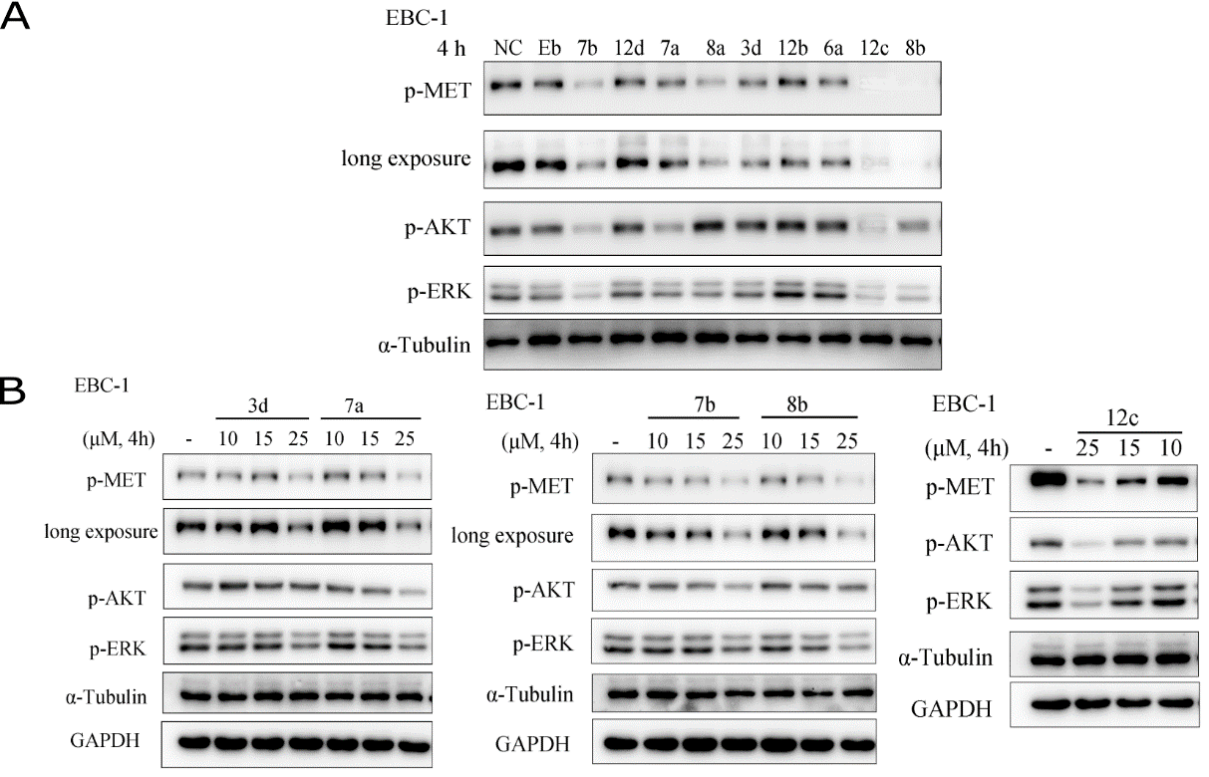
Effects of Ebselen and compounds on p-c-Met, p-AKT and p-ERK in EBC-1 cancer cells. (**A**) Western blot analysis in the EBC-1 cells treated with concentrations near the IC_50_ values of compounds for 4 h. Ebselen was unable to inhibit the phosphorylation of c-Met at 25 μM. (**B**) Representative Western blot analysis in the EBC-1 cells treated with increasing concentrations of compounds **3d**, **7a**, **7b**, **8b** and **12c**, as indicated. All of the tested compounds could inhibit the phosphorylation of Met, AKT and ERK in a dose-dependent manner.

To determine whether the antitumour effects of these compounds result from targeting MET signalling specifically, we tested the growth inhibition efficacies of these compounds in four human cancer cell lines neuroblastoma SK-N-SH, colorectal cancer DLD1, triple-negative breast cancer MDA-MB-231 and lung cancer A549 without MET gene amplification or overexpression (Figure 3) [35]. The previously published data also indicated that there was no obvious MET activation/phosphorylation in cancer cell lines A549, MDA-MB-231 and DLD1, which shares the same genetic origin with HCT-15 [36,37]. However, the data from the Cancer Cell Line Encyclopedia (CCLE) database suggested that there were more hepatocyte growth factor (HGF) expression—the ligands of MET kinase—in SK-N-SH cells compared with the three other cell lines [35]. The HGF overexpression in SK-N-SH cells would activate MET signalling and be responsible for the cancer cell growth, which is accordant with the clinical status of neuroblastoma cancer patients [38]. As shown in Figure 4, we measured the percentage of viable cells after 72 h exposure to those compounds with high inhibitory activity against MET gene amplified lung cancer cells EBC-1 (**3d**, **7a**, **8a**, **8b** and **12c**) at 25 μM, and the testing was repeated twice to determine the IC_50_ values in these four cancer cell lines. The compound **12c** caused low micromolar concentration inhibition of the cell viability of all cancer cells and exhibited the best antineoplastic activity at 25 μM no matter MET is activated or not. Therefore, the antitumour effects of compound **12c** could not only result from MET activation but also other targeted proteins/kinases in these cancer cell lines. Compound **3d** did not perform well in all three cell lines except for its antitumour activity against A549, which means compound **3d** might target the protein specifically expressed in A549 cells instead of MET. Interestingly, compound **7a** had the opposite results to **3d** and, similarly, compounds **8a** and **8d** also showed this phenomenon. The selectivity of compounds **7a**, **8a** and **8d** was in accordance with the MET activation status in these four cells, which suggested that these compounds exhibited higher affinity to MET. Nevertheless, compounds **7a** and **8a** could also efficiently inhibit the growth of DLD1 and MDA-MB-231 cells without MET activation. The compounds **7a** and **8a** might target multiple proteins/kinases except MET. From this we can infer that the existence of C-ring in the structure of these compounds may change the mechanism of their inhibitory effect on cancer cells. The IC_50_ values of the five compounds in four cancer cell lines and EBC-1 are shown in Table 2. The IC_50_ values of compounds **7a**, **8a** and **8b** in SK-N-SH and EBC-1 cells were comparable or even lower than those in DLD1, MDA-MB-231 and A549 cells, indicating that these three compounds might bind to MET more efficiently than other proteins/kinases. The compound **3d** and **12c** might exhibit antitumour effects in these five cell lines by targeting more than one protein/kinase with no less binding affinity than MET. By summarising the structure–activity relationships of **7a**, **8a** and **8b**, it can be seen that the introduction of methoxy on benzoisoselenone will lead to a decrease in the antitumour activity of the compounds and, except for A549, the effects caused by methylation of isothiourea on the activity of compounds is not significant.

From the aforementioned cytotoxicity experiments, we can find that the designed compounds have antitumour activity, but the mechanism of the antitumour effect may be different in various cancer cells. Although they have the best inhibitory effect against c-Met overexpressed EBC-1 cells, like type II c-Met inhibitors, these compounds may still have other kinase targets.

#### 2.3.3. The inhibitory effects on the activation of c-Met and its downstream signalling pathways

Most of the type-II inhibitors are nonspecific inhibitors that inhibit multiple kinase targets. We selected those compounds with strong antitumour effects to examine their inhibitory effects on the autophosphorylation of c-Met and its downstream signalling pathways (AKT and ERK) in the EBC-1 cells. Phosphorylation of c-Met protein activates downstream signalling pathways to exert corresponding biological effects. The AKT and ERK play an important role in the proliferation and invasion of cancer cells [39,40,41]. The status of p-Met, p-AKT and p-ERK were determined by Western blot assay at a drug concentration near their IC_50_ values. As shown in Figure 4A, compounds **3d**, **7a**, **7b**, **8a**, **8b** and **12c** significantly blocked c-Met phosphorylation, compared to Ebselen at 25 μM, while no inhibitory effects were observed with the other compounds. Correspondingly, the phosphorylation of AKT and ERK were inhibited to some extent. However, compound **12d**, with its strong antitumour effects, did not inhibit the activation of c-Met and downstream signalling pathways AKT and ERK. Other mechanisms might be responsible for its antitumour effects. Subsequently, we examined the inhibitory effects of compounds **3d**, **7a**, **7b**, **8b** and **12c** on the activation of p-Met, p-AKT and p-ERK in a dose-dependent manner (Figure 4B). All of the tested compounds could inhibit the phosphorylation of Met, AKT and ERK in a dose-dependent manner. Our data proved that nine of the BISAs derived from Ebselen could potently inhibit EBC-1 cancer cell growth by downregulating the activity of c-Met and its downstream signalling pathways AKT and ERK.

#### 2.3.4. Molecule Docking Studies and Molecular Dynamics Simulation

**Figure 5:**
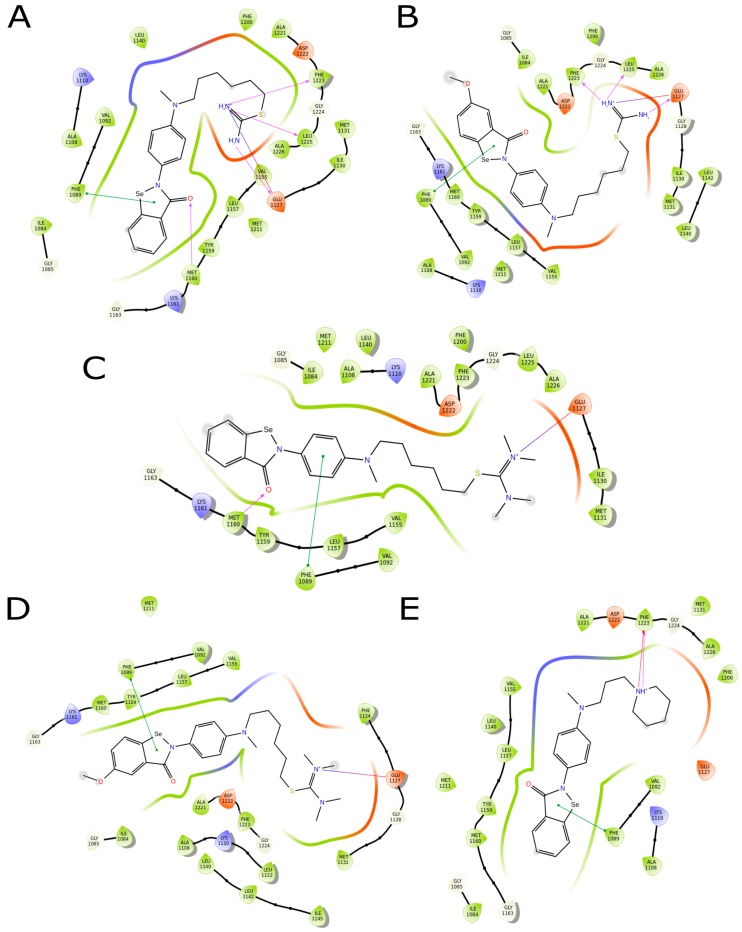
The binding mode of compounds in the ATP binding pocket of c-Met protein in two-dimensional panel. The green full line indicates the π–π stacking interaction, the purple arrow indicates the hydrogen bond and the fuchsia line indicates the ionic bond. The grey shade highlights the solvent exposure region of the small compounds. (**A**) The binding mode of compound **7a**. (**B**) The binding mode of compound **7b**. (**C**) The binding mode of compound **8a**. (**D**) The binding mode of compound **8b**. (**E**) The binding mode of compound **12c**.

We chose the bonded protein (PDB code: 3U6I) to understand the mechanism governing the behaviour of these molecules in the c-Met binding pocket by computational docking in Schrödinger and LeDock [42]. The binding mode of molecules **7a**, **7b**, **8a**, **8b** and **12c** in 3U6I is shown in Figure 5. Aromatic rings increased the affinity between molecule and hydrophobic pocket, and effectively enhanced the hydrophobic effects with ILE1084, PHE1089, ALA1108, MET1160, TYR1159 and LEU1157. Meanwhile, there were π–π stacking effects observed between all compounds and PHE1089 in the binding pockets. In addition, isothiourea, tetramethylthiourea and piperidine in these compounds could form hydrogen bonds or ionic bonds with different amino acid residues in the binding pockets (Figure 5). The interactions of known c-Met inhibitors cocrystallysed with the c-Met structure (3U6I) are shown in Figure S1.

In order to verify whether this docking methodology allows us to make such a prediction, we conducted cross-docking experiments. Four c-Met crystals with different ligands were selected for cross-docking and the PDB codes were 3ZZE (type-I), 3U6I (type-II), 5T3Q (type-II) and 3LQ8 (type-II), respectively. Firstly, the ligand separated from 3ZZE was selected to be docked with 3U6I, and the conformer obtained after docking is compared with the original conformer of the ligand in 3ZZE to show the RMSD of 2.943 Å, which was calculated by Pymol. It indicated that there was a certain deviation between the results obtained by the docking methodology of type-I and type-II inhibitors. Subsequently, we chose two kinds of ligands isolated from PDB structures with type-II inhibitors (5T3Q and 3LQ8) to dock using 3U6I as a receptor. RMSD were calculated to be 0.329 Å and 1.533 Å, respectively. Values were less than 2.0 Å, even close to zero when the structures of compounds are similar. It can be seen that this docking method is more accurate for the same type of c-Met inhibitors and the results of virtual screening indicate that the compounds we designed are more in line with type-II inhibitors, so docking in 3U6I is feasible.

**Figure 6:**
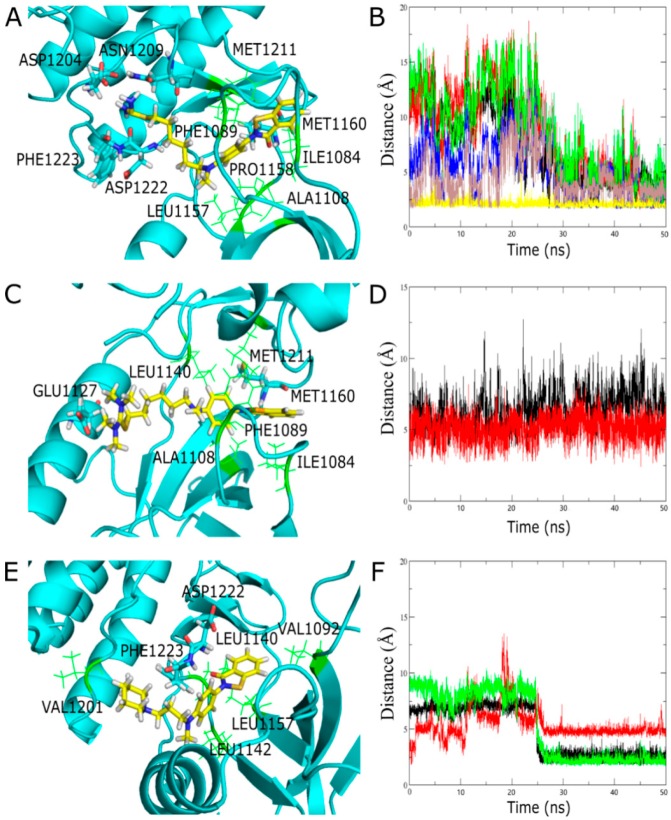
Analysis of the stability of compound/c-Met over 50 ns (from molecular dynamics simulations). (**A**) The binding mode of compound **7a** (yellow) in the binding site of c-Met (cyan and green) and its binding pocket. (**B**) The hydrogen bond interactions of **7a**–MET1160 (yellow), **7a**–ASP1204 (red and green), **7a**-ASN1209 (black), **7a**-ASP1222 (blue) and **7a**–PHE1223 (brown). (**C**) The binding mode of compound **8a** (yellow) in the binding site of c-Met (cyan and green) and its binding pocket. (**D**) The hydrogen bond interactions of **8a**–MET1211 (black) and the ionic bond of **8a**–GLU1127 (red). (**E**) The binding mode of compound **12c** (yellow) in the binding site of c-Met (cyan and green) and its binding pocket. (**F**) The hydrogen bond interactions of **12c**–ASP1222 (black) and **12c**–PHE1223 (red and green).

Molecular dynamic simulations were performed on the complex of compounds **7a**, **8a** and **12c** when bound with c-Met. The binding mode of these compounds on the protein c-Met ATP binding domain was similar to that determined using docking. The RMSD of **7a** to the initial conformation tended to be stable after 30 ns and remained at 3.0 Å indicating that the whole system reached equilibrium (Figure S2A). The linear type-II inhibitors were deeply embedded into a narrow binding site and the A, B and C rings were surrounded by hydrophobic amino acids residues, including ILE1084, PHE1089, ALA1108, LEU1157, PRO1158, MET1160 and MET1211 (Figure 6A). Meanwhile the carbonyl group formed a hydrogen bond with the amino group of MET1160. The distances between the isothiourea and the surrounding residues was within 3.0 Å and generated several hydrogen bonds with ASP1204, ASN1209, ASP1222 and PHE1223. These hydrogen bonds did not stabilise until 30 ns had elapsed (Figure 6B), as did the conformation. Thus, it can be concluded that the hydrogen bond and the hydrophobic interaction worked together to sustain the stability of the complex.

The RMSD of **8a** remained at ~2.0 Å in 50 ns (Figure S2B), and its bonding pattern in the pocket is as shown in Figure 6C. The hydrophobic A, B and C rings were surrounded by hydrophobic residues including ILE1084, ALA1108, PHE1089, LEU1140, MET1160 and MET1211, meanwhile, the B-ring made contact with the MET1160. In addition, positively charged N atoms in the tetramethylthiourea group formed ionic bonds with GLU1127. We calculated the ionic bond and hydrogen bond distances, and found that they were stable at more than 5 Å (Figure 6D), and the interaction force was relatively weak and insufficient to allow molecular equilibrium. Hydrophobic interaction and π–π stacking of the compound with PHE1089 played an important role in maintaining conformational stability.

After 20 ns, **12c** gradually stabilised at ~2.3 Å (Figure S2C) and, as we can see in Figure 6E, the inhibitor was bound in an extended conformation that stretched from the kinase linker to the pocket, and it also exhibited van der Waals force and hydrophobic interaction with surrounding hydrophobic residues such as ALA1092, LEU1140, LEU1142, LEU1157 and VAL1201. Residues ASP1222 and PHE1223 around the molecule formed hydrogen bonds with the carbonyl group of the B-ring. At the same time, PHE1223 formed hydrogen bond with protonated N atom in the compound. These hydrogen bonds were not stable until 20 ns and moreover, the hydrogen bonding between the carbonyl group of the B-ring with ASP1222 and protonated N atom in piperidine with PHE1223 were very strong (Figure 6F).

## 3. Discussion

HGF/c-Met signalling pathway has many biological functions and can promote tumour proliferation, invasion, metastasis and angiogenesis. HGF is produced by mesenchymal cells, which is combined with epithelial cells of specific receptors c-Met to activate the receptor tyrosine activity. Activated c-Met activates its downstream signalling pathways, such as PI3K/AKT and MARK/ERK in cytoplasm by recruiting Gab1 and Gab2, promoting the growth, migration and morphological change of a variety of cell. The HGF/c-Met signalling pathway, as a new target for a variety of solid tumours, is considered to be the most promising therapeutic targets in recent years, and it has also become one of the hot spots of current research.

In this study, we designed and synthesised a series of BISAs consisting of isothiourea, tetramethylthiourea and other groups, guided by virtual screening. Biological activity experiments proved that many compounds, except a modified guanidine, exhibited c-Met inhibitory activity and potential antitumour activity. The IC_50_ values of compounds **3d**, **7a**, **7b**, **8a**, **8b**, **12c** and **12d** on EBC-1 cells were less than 20 μM, whereas Ebselen showed no inhibitory effect thereon. In the subsequent antitumour activity experiments on four cancer cell lines (SK-N-SH, DLD1, MDA-MB-231 and A549), we found those compounds with high activities to EBC-1 had strong inhibitory effects on other cancer cell lines (IC_50_ < 20 μM). However, by comparison, it was found that the antitumour activity of these compounds to various cells might have different mechanisms. The results of Western blot assay revealed that compounds **3d**, **7a**, **7b**, **8a**, **8b** and **12c** containing thiourea, tetramethylthiourea or nitrogen heterocyclic groups had a significant inhibitory effect on the autophosphorylation of c-Met and a certain inhibitory activity on its downstream pathway signalling proteins such as AKT and ERK. According to the simulation results, hydrophobic interaction plays an important role in the stability of molecular conformation, and the existence of a C-ring is required. Although the IC_50_ value of compound **3d** is excellent due to the introduction of isothiourea directly into the B-ring, and considering that **3b** and **4** are a little weaker, we suggest that modification on the C-ring of Ebselen is a better choice for the inhibition of c-Met phosphorylation. Docking simulations and molecular dynamics simulations suggested that the introduction of positively charged isothiourea and heterocyclic groups resulted in the formation of hydrogen bonds, ionic bonds, and cation-π interactions between the compounds and protein amino acid residues, thus enhancing the binding of the compounds to c-Met.

Moreover, the inhibitory effect of these compounds on other targets remains to be further studied. As we mentioned earlier, most of type-II inhibitors belong to nonspecific multi-kinase target inhibitors, which can inhibit a variety of targets, and the compounds we designed are more in line with the characteristics of these inhibitors. In addition, in survival inhibition of cancer cell lines, these compounds not only inhibited EBC-1 cell line, but also inhibited four other cancer cell lines in varying degrees, as we discussed in the Section 2.3.2. Therefore, we speculate that there may be other inhibitory targets. 

Many clinical type-II c-Met inhibitors have great similarities in scaffolding, but the structure of the compounds we designed is quite different. In this work, we started with molecular structure design, synthesised and carried out bioactivity experiments under the guidance of virtual screening and binding free energy calculation and explained the interaction between compounds and protein by molecular docking and molecular dynamics simulation. We hope that our research methods and results will provide a reference for the search for novel potential c-Met drugs.

## 4. Materials and Methods 

### 4.1. Virtual Screening and Binding Energy Calculations

Virtual screening was performed using MOE [30]. Compounds were drawn in Chem3D saved the format as mol2 and minimised using 10,000 steps of steepest minimisation in MOE. The binding site was assumed to be identical to that of inhibitors of the same type in the crystal structure. Both the ligand and the protein were protonated at physiological pH prior to docking. The X-ray crystal structures of the c-Met (PDB ID: 3U6I and 3ZZE) were downloaded from the RCSB PDB (http://www.rcsb.org). In consideration of the flexibility of the side chains of the residues at the binding site, the induced fit docking approach was applied in the docking studies. The produced conformation with the best score was selected for the analysis.

Binding free energy calculation and decomposition was performed using the MM/GBSA script in AMBER 16 as previously described. Briefly, twenty-five frames were extracted from the last 5 ns of the MD trajectory at regular time intervals. The binding energies calculated on the ten frames were averaged.

### 4.2. Chemical Synthesis

All chemicals and solvents were purchased from Aladdin Reagent Ltd. (Shanghai, China) and directly used without further purification. TLC was performed on precoated silica gel 60 F254 plates (E. Merck, Darmstadt, Germany). Flash column chromatography was performed using silica gel (200–300 mesh) purchased from MeiGao Ltd. (Qingdao, China). Melting points were determined with an X-4 digital micro melting point tester (Taike Ltd., Beijing, China) and were uncorrected. All newly synthesised compounds were characterised with ^1^H NMR, ^13^C NMR on a Jeol JNM-ECP 600 spectrometer with tetramethylsilane (Me_4_Si) as the internal standard, and chemical shifts were recorded as δ values in ppm. Abbreviations used: s = singlet; d = doublet; t = triplet; q = quartet; m = multiplet; and dd = double-doublet. Mass spectra were recorded on a Q-TOF Global mass spectrometer. The ^1^H and ^13^C NMR spectra data of new compounds were submitted in the supplementary material (Figures S3–S54).

**1a**~**b** were prepared according to the literature [31]. These molecular can be obtained from appropriate 2,2’-bisselanyldibenzoic acid which was prepared from the diazotisation of o-aminobenzoic acid with a basic solution of Na_2_Se_2_. The appropriate 2,2’-bisselanyldibenzoic acid was refluxed with thionyl chloride for 3 h in the presence of a catalytic amount of DMF, and the unreacted thionyl chloride was removed under reduced pressure. The residue was extracted three times with *n*-hexane and after hexane removal, the obtained yellow solid was dried with an oil pump for half an hour in yield of 45–55%.

*N*-methyl-4-nitroaniline derived from p-fluoronitrobenzene in the yield of 98%, and then it was treated with sodium hydrogen and 1,6-Dibromohexane in THF to obtained **5** in Scheme S2.

The starting material of **12b** can be prepared from *N*-methyl-4-nitroaniline and CbzCl in the presence of NaOH, THF and H_2_O 3:1 as solvent. Then, the obtained compound was dissolved in methanol and 3-fold the amount of iron powder and a few drops of hydrochloric acid were added, and the reaction was carried out at 80 °C for 3 h. A two-step reaction required silica gel column with a total yield of 50%. The raw materials 1-(3-bromopropyl) piperidine for **12c** which was previously reported was made by 1,3-dibromopropane and piperidine in the yield of 55%.

#### 4.2.1. General Procedure for Compounds **2a**~**d**

To a stirred solution of appropriate ingredients NH_2_(CH_2_)*_n_*Br (2.0 mmol) and an equimolar amount of trimethylamine (2.0 mmol) in THF was slowly added a THF solution of 1.2-fold the amount of compound **1a** or **1b** (2.4 mmol) using a syringe under nitrogen atmosphere. After stirring at room temperature for 5 h and monitored with TLC, the reaction solution was filtered, and the solvent was distilled off under reduced pressure. The residue was subjected to silica gel column chromatography to give the target compounds **2a**~**d**. (The ^1^H and ^13^C NMR spectra data of compounds **2a**~**d** were submitted in the supplementary material).

#### 4.2.2. General Procedure for Compounds **3a**~**d**

Compounds **2a**~**d** (1.0 mmol) were mixed with thiourea (8.0 mmol), respectively, in THF (20 mL) and refluxed overnight. The organic solvent was distilled off under reduced pressure, and the reaction residue was subjected to silica gel column chromatography to obtain compounds **3a**~**d**. (The ^1^H and ^13^C NMR spectra data of compounds **3a**~**d** were submitted in the supplementary material).

#### 4.2.3. Synthesis of Compound (**4**)

Compound **2b** (1.0 mmol) was mixed with tetramethylthiourea (8.0 mmol) in THF (20 mL) and refluxed overnight. The organic solvent was distilled off under reduced pressure, and the reaction residue was subjected to silica gel column chromatography to obtain compound **4**. (The ^1^H and ^13^C NMR spectra data of compound **4** was submitted in the supplementary material).

#### 4.2.4. Synthesis of Compound (**5**)

To a stirred solution of *N*-methyl-4-nitroaniline (2.0 mmol) in THF (50 mL) was added NaH (8.0 mmol) under 0 °C. After reacting at 0 °C for half an hour, we added Br(CH_2_)_6_Br and moved the solution to room temperature for 3 h. The mixture was extracted with water and ethyl acetate (3 × 20 mL), and after evaporating the organic solvent in vacuo, the residue was purified by column chromatography to provide **5**. (The ^1^H and ^13^C NMR spectra data of compound **5** was submitted in the supplementary material).

#### 4.2.5. General Procedure for Compounds **6a**~**b**

To a stirred solution of ingredients **5** (2.0 mmol) in methanol (40 mL) and concentrated hydrochloric acid (6 mL) was added iron powder (8.0 mmol) under 80 °C. After stirring for 3 h at 80 °C, the mixture was filtered, and the solvent was evaporated under reduced pressure. The reaction residue was subjected to preliminary purification on a silica gel column.

Add the crude products obtained in the above step and trimethylamine (1.0 mmol) to a two-neck bottle. Then, a THF solution of 1.2-fold the amount of compound **1a** or **1b** (1.2 mmol) was added to the reaction vessel using a syringe under a nitrogen atmosphere. After stirring for 5 h, and under constant monitoring with TLC, the reaction solution was filtered and the solvent was distilled off under reduced pressure. The residue was subjected to silica gel column chromatography to give the target compounds **6a**~**b** (Scheme S1). (The ^1^H and ^13^C NMR spectra data of compounds **6a**~**b** were submitted in the supplementary material).

#### 4.2.6. General Procedure for Compounds **7a**~**b**

Compounds **6a**~**b** (0.5 mmol) were mixed with thiourea (4.0 mmol), respectively, in THF (20 mL) and refluxed overnight. The organic solvent was distilled off in vacuo and the reaction residue was subjected to silica gel column chromatography to obtain compounds **7a**~**b**. (The ^1^H and ^13^C NMR spectra data of compounds **7a**~**b** were submitted in the supplementary material).

#### 4.2.7. General Procedure for Compounds **8a**~**b**

Compounds **6a** or **6b** (0.5 mmol) were mixed with tetramethylthiourea (4.0 mmol), respectively, in THF (20 mL) and refluxed overnight. The organic solvent was distilled off in vacuo and the reaction residue was subjected to silica gel column chromatography to obtain compounds **8a**~**b**. (The ^1^H and ^13^C NMR spectra data of compounds **8a**~**b** were submitted in the supplementary material).

#### 4.2.8. General Procedure for Compounds **9a**~**b**

The DCM mixed solution of NH_2_(CH_2_)*_n_*NH_2_ and *N*,*N*′-Di-Boc-thiourea was placed at room temperature and stirred for 30 min [43]. The solution was extracted with water and DCM (3 × 10 mL). The organic phase was washed with water and dried with anhydrous magnesium sulphate. After filtering and drying the solvent under reduced pressure, white solid was obtained (Scheme S2).

#### 4.2.9. General Procedure for Compounds **10a**~**c**

To a stirred solution of **9a**~**b** (1.0 mmol) and an equimolar amount of trimethylamine (1.0 mmol) in THF was slowly added to a THF solution of 1.0-fold the amount of compound **1a** or **1b** (1.0 mmol) using a syringe under nitrogen atmosphere. After stirring at room temperature for 5 h, and under constant monitoring with TLC, the reaction solution was filtered and the solvent was distilled off under reduced pressure. The residue was subjected to silica gel column chromatography to give the target compounds **10a**~**c**. (The ^1^H and ^13^C NMR spectra data of compounds **10a**~**c** were submitted in the supplementary material).

#### 4.2.10. General Procedure for Compounds **11a**~**c**

HCl (5 mL) was added to a solution of **10a**~**c** (0.5 mmol) in DCM (20 mL) and the reaction was stirred at room temperature for 3 h. Reaction was detected by TLC, and the solvent was evaporated under vacuum to get compounds **11a**~**c** as yellow viscous liquid. (The ^1^H and ^13^C NMR spectra data of compounds **11a**~**c** were submitted in the supplementary material).

#### 4.2.11. Synthesis of Compound (**12a**)

To a stirred solution of *N*^1^-methylbenzene-1,4-diamine that reduced from *N*-methyl-4-nitroaniline (1.5 mmol) and an equimolar amount of trimethylamine (1.5 mmol) in THF was slowly added to a THF solution of compound **1a** (1.0 mmol) using a syringe under nitrogen atmosphere. After stirring at room temperature for 5 h and monitored with TLC, the reaction solution was filtered, and the solvent was distilled off under reduced pressure. The residue was subjected to silica gel column chromatography to give the target compounds **12a**. (The ^1^H and ^13^C NMR spectra data of compound **12a** was submitted in the supplementary material).

#### 4.2.12. Synthesis of Compound (**12b**)

To a stirred solution of benzyl (4-aminophenyl) (methyl)Carbamate which reduced from benzyl methyl (4-nitrophenyl) carbamate (1.5 mmol) and an equimolar amount of trimethylamine (1.5 mmol) in THF was slowly added to a THF solution of compound **1a** (1.0 mmol) using a syringe under nitrogen atmosphere. After stirring for 5 h and monitored with TLC, the reaction solution was filtered, and the solvent was distilled off under reduced pressure. The residue was subjected to silica gel column chromatography to give the target compounds **12b**. (The ^1^H and ^13^C NMR spectra data of compound **12b** was submitted in the supplementary material).

#### 4.2.13. Synthesis of Compound (**12c**)

A flask charged with **12a** (0.5 mmol) was dissolved in anhydrous THF (30 mL). K_2_CO_3_ (2.0 mmol) was then added into the solution, and after 15 min of continuous reaction 1-(3-bromopropyl) piperidine (1.0 mmol) was added. The reaction was carried out at room temperature overnight. The target product was obtained in 40% yield after column chromatography. (The ^1^H and ^13^C NMR spectra data of compound **12c** was submitted in the supplementary material).

#### 4.2.14. Synthesis of Compound (**12d**)

To a stirred solution of histamine dihydrochloride (1.5 mmol) and an equimolar amount of trimethylamine (1.5 mmol) in THF was slowly added a THF solution of compound **1a** (1.0 mmol) using a syringe under nitrogen atmosphere. After stirring at room temperature for 5 h and monitored with TLC, the reaction solution was filtered, and the solvent was distilled off under reduced pressure. The residue was subjected to silica gel column chromatography to give the target compounds **12d**. (The ^1^H and ^13^C NMR spectra data of compound **12d** was submitted in the supplementary material).

### 4.3. Resazurin Assay

Cells were seeded into 96-well cell culture plates and allowed to grow overnight. After that, the cells were treated with vehicle control (DMSO) or the compounds at the indicated concentration for 72 h. After treatment, 10 μL resazurin were added to the culture medium. After incubation at 37 °C for 5 h, fluorescence intensity was measured at a 530 nm excitation wavelength and a 590 nm emission wavelength using a Synergy HT photometer (BioTek, Burlington, VT, USA).

### 4.4. Western Blot

Antibodies against phospho-Met (Tyr1234/1235), phospho-AKT(Ser473), phospho-p44/42 MAPK (ERK1/2) (Thr202/Tyr204) and GAPDH (D16H11) were obtained from Cell Signalling Technology (Boston, MA, USA). Antibodies against α-Tubulin were acquired from Santa Cruz (Dallas, TX, USA). Cells were harvested in RIPA buffer (Cell Signalling Technology). Protein lysates were separated by SDS-PAGE, transferred to nitrocellulose membranes (GE Healthcare, Beijing, China), probed with first antibodies and then secondary antibodies. Immune complexes were detected by Immobilon Western Chemiluminescent HRP Substracte (Millipore, Shanghai, China) and photographed using S100 (Tanon, Shanghai, China).

### 4.5. Molecular Docking

Both compounds and protein were optimised through Schrödinger Suite 2017, and molecular docking was performed by using LeDock (http://www.lephar.com/software.htm). Firstly, compounds **7a**, **7b**, **8a**, **8b** and **12c** listed in Scheme 1 were sketched in ChemDraw and optimised through LigPrep module in Schrödinger Suite 2017. The crystal structure of c-Met in complex with an inhibitor (PDB code: 3U6I) revealed the protein kinase domain of c-Met and had a complete binding pocket and relatively high resolution, so that it was retrieved from Protein Data Bank (http://www.rcsb.org), then subjected to preprocessing, H-bond assignment, water removal and restrained minimisation in sequence by applying Protein Preparation Wizard module in Schrödinger Suite 2017, and finally processed through *CHARMM-GUI*. In this way, it was ensured that the protein structure without the original ligand was not only complete and but also ready for molecular docking. After molecular docking performed by LeDock, the optimal generated poses of the compounds were manually selected to be combined with the protein to generate 2D interaction diagrams in Figure 5 by the application of Ligand Interaction Diagram module in Schrödinger Suite 2017.

### 4.6. Molecular Dynamics Simulation

Molecular dynamic (MD) simulations were implemented on the complex of the protein c-Met and compound **7a**, **8a** and **12c**, respectively, using the Amber 16 package. The force field applied for the ligand was GAFF and FF14SB was for the receptor, respectively. The complex was solvated into a decagon box of TIP3P water molecules and neutralised using 4 Cl^−^ ahead of the MD simulations. The system was minimised to remove unfavourable van der Waals interactions, and MD was performed after minimisation. First, the solute was restrained and the whole system was gradually heated from 10 to 300 K in 100 ps in the NVT ensemble. Then the system was equilibrated in the NPT ensemble where the temperature and pressure were kept at 300 K and 1 atm, respectively. Finally, in the production process, the whole system was unconstrained and a 50 ns molecular dynamics process was carried out. For all MD simulations the time step was set to 2 fs, the particle mesh Ewald (PME) method [44] was applied to account for long-range electrostatic interactions and the lengths of the bonds involving hydrogen atoms were fixed with the SHAKE algorithm [45]. 

## 5. Conclusions

In this study, we designed and synthesised a series of BISAs consisting of isothiourea, tetramethylthiourea and other groups, guided by virtual screening. It was found that some compounds exhibited potential c-Met inhibitory activity and antitumour activity. Considering that there are no reports currently available on organic selenium compounds targeting c-Met, the introduction of isothiourea and some other heterocyclic groups in BISAs not only improved their water solubility and bioactivity, but also provided guidance to those searching for lead compounds of organic selenium that can inhibit c-Met. We hope that it can be used as guidance for the search of c-Met small molecule inhibitors with novel structures.

## Figures and Tables

**Table 1:**
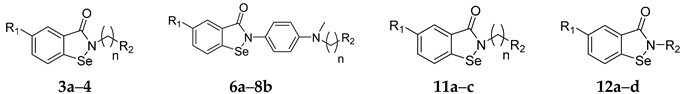
IC_50_ of the compounds in an EBC-1 cancer cell line.

Compound	R1	*n*	R2	IC_50_ of Cell Viability (μM)
**3a**	H	2	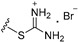	>25
**3b**	H	6	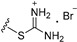	>25
**3c**	OMe	2	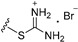	>25
**3d**	OMe	6	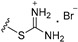	14.09
**4**	H	6	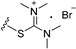	>25
**6a**	H	6	Br	>25
**7a**	H	6	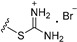	14.31
**7b**	OMe	6	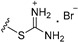	17.29
**8a**	H	6	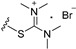	13.84
**8b**	OMe	6	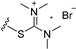	16.79
**11a**	H	2	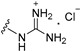	>50
**11b**	H	6	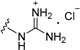	>50
**11c**	OMe	2	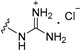	>50
**12a**	H	/	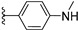	>25
**12b**	H	/	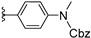	>25
**12c**	H	/	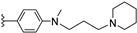	14.82
**12d**	H	/	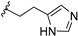	17.28

**Table 2:** IC_50_ of compounds (**3d**, **7a**, **8a**, **8b** and **12c**) in four cancer cell lines and EBC-1.

Compound	IC_50_ values of cell viability (μM)				
	SK-N-SH	DLD1	MDA-MB-231	A549	EBC-1
**3d**	26.55	20.61	23.18	14.91	14.09
**7a**	14.83	14.95	15.50	28.84	14.31
**8a**	11.51	16.48	16.41	45.68	13.84
**8b**	17.32	28.84	31.75	101.6	16.79
**12c**	13.86	14.91	22.63	23.19	14.82

## Supplementary Materials

Supplementary materials can be found at https://www.mdpi.com/1422-0067/20/10/2489/s1. Table S1: Structure and docking results of 300 BISAs compounds and Ebselen; Table S2: Binding free energy calculation and decomposition using the MMGBSA script in AMBER 16; Scheme S1: Synthesis of compounds **6a**~**6b**; Scheme S2: Synthesis of compounds **9a**~**9b**; Figure S1: The binding mode of known c-Met inhibitor cocrystallysed with the c-Met structure (3U6I) in two-dimensional panel; Figure S2: Evolution of the root-mean-square deviations (RMSD) of the c-Met protein bound with **7a**, **8a** and **12c** in 50 ns MD simulations; Analytical and spectral data for new compounds; Figures S3–S54: Copies of ^1^H and ^13^C NMR spectra of compounds **2a**~**d**, **3a**~**d**, **4**–**5**, **6a**~**b**, **7a**~**b**, **8a**~**b**, **10a**~**c**, **11a**~**c** and **12a**~**d**.

## Abbreviations

HGF	Hepatocyte growth factor
c-Met	Cellular mesenchymal–epithelial transition factor
RON	Recepteur d’Origine Nantais
TK	Tyrosine kinase
TKIs	Tyrosine kinase inhibitors
BISAs	Benzisoselenazolones
FAK	Focal Adhesion Kinase
AKT	Protein kinase B
ERK	Extracellular regulated protein kinases
ATP	Adenosine triphosphate
THF	Tetrahydrofuran
DCM	Dichloromethane
DMSO	Dimethyl sulphoxide
RMSD	Root mean square deviation
