# Supplementary material for: Virtual Screening Guided Design, Synthesis and Bioactivity Study of Benzisoselenazolones (BISAs) on Inhibition of c-Met and Its Downstream Signalling Pathways
Source: Int J Mol Sci. 2019 May 20;20(10):2489. doi: 10.3390/ijms20102489 (PMC6566228; doi:10.3390/ijms20102489)
Supplement: Supplementary file 1 [file ijms-20-02489-s001.pdf]

Supplementary Material

Virtual screening guided design, synthesis, and bioactivity study of BISAs on inhibition of c-Met and its downstream signalling pathways

Table S1. Structure and docking results of 300 BISAs compounds and Ebselen.

Number	Structure in MOE	Scores	
		3U6I	3ZZE
Ebselen	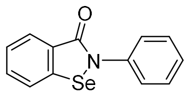	-6.1458	-5.9608
1	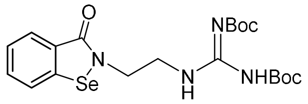	-8.9999	-8.3928
2	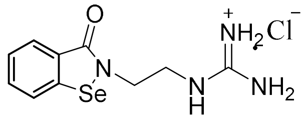	-6.3305	-6.1496
3	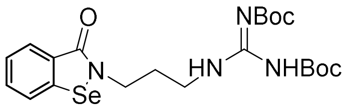	-8.602	-8.735
4	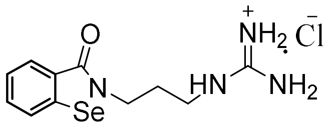	-6.4521	-6.2689
5	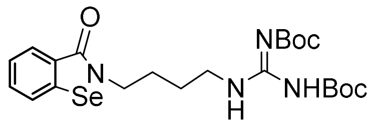	-8.8676	-8.8082
6	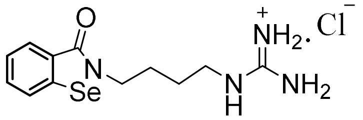	-6.7571	-6.4946
7	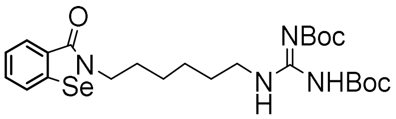	-9.379	-9.8461
8	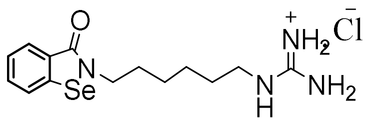	-6.9434	-7.1115
9	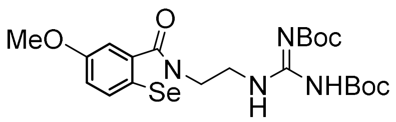	-8.8069	-8.9007

10	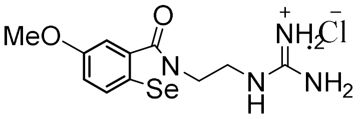	-6.7991	-6.7491
11	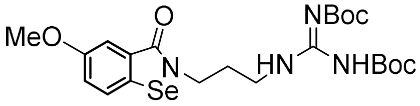	-8.9715	-9.1441
12	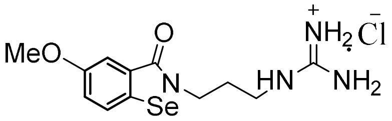	-7.1323	-6.9518
13	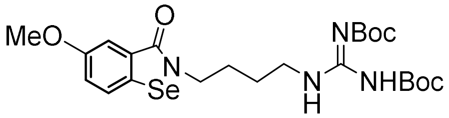	-9.4051	-9.3815
14	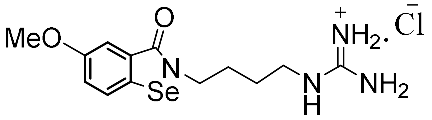	-6.8805	-6.3953
15	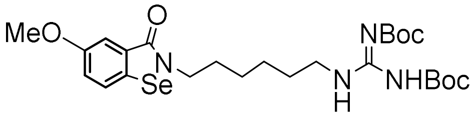	-9.6853	-9.7483
16	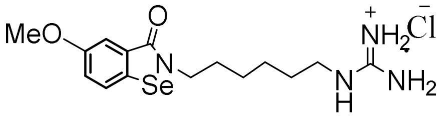	-7.9434	-7.4818
17	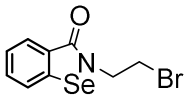	-5.9432	-5.8437
18	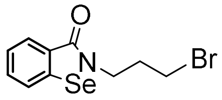	-6.2099	-6.2001
19	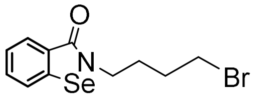	-6.535	-6.4084
20	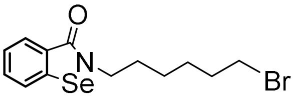	-7.0898	-6.9632
21	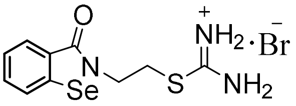	-6.5435	-6.2478
22	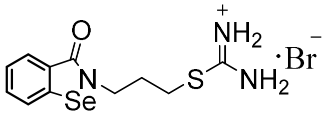	-6.7111	-6.651
23	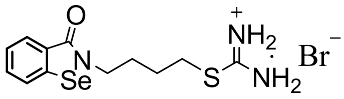	-6.8788	-6.7246

24	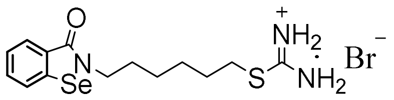	-7.4165	-7.1454
25	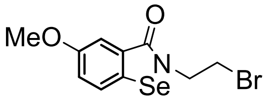	-6.323	-6.5084
26	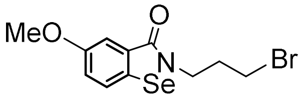	-6.6846	-6.6301
27	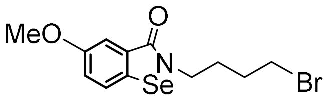	-6.8104	-7.1264
28	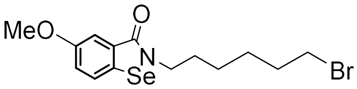	-7.4474	-7.4294
29	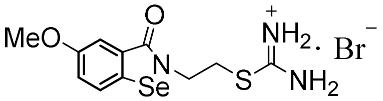	-6.8822	-7.1336
30	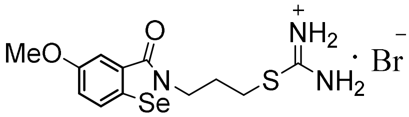	-7.2345	-7.1225
31	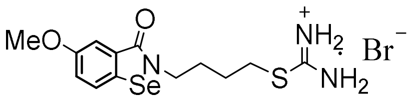	-7.3763	-7.1247
32	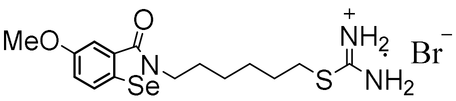	-8.0286	-7.8087
33	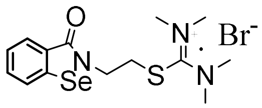	-7.5658	-7.2053
34	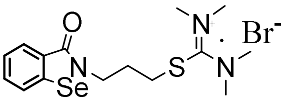	-8.004	-7.6662
35	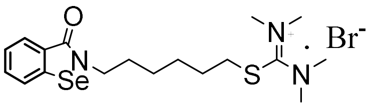	-8.7108	-8.1956
36	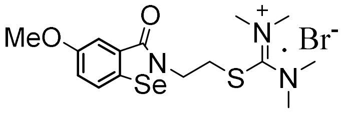	-8.2734	-7.9633
37	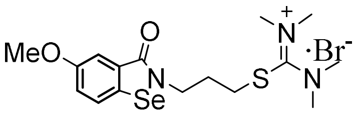	-8.4184	-7.5127

38	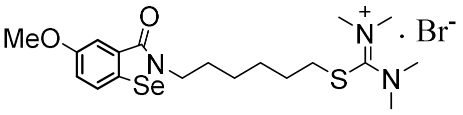	-8.9525	-8.9512
39	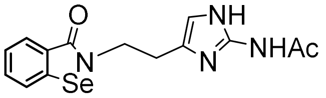	-7.6058	-7.3357
40	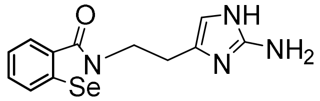	-6.8161	-6.5064
41	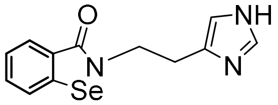	-6.6782	-6.2824
42	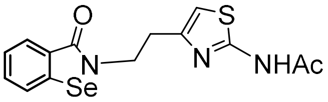	-7.3962	-7.5718
43	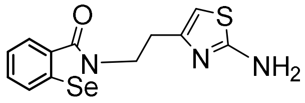	-6.8814	-6.5217
44	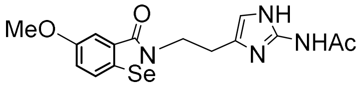	-7.7893	-7.7489
45	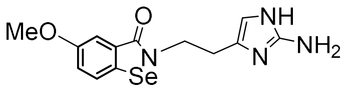	-7.3814	-7.2171
46	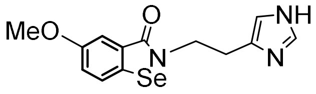	-7.1584	-6.7993
47	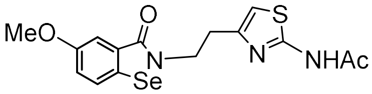	-8.1724	-7.5869
48	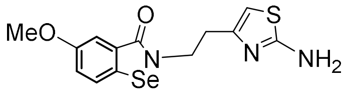	-7.1726	-7.1093
49	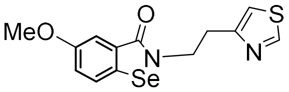	-7.1708	-7.0021
50	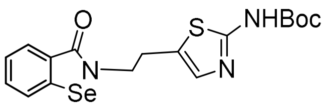	-8.3649	-7.8601
51	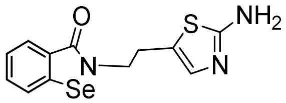	-6.8303	-6.6465

52	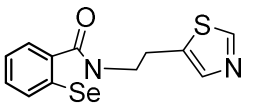	-6.6914	-6.3516
53	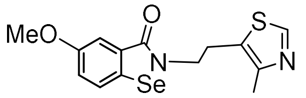	-8.5664	-7.8869
54	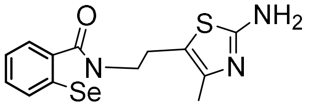	-7.0927	-6.9791
55	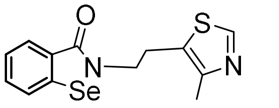	-7.0586	-6.844
56	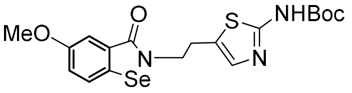	-8.5	-8.1627
57	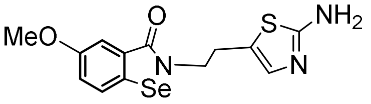	-7.3793	-7.0927
58	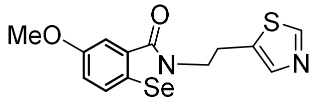	-7.0809	-7.0137
59	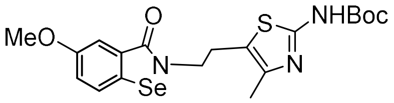	-8.7852	-8.1407
60	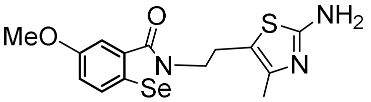	-7.4031	-7.6642
61	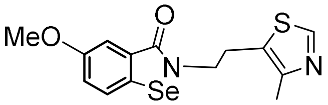	-7.4504	-7.2644
62	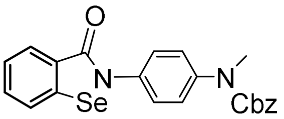	-8.6114	-8.0218
63	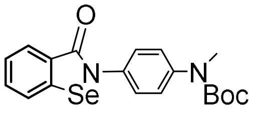	-8.1202	-7.5493
64	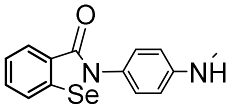	-6.5436	-6.3148
65	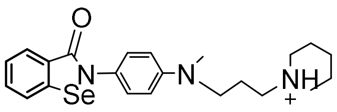	-9.0012	-7.6649

66	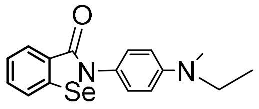	-7.1641	-6.7902
67	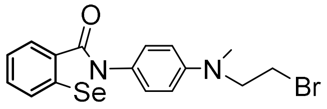	-7.4368	-6.9729
68	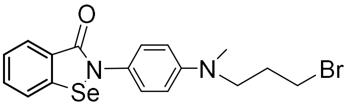	-7.7293	-7.4698
69	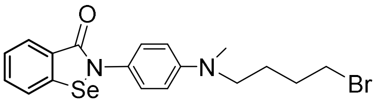	-7.8597	-7.893
70	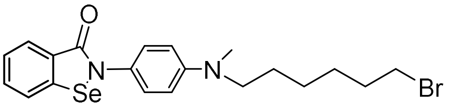	-8.4934	-8.0367
71	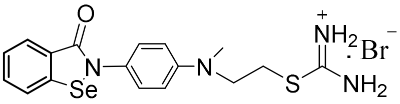	-7.7939	-7.2604
72	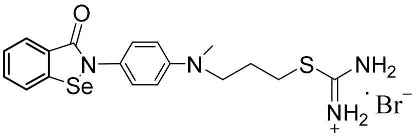	-8.1845	-7.8381
73	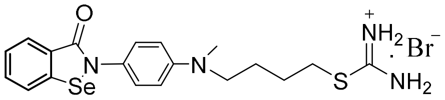	-8.6254	-8.125
74	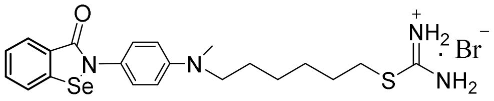	-8.9799	-8.3684
75	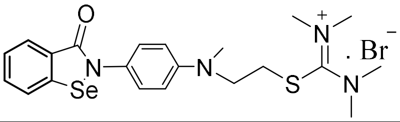	-9.0213	-8.626
76	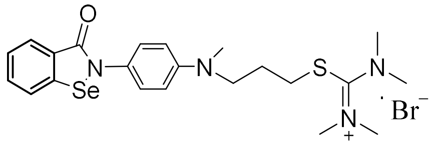	-9.6469	-8.983
77	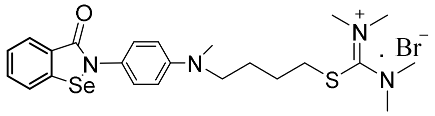	-9.8018	-9.002
78	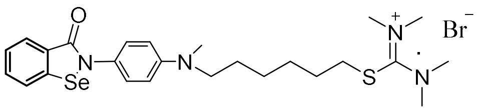	-9.9109	-8.6073
79	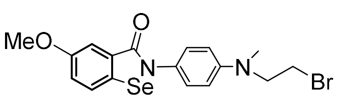	-7.8772	-7.4761

80	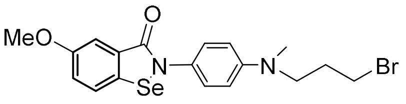	-8.3347	-7.5228
81	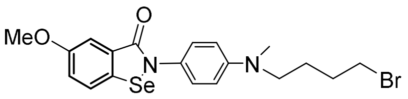	-8.4679	-7.9495
82	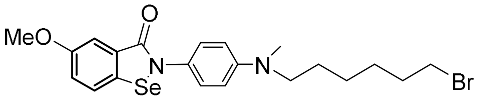	-8.9819	-8.475
83	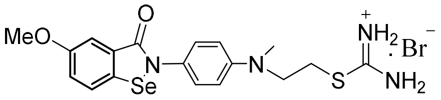	-8.1215	-7.9868
84	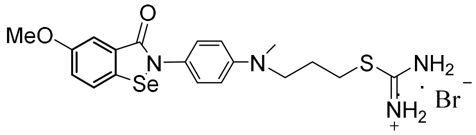	-8.4919	-8.141
85	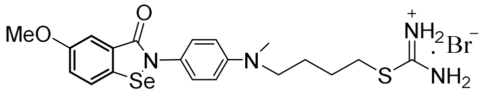	-9.1006	-8.1858
86	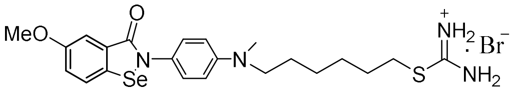	-9.1712	-8.7091
87	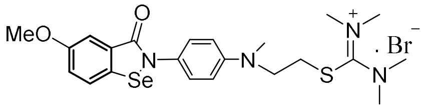	-9.6775	-8.3543
88	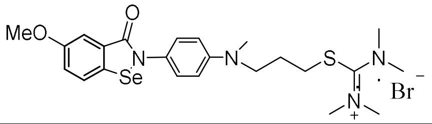	-9.9083	-9.2171
89	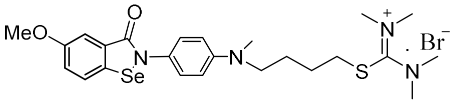	-10.069	-9.2565
90	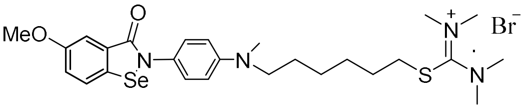	-10.1153	-9.7788
91	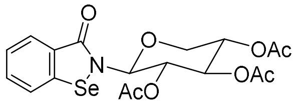	-8.7214	-8.6015
92	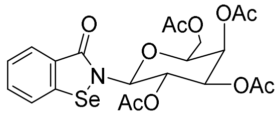	-8.5688	-9.5336
93	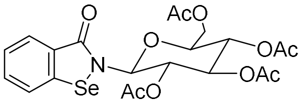	-8.7446	-9.3291

94	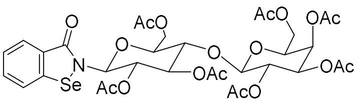	-11.111	-10.7897
95	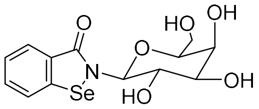	-6.9715	-7.3884
96	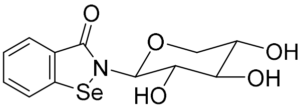	-7.0311	-6.8375
97	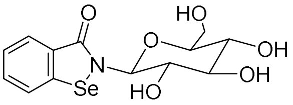	-7.3336	-7.2154
98	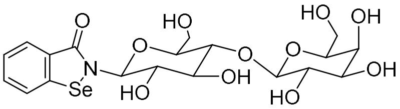	-9.3951	-9.2027
99	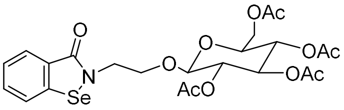	-9.4042	-10.2245
100	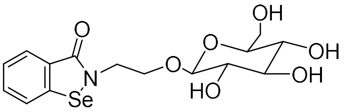	-7.5503	-7.0671
101	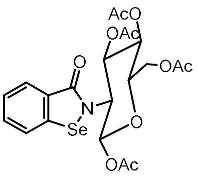	-8.084	-7.7107
102	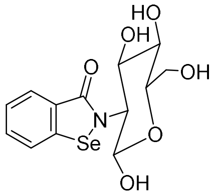	-6.6668	-6.4157
103	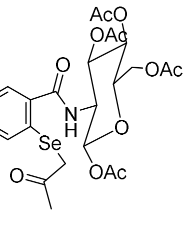	-9.8637	-9.3388
104	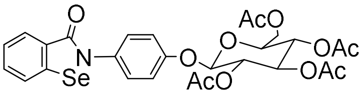	-9.3604	-8.4963
105	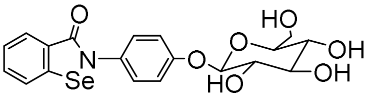	-8.6033	-8.0493

106	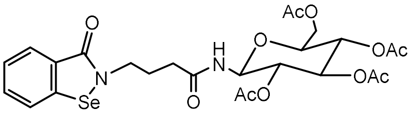	-8.7528	-8.3836
107	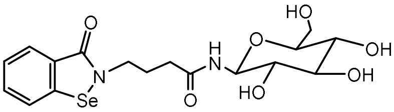	-8.0861	-7.5963
108	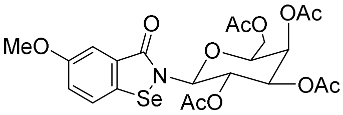	-8.0884	-8.0639
109	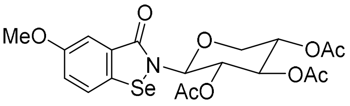	-7.6045	-7.5911
110	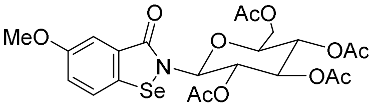	-7.3662	-8.4542
111	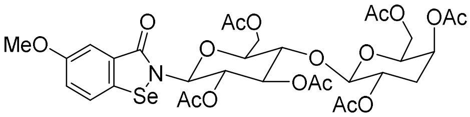	-8.5206	-8.4613
112	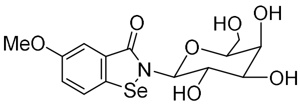	-7.3483	-7.7277
113	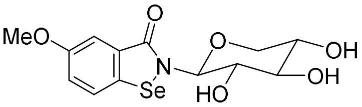	-7.0681	-6.583
114	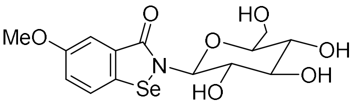	-7.6229	-7.7006
115	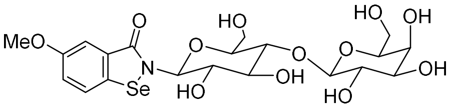	-9.8637	-8.9304
116	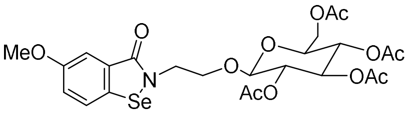	-9.1284	-8.6102
117	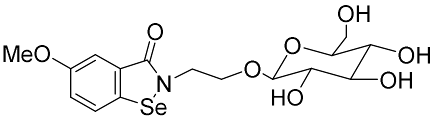	-8.2538	-7.6573
118	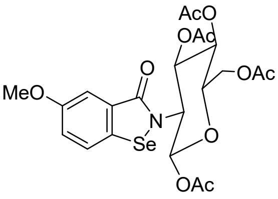	-7.8845	-8.0896

119	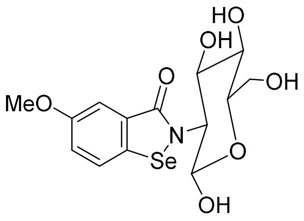	-7.4118	-6.6782
120	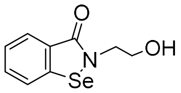	-5.5328	-5.3425
121	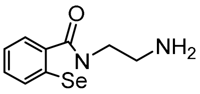	-5.2838	-5.4243
122	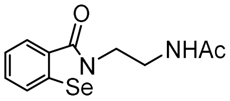	-6.4263	-6.1402
123	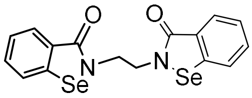	-7.4037	-6.5155
124	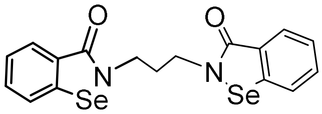	-7.7802	-6.9626
125	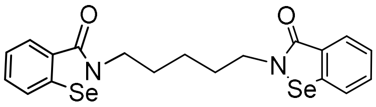	-8.0855	-7.1305
126	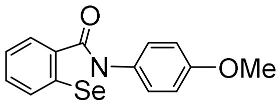	-6.2801	-6.1155
127	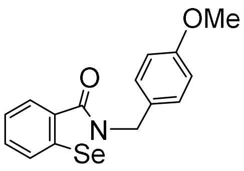	-6.7913	-6.2652
128	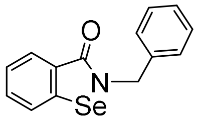	-6.5296	-6.4983
129	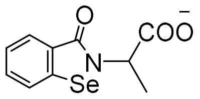	-6.0265	-5.7895
130	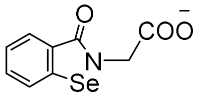	-5.616	-5.568
131	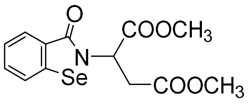	-7.1448	-7.05

132	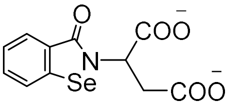	-6.3471	-6.629
133	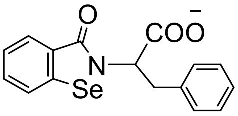	-6.9216	-7.1127
134	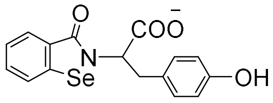	-7.2191	-7.4741
135	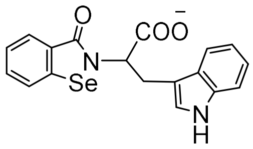	-7.3736	-7.098
136	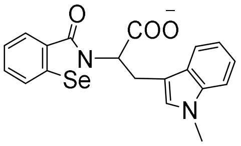	-7.0311	-7.1617
137	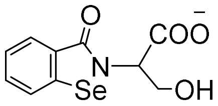	-6.3359	-6.1675
138	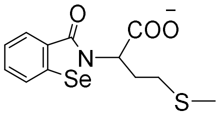	-6.7441	-7.0555
139	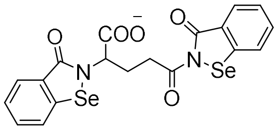	-8.0953	-7.1502
140	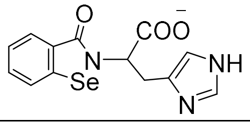	-6.6781	-6.5631
141	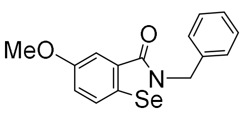	-6.9174	-7.0623
142	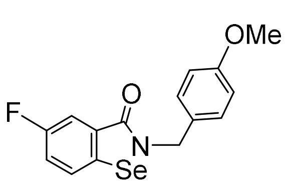	-6.8709	-6.7003
143	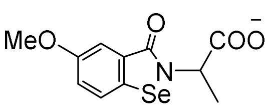	-6.1289	-6.0706
144	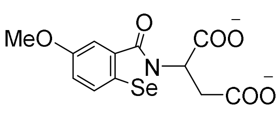	-6.4107	-6.4666

145	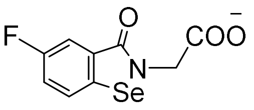	-5.5059	-5.6643
146	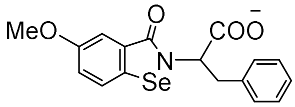	-7.139	-7.0798
147	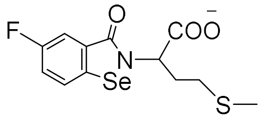	-6.7413	-6.8907
148	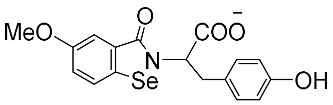	-7.3295	-7.5631
149	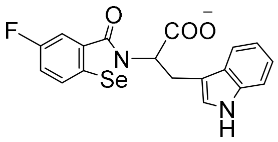	-7.3698	-7.055
150	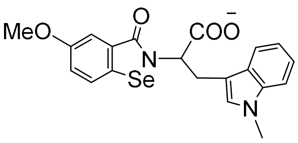	-7.7695	-7.411
151	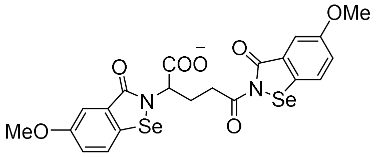	-9.0413	-8.4507
152	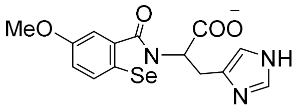	-7.168	-6.9414
153	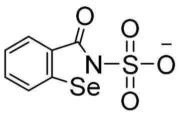	-4.9319	-4.8986
154	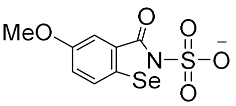	-5.5144	-5.3224
155	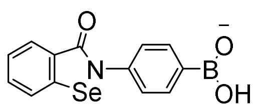	-6.777	-6.2242
156	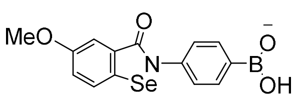	-7.4199	-6.4958
157	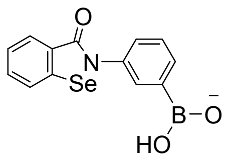	-6.3405	-6.3301

158	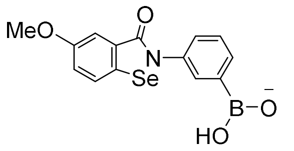	-6.8079	-6.9559
159	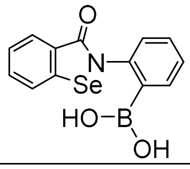	-6.8608	-6.7888
160	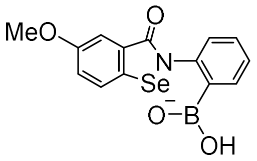	-6.6295	-7.062
161	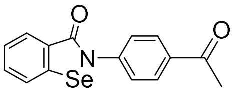	-6.6404	-6.1936
162	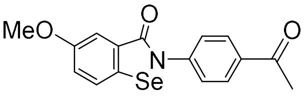	-6.924	-6.5806
163	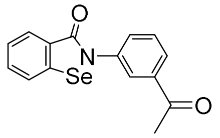	-6.6242	-6.3045
164	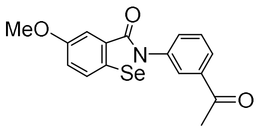	-7.0357	-6.6989
165	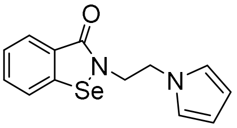	-6.3027	-6.6955
166	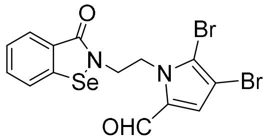	-6.9527	-6.7661
167	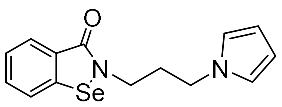	-6.9286	-6.8749
168	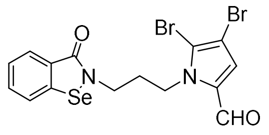	-7.2135	-7.1542
169	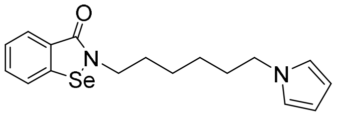	-7.3677	-6.9435

170	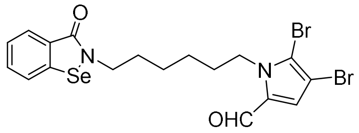	-8.1971	-7.3675
171	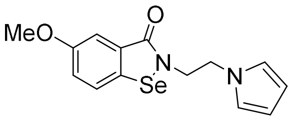	-6.6653	-7.0036
172	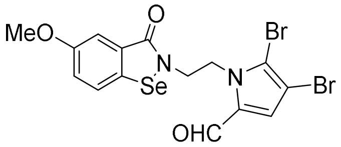	-7.5664	-7.2667
173	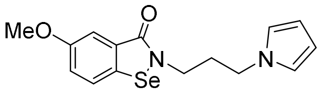	-7.0522	-6.8744
174	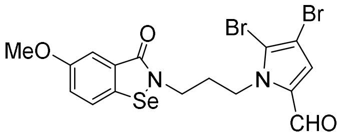	-7.9797	-7.2631
175	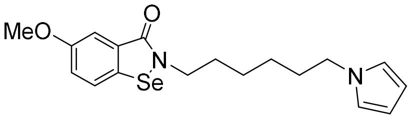	-7.8671	-7.5022
176	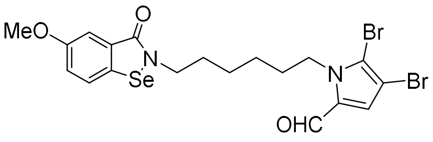	-8.741	-7.914
177	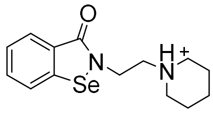	-6.6833	-6.1572
178	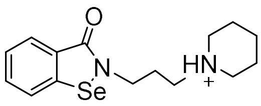	-7.0338	-6.6492
179	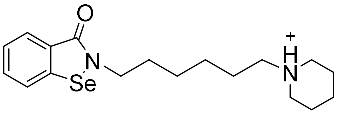	-8.0127	-7.265
180	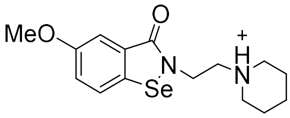	-7.0632	-6.9653
181	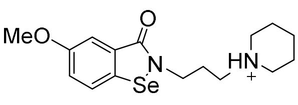	-7.5091	-7.2987
182	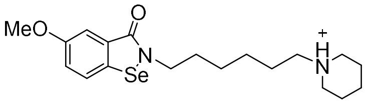	-7.5197	-6.8419

183	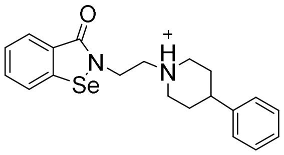	-7.6871	-7.1633
184	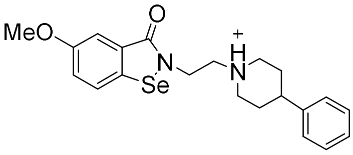	-7.9542	-7.4267
185	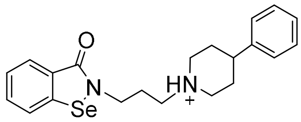	-8.067	-7.687
186	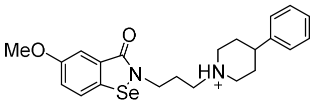	-8.5261	-7.5711
187	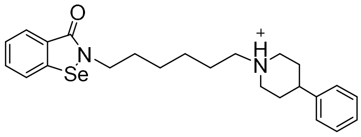	-8.9852	-8.0598
188	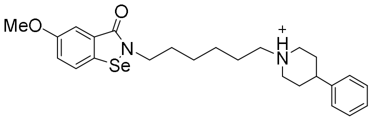	-9.6471	-7.6932
189	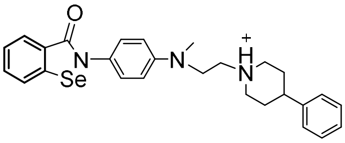	-9.5735	-7.4713
190	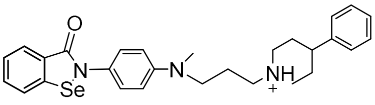	-9.372	-8.0108
191	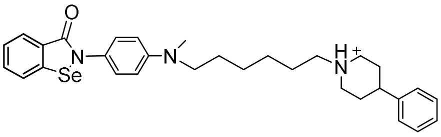	-10.5659	-8.1266
192	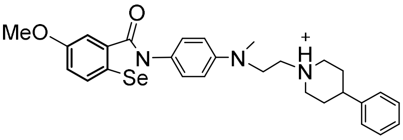	-9.925	-7.9789
193	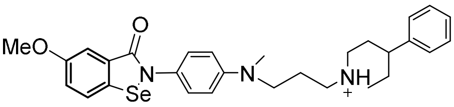	-9.7914	-8.2667
194	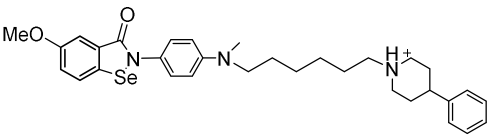	-10.1509	-7.9986

195	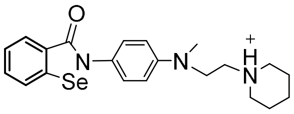	-8.1882	-7.124
196	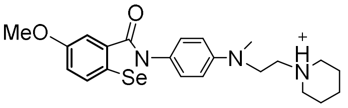	-8.4213	-7.8641
197	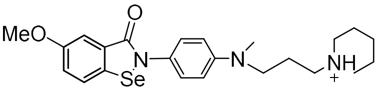	-8.2611	-7.6875
198	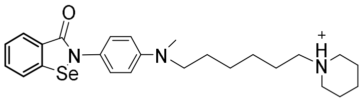	-9.1389	-7.6478
199	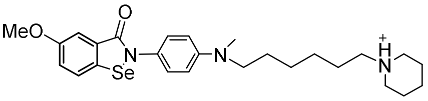	-9.4598	-8.5863
200	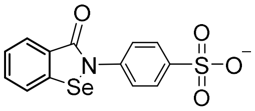	-6.3114	-5.6773
201	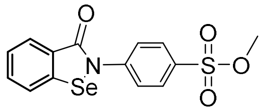	-7.0683	-6.8548
202	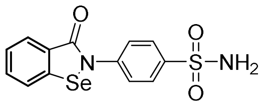	-6.8443	-6.3774
203	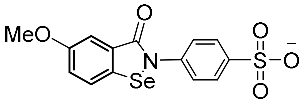	-7.1797	-6.5268
204	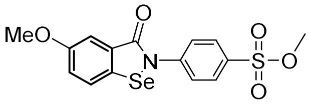	-7.4772	-7.1854
205	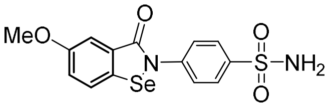	-7.1577	-6.6484
206	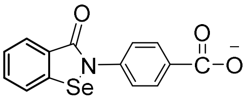	-6.4393	-6.0724
207	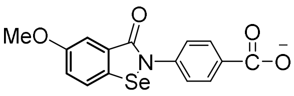	-7.0033	-6.6051
208	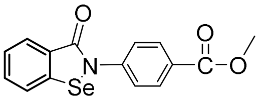	-7.0708	-6.7748

209	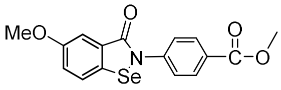	-7.4997	-7.1842
210	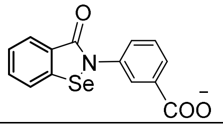	-6.4072	-6.4627
211	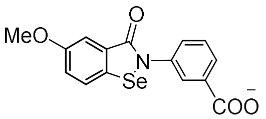	-6.9585	-6.5157
212	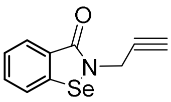	-5.8128	-5.8303
213	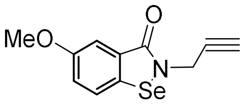	-6.2407	-6.1773
214	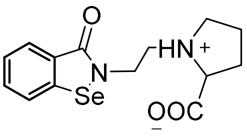	-7.4347	-6.6797
215	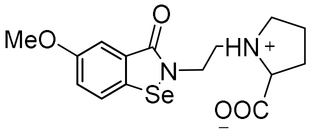	-7.8229	-7.0822
216	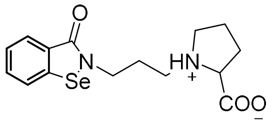	-7.6025	-6.9484
217	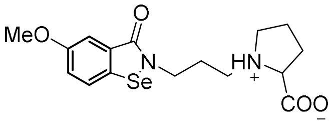	-7.7925	-7.3364
218	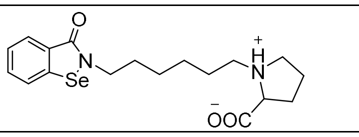	-8.2174	-7.8189
219	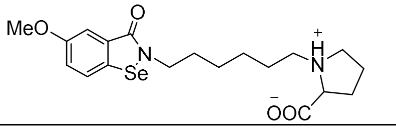	-8.522	-8.1796
220	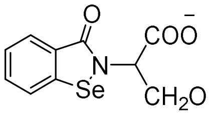	-6.1087	-5.9913
221	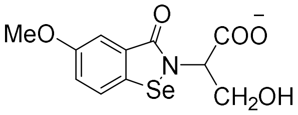	-6.5183	-6.5147

222	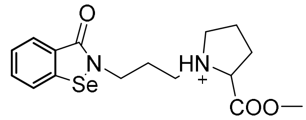	-7.7573	-7.3245
223	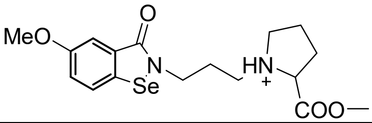	-7.9226	-8.168
224	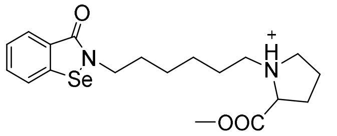	-8.3929	-8.2151
225	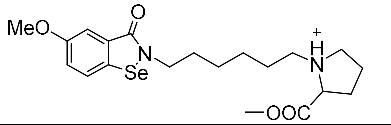	-9.1766	-8.7376
226	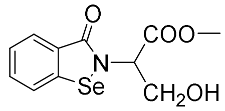	-6.688	-6.5551
227	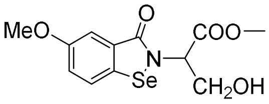	-7.1773	-6.9376
228	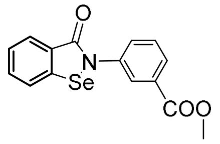	-6.9872	-6.9873
229	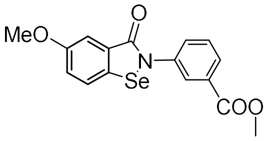	-7.2931	-7.3824
230	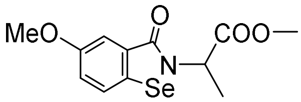	-6.9297	-6.6167
231	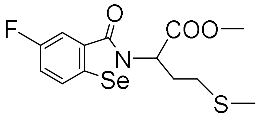	-7.318	-7.1968
232	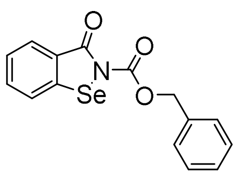	-7.0158	-6.8988
233	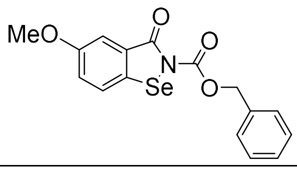	-7.557	-7.4935

234	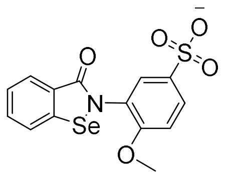	-7.4289	-6.6886
235	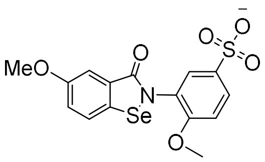	-7.4949	-7.0698
236	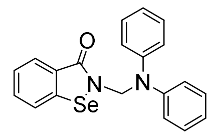	-7.3507	-7.1865
237	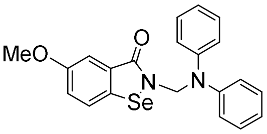	-7.7838	-7.3728
238	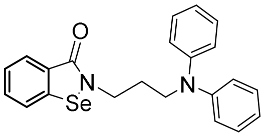	-8.2507	-7.8968
239	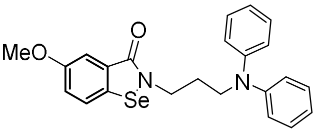	-8.7255	-8.2962
240	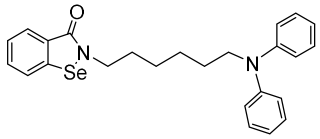	-9.1166	-8.4603
241	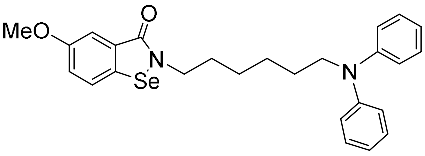	-9.34	-8.9282
242	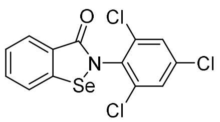	-6.1843	-6.5962
243	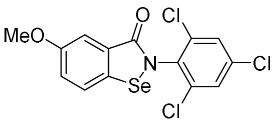	-7.3734	-7.1148
244	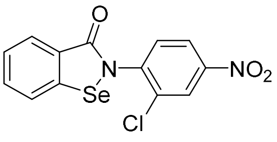	-7.0239	-6.5305

245	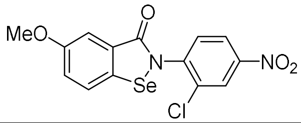	-7.4627	-7.3316
246	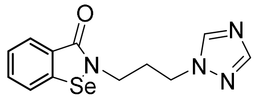	-6.9705	-6.7497
247	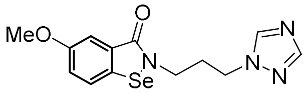	-7.2242	-7.1632
248	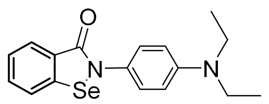	-7.246	-7.2319
249	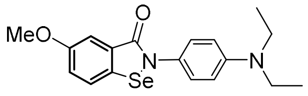	-7.8654	-7.5675
250	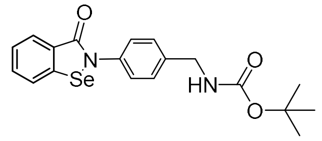	-7.9546	-7.8829
251	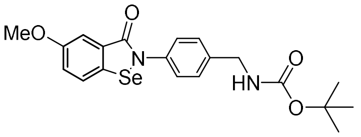	-8.4592	-7.876
252	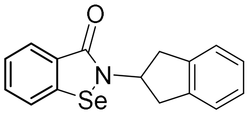	-6.9306	-6.3791
253	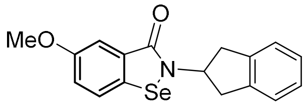	-7.435	-6.8736
254	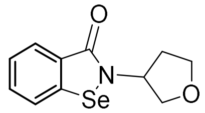	-6.1711	-6.1025
255	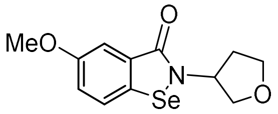	-6.6373	-6.519
256	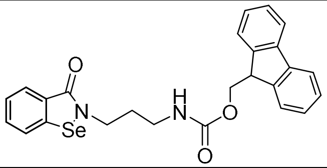	-9.2311	-8.145
257	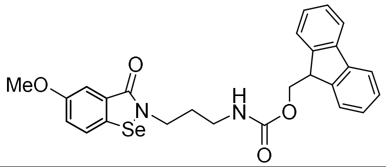	-9.4009	-8.6129

258	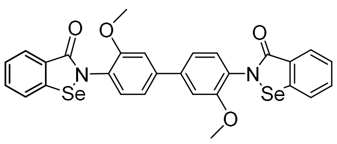	-10.8445	-8.8799
259	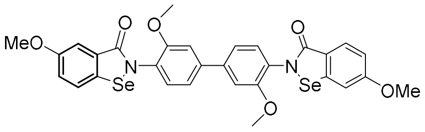	-10.016	-8.6649
260	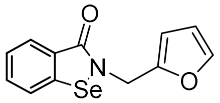	-6.4501	-6.0574
261	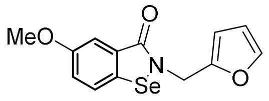	-6.8698	-6.5246
262	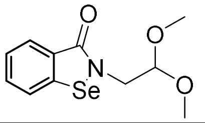	-6.519	-6.3981
263	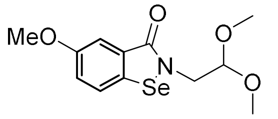	-7.0612	-6.8269
264	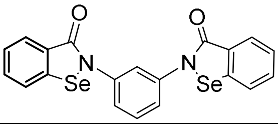	-8.259	-7.4505
265	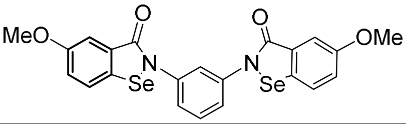	-8.9116	-8.4717
266	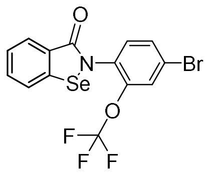	-7.2902	-7.0157
267	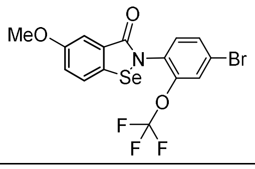	-7.8659	-7.015
268	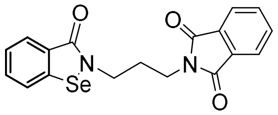	-7.6664	-7.5998
269	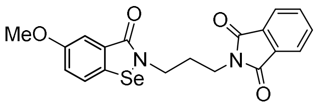	-8.1916	-8.0021
270	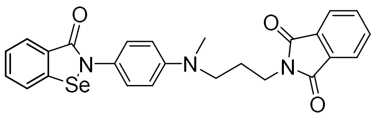	-9.3989	-8.324

271	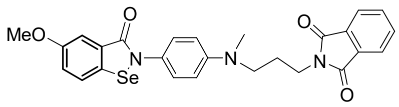	-9.5092	-8.5089
272	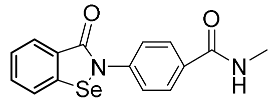	-7.1373	-6.787
273	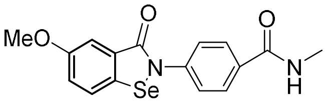	-7.7158	-7.6279
274	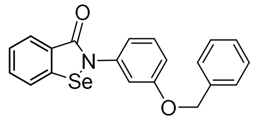	-7.7702	-7.4154
275	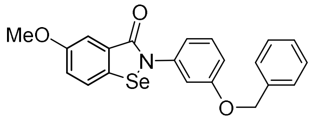	-8.2664	-7.9518
276	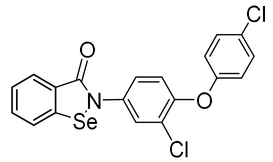	-8.0889	-7.2676
277	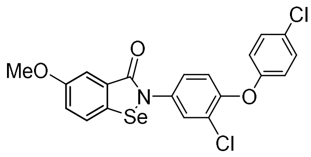	-8.5529	-7.8619
278	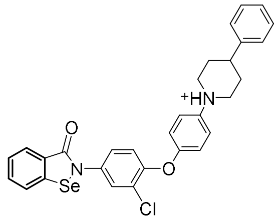	-9.2424	-8.6616
279	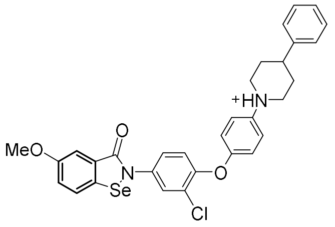	-9.5219	-8.7917
280	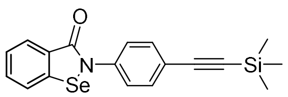	-7.7122	-7.2398
281	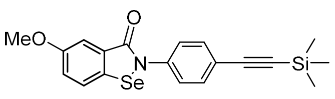	-7.8883	-7.8752

282	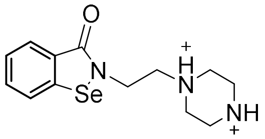	-7.4479	-6.6911
283	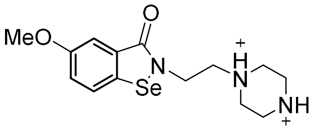	-7.7148	-7.2802
284	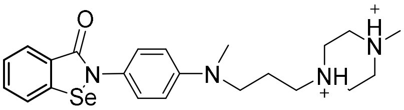	-8.7275	-8.3676
285	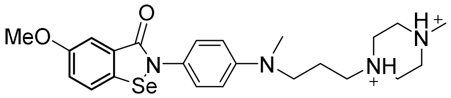	-9.2994	-7.9955
286	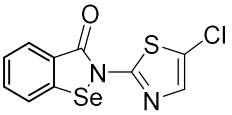	-6.3974	-6.2357
287	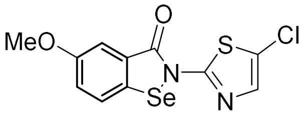	-6.8216	-6.3707
288	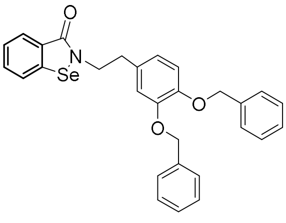	-9.4211	-8.6657
289	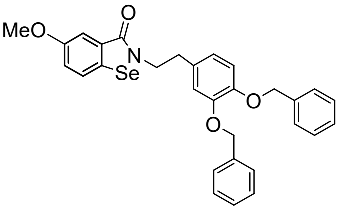	-9.5847	-9.4119
290	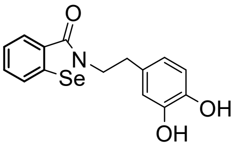	-7.1504	-6.8652
291	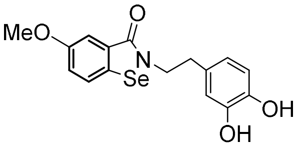	-7.6384	-6.9793

292	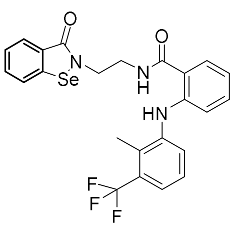	-8.9057	-8.4803
293	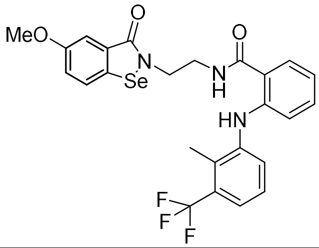	-9.5418	-9.167
294	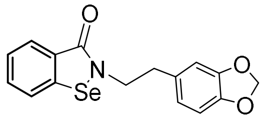	-7.2261	-6.9761
295	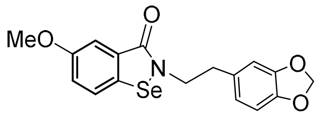	-7.7682	-7.5343
296	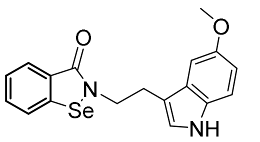	-7.7535	-7.404
297	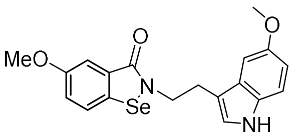	-8.3883	-7.9162
298	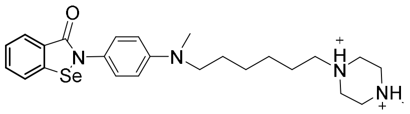	-9.5272	-8.7217
299	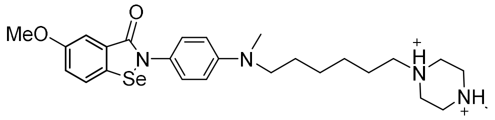	-9.9381	-9.3654
300	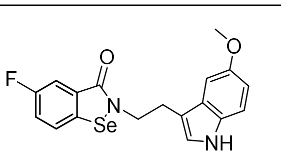	-7.844	-7.4391

Table S2. Binding free energy calculation and decomposition using the MMGBSA script in AMBER 16.

Compound Number	Binding free energy	Compound Number	Binding free energy
65	-50.1675±1.0149	75	-58.2660±0.8334
76	-41.1891±0.7926	77	-50.5297±0.8414
78	-44.1653±0.6886	85	-40.1798±1.0718
86	-59.1457±1.2310	87	-40.4153±1.0148
88	-50.9957±0.9054	89	-46.5625±1.6331
90	-47.8221±0.7948	94	-32.1107±0.9866
98	-28.3848±1.1840	99	-45.4442±1.0132
104	-46.5621±1.1826	116	-36.8738±0.7043
151	-42.5155±1.0010	188	-38.5763±0.8896
190	-39.2805±2.0498	191	-64.1102±1.1160
192	-61.4149±0.9947	193	-50.8160±1.2643
194	-60.2086±1.0741	198	-52.3320±0.7155
199	-59.8037±1.1250	225	-41.9554±0.7624
240	-44.8807±0.8251	241	-51.9235±0.9427
256	-55.3331±0.9213	257	-52.2890±0.8211
258	-60.3422±0.7868	259	-53.2528±0.8264
270	-52.8233±0.8234	271	-57.4254±0.9254
278	-44.0144±0.9275	279	-43.1069±0.6712
288	-46.7632±0.7423	289	-51.7130±0.6518
292	-44.9514±0.8639	293	-45.4408±0.8461

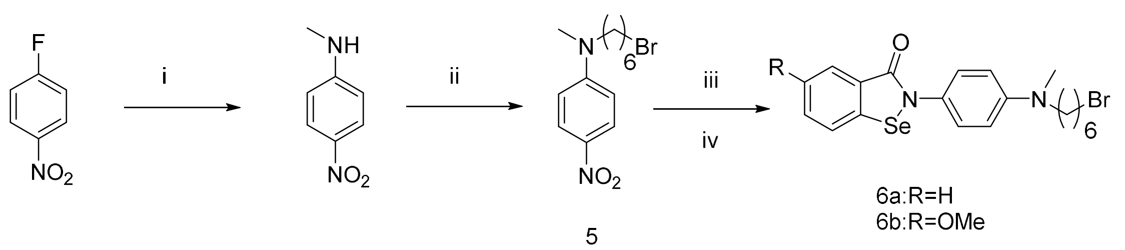

Scheme S1. Synthesis of compounds 6a~6b. Reagents and conditions: (i) Methylamine hydrochloride, K_2CO_3, DMSO, 85°C, overnight, 98%; (ii) $Br(CH_2)_6Br$, NaH, THF, 0°C ~ rt, 3h, 70%; (iii) Fe/HCl, Methanol, 80°C, 3h; (iV) **1a~b**, Et_3N, THF, rt, 5h, 35%-37% (2 steps)

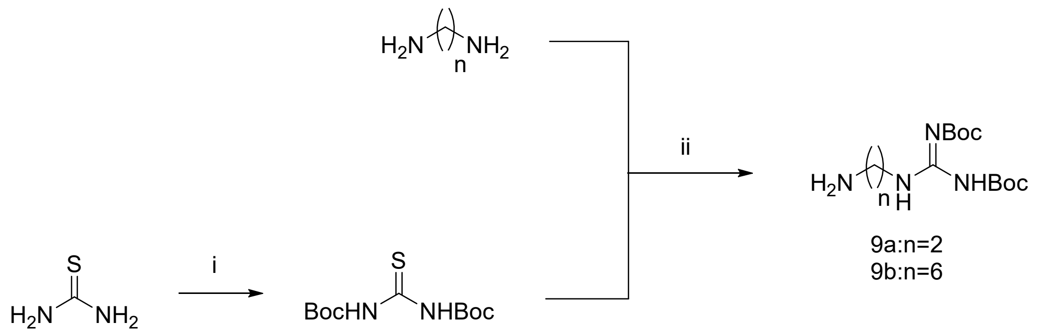

Scheme S2. Synthesis of compounds 9a~9b. Reagents and conditions: (i) $(Boc)_2O$, DCM, rt, 1h, 33%; (ii) DCM, rt, 30min, 60%-70%.

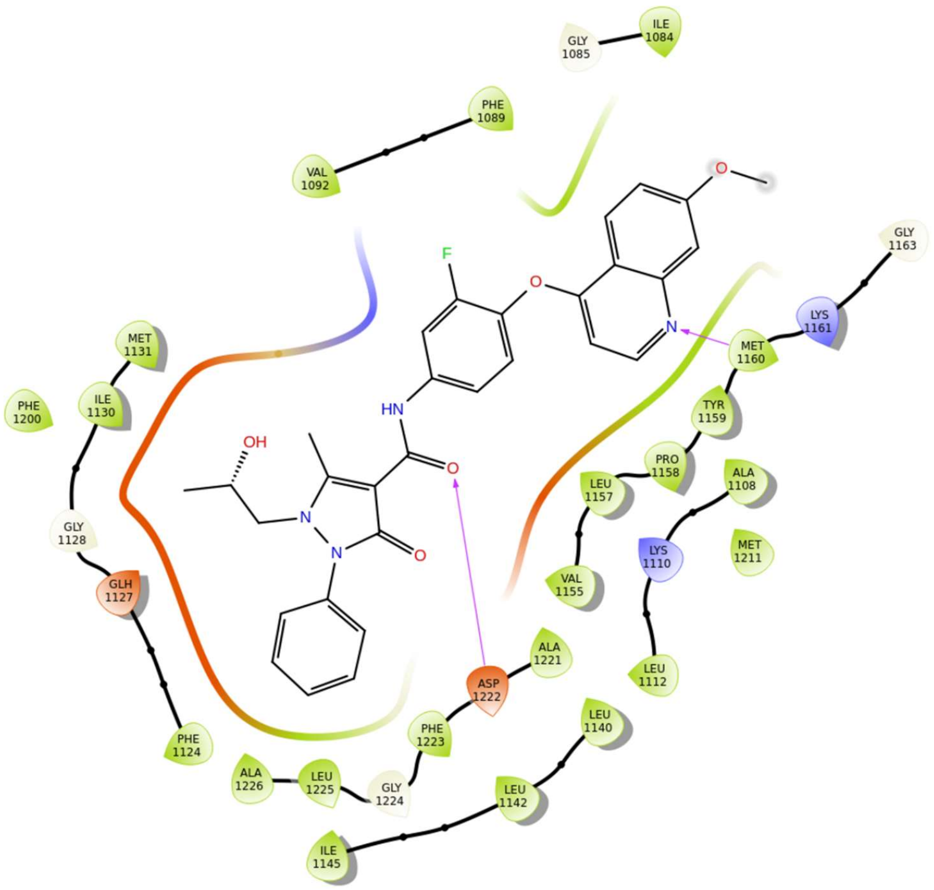

Fig S1. The binding mode of known c-Met inhibitor co-crystallyzed with the c-Met structure (3U6I) in two-dimensional panel. The purple arrow indicates the hydrogen bond.

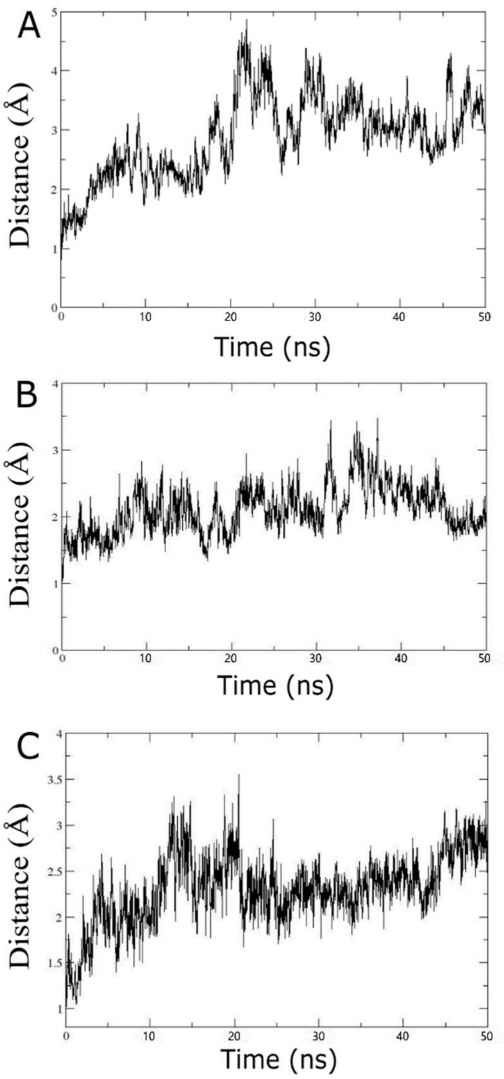

Fig S2. Evolution of the root-mean-square deviations (RMSD) of the c-Met protein bound with 7a, 8a and 12c in 50 ns MD simulations. A) RMSD of the c-Met protein bound with **7a**. B) RMSD of the c-Met protein bound with **8a**. C) RMSD of the c-Met protein bound with **12c**.

Analytical and spectral data for new compounds

2-(2-bromoethyl) benzo[d][1,2]selenazol-3(2H)-one (**2a**)

Yellow solid (340mg, yield 56%), mp 195-197 °C; ^{1}H NMR (500 MHz, $CDCl_3$) δ 8.07 (d, *J* = 7.8 Hz, 1H), 7.66 (d, *J* = 7.4 Hz, 1H), 7.64 – 7.60 (m, 1H), 7.47 – 7.42 (m, 1H), 4.24 (t, *J* = 6.3 Hz, 2H), 3.66 (t, *J* = 6.3 Hz, 2H); ^{13}C NMR (126 MHz, $CDCl_3$) δ 167.43, 138.30, 132.36, 128.91, 126.41, 126.35, 123.96, 46.60, 30.45; MS (ESI) m/z ESI^{+} 305.03 $[M+H]^{+}$.

2-(6-bromohexyl) benzo[d][1,2]selenazol-3(2H)-one (**2b**)

Yellow solid (360mg, yield 50%), mp 107-109 °C; ^{1}H NMR (500 MHz, $CDCl_3$) δ 8.03 (d, *J* = 7.9 Hz, 1H), 7.64 (d, *J* = 7.9 Hz, 1H), 7.60 – 7.55 (m, 1H), 7.41 (t, *J* = 7.4 Hz, 1H), 3.85 (t, *J* = 7.1 Hz, 2H), 3.39 (t, *J* = 6.8 Hz, 2H), 1.88 – 1.81 (m, 2H), 1.77 – 1.69 (m, 2H), 1.49 (m, *J* = 14.3, 7.0 Hz, 2H), 1.45 – 1.38 (m, 2H); ^{13}C NMR (126 MHz, $CDCl_3$) δ 167.17, 137.62, 131.90, 128.79, 127.50, 126.20, 123.99, 44.60, 33.75, 32.54, 30.32, 27.76, 25.72; MS (ESI) m/z ESI$^+$ 362.14 $[M+H]^+$.

2-(2-bromoethyl)-5-methoxybenzo[d][1,2]selenazol-3(2H)-one (**2c**)

Yellow solid (422mg, yield 63%), mp 230-232 °C; ^{1}H NMR (500 MHz, $CDCl_3$) δ 7.54 (d, *J* = 2.7 Hz, 1H), 7.50 (d, *J* = 8.7 Hz, 1H), 7.25 (dd, *J* = 8.7, 2.7 Hz, 1H), 4.22 (t, *J* = 6.4 Hz, 2H), 3.88 (s, 3H), 3.64 (t, *J* = 6.4 Hz, 2H); ^{13}C NMR (126 MHz, $CDCl_3$) δ 167.30, 159.00, 129.11, 127.42, 124.67, 122.35, 110.64, 55.75, 46.76, 30.36; MS (ESI) m/z ESI$^+$ 336.06 $[M+H]^+$.

2-(6-bromohexyl)-5-methoxybenzo[d][1,2]selenazol-3(2H)-one (**2d**)

Yellow solid (546mg, yield 70%), mp 110-112 °C; ^{1}H NMR (500 MHz, $CDCl_3$) δ 7.52 (d, *J* = 2.6 Hz, 1H), 7.49 (d, *J* = 8.7 Hz, 1H), 7.21 (dd, *J* = 8.7, 2.7 Hz, 1H), 3.87 (s, 3H), 3.85 (t, *J* = 7.1 Hz, 2H), 3.39 (t, *J* = 6.8 Hz, 2H), 1.88 – 1.81 (m, 2H), 1.74 (dd, *J* = 13.9, 6.5 Hz, 2H), 1.52– 1.46 (m, 2H), 1.44 – 1.38 (m, 2H); ^{13}C NMR (126 MHz, $CDCl_3$) δ 167.04, 158.90, 128.51, 128.40, 124.67, 121.85, 110.60, 55.71, 44.79, 33.74, 32.55, 30.31, 27.76, 25.70; MS (ESI) m/z ESI$^+$ 392.17 $[M+H]^+$.

2-(3-oxobenzo[d][1,2]selenazol-2(3H)-yl)ethyl carbamimidothioate hydrobromide (**3a**)

Yellow solid (179mg, yield 47%), mp 210-212 °C; ^{1}H NMR (500 MHz, DMSO-d_6) δ 9.11 (s, 4H), 8.33 – 8.24 (m, 1H), 7.81 (d, *J* = 7.6 Hz, 1H), 7.60 (t, *J* = 7.4 Hz, 1H), 7.42 (t, *J* = 7.3 Hz, 1H), 3.99 (t, *J* = 6.4 Hz, 2H), 3.45 (t, *J* = 6.5 Hz, 2H); ^{13}C NMR (126 MHz, DMSO-d_6) δ 169.94, 167.48, 140.59, 131.90, 128.18, 127.58, 127.05, 126.18, 42.27, 30.52; MS (ESI) m/z ESI$^+$ 302.25 $[M+H]^+$.

6-(3-oxobenzo[d][1,2]selenazol-2(3H)-yl)hexyl carbamimidothioate hydrobromide (**3b**)

Yellow solid (227mg, yield 52%), mp 196-198 °C; ^{1}H NMR (500 MHz, DMSO-d_6) δ 9.03 (d, *J* = 15.6 Hz, 4H), 8.16 (d, *J* = 8.0 Hz, 1H), 7.78 (dd, *J* = 7.8, 0.6 Hz, 1H), 7.60 – 7.55 (m, 1H), 7.42 – 7.37 (m, 1H), 3.70 (t, *J* = 7.0 Hz, 2H), 3.12 (t, *J* = 7.3 Hz, 2H), 1.60 (m, *J* = 15.2, 7.4 Hz, 4H), 1.43 – 1.39 (m, 2H), 1.38 – 1.31 (m, 2H); ^{13}C NMR (126 MHz, DMSO-d_6) δ 170.38, 166.65, 139.69, 131.64, 128.73, 127.62, 126.62, 126.12, 43.42, 30.48, 30.22, 28.69, 27.88, 25.93; MS (ESI) m/z ESI$^+$ 358.35 $[M+H]^+$.

2-(5-methoxy-3-oxobenzo[d][1,2]selenazol-2(3H)-yl)ethyl carbamimidothioate hydrobromide (**3c**)

Yellow solid (164mg, yield 40%), mp 175-177 °C; ^{1}H NMR (500 MHz, DMSO-d_6) δ 9.14 (d, J = 98.1 Hz, 4H), 8.24 (d, J = 8.8 Hz, 1H), 7.28 (d, J = 2.7 Hz, 1H), 7.21 (dd, J = 8.8, 2.7 Hz, 1H), 3.95 (t, J = 6.7 Hz, 2H), 3.80 (s, 3H), 3.43 (t, J = 6.7 Hz, 2H). ^{13}C NMR (126 MHz, DMSO-d_6) δ 170.12, 167.16, 158.59, 131.36, 129.21, 128.32, 120.70, 109.87, 55.89, 42.41, 30.52; MS (ESI) m/z ESI$^+$ 332.27 $[M+H]^+$.

6-(5-methoxy-3-oxobenzo[d][1,2]selenazol-2(3H)-yl)hexyl carbamimidothioate hydrobromide (**3d**)

Yellow solid (280mg, yield 60%), mp 178-180 °C; ^{1}H NMR (500 MHz, DMSO-d_6) δ 9.09 (s, 4H), 8.12 (d, J = 8.8 Hz, 1H), 7.27 (d, J = 2.7 Hz, 1H), 7.20 (dd, J = 8.8, 2.7 Hz, 1H), 3.81 (s, 3H), 3.67 (t, J = 7.0 Hz, 2H), 3.12 (t, J = 7.3 Hz, 2H), 1.64 – 1.55 (m, 4H), 1.42 – 1.36 (m, H), 1.35 – 1.27 (m, 2H). ^{13}C NMR (126 MHz, DMSO-d_6) δ 170.17, 166.32, 158.56, 130.48, 129.81, 127.79, 120.44, 109.97, 55.86, 43.55, 30.46, 30.25, 28.71, 27.90, 25.93; MS (ESI) m/z ESI$^+$ 388.35 $[M+H]^+$.

1,1,3,3-tetramethyl-2-(6-(3-oxobenzo[d][1,2]selenazol-2(3H)-yl)hexyl)isothiouronium hydrobromide (**4**)

Yellow solid (270mg, yield 55%), mp 171-173 °C; ^{1}H NMR (500 MHz, DMSO-d_6) δ 8.47 (d, J = 8.0 Hz, 1H), 7.74 (d, J = 7.6 Hz, 1H), 7.52 (t, J = 7.6 Hz, 1H), 7.36 (t, J = 7.4 Hz, 1H), 3.67 (t, J = 6.9 Hz, 2H), 3.21 (s, 12H), 3.00 (t, J = 7.2 Hz, 2H), 1.64 – 1.53 (m, 4H), 1.41 – 1.28 (m, 4H); ^{13}C NMR (126 MHz, DMSO-d_6) δ 174.49, 166.52, 140.21, 131.07, 129.33, 127.45, 127.27, 125.85, 43.92, 43.10, 34.20, 30.25, 29.57, 27.92, 25.96; MS (ESI) m/z ESI$^+$ 414.46 $[M+H]^+$.

N-(6-bromohexyl)-N-methyl-4-nitroaniline (**5**)

Yellow solid (440mg, yield 70%), mp 56-58 °C; ^{1}H NMR (500 MHz, $CDCl_3$) δ 8.11 (d, J = 8.9 Hz, 2H), 6.58 (d, J = 8.9 Hz, 2H), 3.45 – 3.39 (m, 4H), 3.07 (s, 3H), 1.91 – 1.84 (m, 2H), 1.68 – 1.61 (m, 2H), 1.54 – 1.47 (m, 2H), 1.38 (dd, J = 15.2, 7.9 Hz, 2H); ^{13}C NMR (126 MHz, $CDCl_3$) δ 153.34, 136.74, 126.27, 110.07, 52.51, 38.77, 33.62, 32.56, 27.91, 26.76, 26.15; MS (ESI) m/z ESI$^+$ 316.21 $[M+H]^+$.

2-(4-((6-bromohexyl) (methyl) amino) phenyl) benzo[d][1,2]selenazol-3(2H)-one (**6a**)

Yellow solid (163mg, yield 35%), mp 108-110 °C; ^{1}H NMR (500 MHz, $CDCl_3$) δ 8.10 (d, J = 7.7 Hz, 1H), 7.62 (dt, J = 15.8, 7.8 Hz, 2H), 7.44 (t, J = 7.7 Hz, 1H), 7.38 (d, J = 8.9 Hz, 2H), 6.68 (d, J = 8.7 Hz, 2H), 3.41 (t, J = 6.7 Hz, 2H), 3.33 (t, J = 7.4 Hz, 2H), 2.95 (s, 3H), 1.91 – 1.76 (m, 2H), 1.60 (dt, J = 15.1, 7.6 Hz, 2H), 1.48 (dt, J = 15.1, 7.5 Hz, 2H), 1.35 (dt, J = 15.1, 7.5 Hz, 2H); ^{13}C NMR (126 MHz, $CDCl_3$) δ 165.97, 148.31, 138.07, 133.74, 132.08, 130.57, 129.23, 127.38,

126.28, 123.76, 112.04, 52.64, 38.56, 33.84, 32.70, 28.03, 26.76, 26.29; MS (ESI) m/z ESI$^+$ 467.23 $[M+H]^+$.

2-(4-((6-bromohexyl) (methyl) amino) phenyl)-5-methoxybenzo[d][1,2]selenazol-3(2H)-one (**6b**)

Yellow solid (184mg, yield 37%), mp 143-145 °C;^{1}H NMR (500 MHz, $CDCl_3$) δ 7.60 (s, 1H), 7.51 (d, *J* = 8.5 Hz, 1H), 7.39 (d, *J* = 7.9 Hz, 2H), 7.26 (s, 1H), 6.69 (d, *J* = 8.1 Hz, 2H), 3.91 (s, 3H), 3.43 (t, *J* = 6.6 Hz, 2H), 3.35 (t, *J* = 7.2 Hz, 2H), 2.97 (s, 3H), 1.92 – 1.85 (m, 2H), 1.65 – 1.60 (m, 2H), 1.53 – 1.47 (m, 2H), 1.40 – 1.35 (m, 2H); ^{13}C NMR (126 MHz, $CDCl_3$) δ 165.85, 159.01, 148.35, 145.67, 128.88, 128.48, 127.31, 124.42, 122.08, 112.02, 111.02, 55.77, 52.64, 38.54, 33.79, 32.71, 28.04, 26.59, 26.31; MS (ESI) m/z ESI$^+$ 497.31 $[M+H]^+$.

6-(methyl(4-(3-oxobenzo[d][1,2]selenazol-2(3H)-yl)phenyl)amino)hexyl carbamimidothioate hydrobromide (**7a**)

Yellow solid (143mg, yield 53%), mp 138-140 °C; ^{1}H NMR (500 MHz, DMSO-d_6) δ 9.08 (s, 4H), 8.24 (d, *J* = 8.0 Hz, 1H), 7.83 (d, *J* = 7.7 Hz, 1H), 7.62 (t, *J* = 7.6 Hz, 1H), 7.44 (t, *J* = 7.4 Hz, 1H), 7.32 (d, *J* = 7.9 Hz, 2H), 6.71 (d, *J* = 8.1 Hz, 2H), 3.34 (t, *J* = 6.6 Hz, 2H), 3.14 (t, *J* = 7.1 Hz, 2H), 2.90 (s, 3H), 1.64 – 1.58 (m, 2H), 1.54 – 1.48 (m, 2H), 1.40 (m, 2H), 1.34 – 1.30 (m, 2H); ^{13}C NMR (126 MHz, DMSO-d_6) δ 170.40, 165.20, 147.71, 139.88, 131.87, 130.10, 129.35, 128.37, 127.96, 126.76, 126.32, 112.34, 52.16, 38.59, 30.47, 28.83, 28.14, 26.29, 26.18; MS (ESI) m/z ESI$^+$ 451.48 $[M+H]^+$.

6-((4-(5-methoxy-3-oxobenzo[d][1,2]selenazol-2(3H)-yl)phenyl)(methyl)amino)hexyl carbamimidothioate hydrobromide (**7b**)

Yellow solid (128mg, yield 45%), mp 145-147 °C; ^{1}H NMR (500 MHz, CD_3OD) δ 7.80 (d, *J* = 8.8 Hz, 1H), 7.45 (d, *J* = 8.9 Hz, 2H), 7.30 (d, *J* = 2.5 Hz, 1H), 6.93 (dd, *J* = 8.9, 2.6 Hz, 1H), 6.76 (d, *J* = 8.9 Hz, 2H), 3.83 (s, 3H), 3.37 (t, 2H), 3.15 (t, *J* = 7.2 Hz, 2H), 2.94 (s, 3H), 1.75 – 1.70 (m, 2H), 1.65-1.59 (m, *J* = 7.0 Hz, 2H), 1.54 – 1.47 (m, 2H), 1.44 – 1.39 (m, 2H); ^{13}C NMR (126 MHz, CD_3OD) δ 171.64, 167.37, 159.00, 147.19, 136.44, 133.31, 130.98, 128.44, 122.75, 117.10, 112.91 , 112.27 , 54.73, 52.32 37.47, 30.40, 29.33, 28.24, 26.10, 26.00; MS (ESI) m/z ESI$^+$ 481.52 $[M+H]^+$.

1,1,3,3-tetramethyl-2-(6-(methyl(4-(3-oxobenzo[d][1,2]selenazol-2(3H)-yl)phenyl)amino)hexyl)isothiouronium hydrobromide (**8a**)

Yellow solid (134mg, yield 45%), mp 125-127 °C; ^{1}H NMR (500 MHz, DMSO-d_6) δ 8.34 (d, *J* = 7.7 Hz, 1H), 7.82 (d, *J* = 7.7 Hz, 1H), 7.60 (t, *J* = 7.6 Hz, 1H), 7.43 (t, *J* = 7.4 Hz, 1H), 7.33 (d,

J = 8.4 Hz, 2H), 6.72 (d, J = 8.3 Hz, 2H), 3.33 (t, J = 7.2 Hz, 2H), 3.22 (s, 12H), 3.02 (t, J = 7.2 Hz, 2H), 2.90 (s, 3H), 1.62 – 1.56 (m, 2H), 1.55 – 1.49 (m, 2H), 1.43 – 1.37 (m, 2H), 1.34 – 1.30 (m, 2H); ^{13}C NMR (126 MHz, DMSO-d$_6$) δ 174.62, 165.10, 147.62, 139.92, 131.69, 129.62, 128.61, 127.85, 127.02, 126.65, 126.24, 112.36, 52.11, 43.90, 38.59, 34.21, 29.67, 28.29, 26.35, 26.26; MS (ESI) m/z ESI$^+$ 519.60 [M+H]$^+$.

2-(6-((4-(5-methoxy-3-oxobenzo[d][1,2]selenazol-2(3H)-yl)phenyl)(methyl)amino)hexyl)-1,1,3,3-tetramethylisothiouronium hydrobromide (**8b**)

Yellow solid (173mg, yield 55%), mp 130-132 °C; ^{1}H NMR (500 MHz, CD$_3$OD) δ 7.84 (d, J = 8.8 Hz, 1H), 7.51 (d, J = 2.6 Hz, 1H), 7.34 – 7.29 (m, 3H), 6.79 (d, J = 8.9 Hz, 2H), 3.90 (s, 3H), 3.42 (t, J = 7.2 Hz, 2H), 3.30 (s, 12H), 3.06 (t, J = 7.3 Hz, 2H), 2.98 (s, 3H), 1.74 – 1.68 (m, 2H), 1.66 – 1.61 (m, 2H), 1.55 – 1.47 (m, 2H), 1.44 –1.39 (m, 2H); ^{13}C NMR (126 MHz, CD$_3$OD) δ 176.00, 166.53, 159.18, 148.64, 130.71, 129.43, 128.63, 126.80, 125.65, 121.09, 111.93, 109.96, 54.71, 51.86, 42.81, 37.38, 34.04, 29.30, 28.10, 26.17, 26.07; MS (ESI) m/z ESI$^+$ 549.63 [M+H]$^+$.

(E)-1,2-diBoc-3-(2-(3-oxobenzo[d][1,2]selenazol-2(3H)-yl)ethyl)guanidine (**10a**)

Yellow solid (325mg, yield 67%), mp 157-159 °C; ^{1}H NMR (500 MHz, CDCl$_3$) δ 11.45 (s, 1H), 8.55 (t, J = 4.7 Hz, 1H), 8.04 (d, J = 7.7 Hz, 1H), 7.62 (d, J = 7.9 Hz, 1H), 7.60 – 7.56 (m, 1H), 7.44 – 7.39 (m, 1H), 4.05 (t, J = 6.0 Hz, 2H), 3.74 (q, J = 5.9 Hz, 2H), 1.49 (s, 9H), 1.45 (s, 9H); ^{13}C NMR (126 MHz, CDCl$_3$) δ 167.49, 163.37, 156.43, 152.97, 138.18, 132.04, 128.83, 126.82, 126.18, 123.97, 83.24, 79.38, 43.81, 40.71, 28.27, 28.00; MS (ESI) m/z ESI$^+$ 486.44 [M+H]$^+$.

(E)-1,2-diBoc-3-(6-(3-oxobenzo[d][1,2]selenazol-2(3H)-yl)hexyl)guanidine (**10b**)

Yellow solid (379mg, yield 70%), mp 152-154 °C; ^{1}H NMR (500 MHz, CDCl$_3$) δ 11.47 (s, 1H), 8.27 (s, 1H), 8.00 (d, J = 7.8 Hz, 1H), 7.63 (d, J = 8.0 Hz, 1H), 7.56 – 7.51 (m, 1H), 7.37 (t, J = 7.5 Hz, 1H), 3.81 (t, J = 7.2 Hz, 2H), 3.36 (dd, J = 12.6, 7.0 Hz, 2H), 1.73 – 1.66 (m, 2H), 1.56 – 1.50 (m, 2H), 1.45 (d, J = 6.3 Hz, 18H), 1.39 – 1.34 (m, 4H); ^{13}C NMR (126 MHz, CDCl$_3$) δ 167.11, 163.57, 156.07, 153.25, 137.72, 131.80, 128.71, 127.54, 126.11, 124.04, 82.98, 79.15, 44.65, 40.74, 30.31, 28.83, 28.28, 28.04, 26.46, 26.23; MS (ESI) m/z ESI$^+$ 542.55 [M+H]$^+$.

(E)-1-(2-(5-methoxy-3-oxobenzo[d][1,2]selenazol-2(3H)-yl)ethyl)-2,3-diBocguanidine (**10c**)

Yellow solid (310mg, yield 60%), mp 153-155 °C; ^{1}H NMR (500 MHz, CDCl$_3$) δ 11.44 (s, 1H), 8.53 (t, J = 5.3 Hz, 1H), 7.50 (d, J = 2.6 Hz, 1H), 7.48 (d, J = 8.7 Hz, 1H), 7.19 (dd, J = 8.7, 2.6 Hz, 1H), 4.01 (t, J = 6.0 Hz, 2H), 3.84 (s, 3H), 3.72 (q, J = 5.9 Hz, 2H), 1.47 (s, 9H), 1.43 (s,

9H); ^{13}C NMR (126 MHz, $CDCl_3$) δ 167.37, 163.35, 158.85, 156.40, 152.94, 129.03, 127.82, 124.72, 121.98, 110.52, 83.18, 79.34, 55.67, 43.98, 40.69, 28.25, 27.98; MS (ESI) m/z ESI$^+$ 516.47 $[M+H]^+$.

1-(2-(3-oxobenzo[d][1,2]selenazol-2(3H)-yl)ethyl)guanidine hydrochloride (**11a**)

Yellow viscous liquid (148mg, yield 93%), mp 153-155 °C; ^{1}H NMR (500 MHz, DMSO-d_6) δ 8.15 (d, J = 8.1 Hz, 1H), 7.87 (t, J = 5.6 Hz, 1H), 7.82 (d, J = 7.1 Hz, 1H), 7.63 – 7.58 (m, 1H), 7.44 – 7.39 (m, 1H), 3.85 (t, J = 6.0 Hz, 2H), 3.41 (q, J = 5.9 Hz, 2H); ^{13}C NMR (126 MHz, DMSO-d_6) δ 167.37, 157.58, 140.46, 131.88, 128.18, 127.65, 126.60, 126.11, 42.65, 41.25; MS (ESI) m/z ESI$^+$ 285.20 $[M+H]^+$.

1-(6-(3-oxobenzo[d][1,2]selenazol-2(3H)-yl)hexyl)guanidine hydrochloride (**11b**)

Yellow viscous liquid (169mg, yield 90%), mp 160-162 °C; ^{1}H NMR (500 MHz, DMSO-d_6) δ 8.20 (d, J = 8.0 Hz, 1H), 7.76 (d, J = 7.6 Hz, 1H), 7.68 (t, J = 5.3 Hz, 1H), 7.58 – 7.52 (m, 1H), 7.38 (t, J = 7.4 Hz, 1H), 3.67 (d, J = 7.1 Hz, 2H), 3.06 (dd, J = 12.8, 6.7 Hz, 2H), 1.63 – 1.59 (m, 2H), 1.45 – 1.41 (m, 2H), 1.34 – 1.28 (m, 4H); ^{13}C NMR (126 MHz, DMSO-d_6) δ 166.61, 157.36, 139.89, 131.47, 128.88, 127.52, 126.76, 126.02, 43.39, 41.04, 30.33, 28.79, 26.15, 26.13; MS (ESI) m/z ESI$^+$ 341.31 $[M+H]^+$.

1-(2-(5-methoxy-3-oxobenzo[d][1,2]selenazol-2(3H)-yl)ethyl)guanidine hydrochloride (**11c**)

Yellow viscous liquid (166mg, yield 95%), mp 165-167 °C; ^{1}H NMR (500 MHz, DMSO-d_6) δ 8.15 (d, J = 8.1 Hz, 1H), 7.87 (t, J = 5.6 Hz, 1H), 7.82 (d, J = 7.1 Hz, 1H), 7.63 – 7.58 (m, 1H), 7.44 – 7.39 (m, 1H), 3.85 (t, J = 6.0 Hz, 2H), 3.41 (q, J = 5.9 Hz, 2H); ^{13}C NMR (126 MHz, DMSO-d_6) δ 167.37, 157.58, 140.46, 131.88, 128.18, 127.65, 126.60, 126.11, 42.65, 41.25; MS (ESI) m/z ESI$^+$ 315.23 $[M+H]^+$.

2-(4-(methylamino) phenyl) benzo[d][1,2]selenazol-3(2H)-one (**12a**)

Yellow solid (151mg, yield 50%); ^{1}H NMR (500 MHz, $CDCl_3$) δ 8.10 (d, J = 7.8 Hz, 1H), 7.66 – 7.59 (m, 2H), 7.45 (t, J = 7.0 Hz, 1H), 7.36 (d, J = 7.8 Hz, 2H), 6.63 (d, J = 7.7 Hz, 2H), 2.86 (s, 3H); ^{13}C NMR (126 MHz, $CDCl_3$) δ 165.96, 148.54, 138.00, 132.12, 129.28, 128.17, 127.54, 126.31, 123.70, 112.45, 30.73; MS (ESI) m/z ESI$^+$ 304.22 $[M+H]^+$.

benzyl methyl (4-(3-oxobenzo[d][1,2]selenazol-2(3H)-yl) phenyl) carbamate (**12b**)

Yellow solid (284mg, yield 65%), mp 147-149 °C; ^{1}H NMR (500 MHz, $CDCl_3$) δ 8.13 (d, J = 7.8 Hz, 1H), 7.67 (s, 2H), 7.62 (d, J = 7.2 Hz, 2H), 7.51 – 7.47 (m, 1H), 7.39 – 7.28 (m, 7H), 5.20 (s, 2H), 3.35 (s, 3H); ^{13}C NMR (126 MHz, $CDCl_3$) δ 165.74, 155.35, 137.53, 136.46, 132.62, 129.42,

128.49, 128.04, 127.90, 127.37, 126.61, 126.38, 125.78, 123.73, 67.50, 31.44; MS (ESI) m/z ESI$^+$ 438.36 [M+H]$^+$.

2-(4-(methyl(3-(piperidin-1-yl) propyl) amino) phenyl) benzo[d][1,2]selenazol-3(2H)-one (**12c**)

Yellow solid (85mg, yield 40%), mp 200-202 °C; ^{1}H NMR (500 MHz, DMSO-d_6) δ 8.30 (d, J = 8.0 Hz, 1H), 7.84 (d, J = 7.6 Hz, 1H), 7.61 (t, J = 7.4 Hz, 1H), 7.43 (t, J = 7.4 Hz, 1H), 7.35 (d, J = 8.8 Hz, 2H), 6.78 (d, J = 8.9 Hz, 2H), 3.40 (t, J = 7.0 Hz, 4H), 3.06 – 3.00 (m, 2H), 2.91 (s, 3H), 2.88 – 2.76 (m, 2H), 1.94 (m, 2H), 1.74 (s, 6H); ^{13}C NMR (126 MHz, DMSO-d$_6$) δ 165.17, 147.42, 139.96, 131.78, 129.51, 129.09, 127.90, 126.84, 126.65, 126.27, 112.76, 54.15, 52.51, 49.60, 38.49, 22.93, 21.81, 21.20; MS (ESI) m/z ESI$^+$ 429.44 [M+H]$^+$.

2-(2-(1H-imidazol-4-yl) ethyl) benzo[d][1,2]selenazol-3(2H)-one (**12d**)

White solid (184mg, yield 63%), mp 250-252 °C; ^{1}H NMR (500 MHz, DMSO-d$_6$) δ 11.86 (s, 1H), 8.00 (d, J = 8.0 Hz, 1H), 7.80 (d, J = 7.7 Hz, 1H), 7.60 – 7.55 (m, 2H), 7.40 (t, J = 7.4 Hz, 1H), 6.84 (s, 1H), 3.97 (t, J = 7.0 Hz, 2H), 2.84 (t, J = 6.9 Hz, 2H); ^{13}C NMR (126 MHz, DMSO-d$_6$) δ 166.68, 139.87, 135.27, 132.83, 131.80, 130.10, 128.31, 127.69, 126.21, 126.09, 43.73, 28.33; MS (ESI) m/z ESI$^+$ 293.20 [M+H]$^+$.

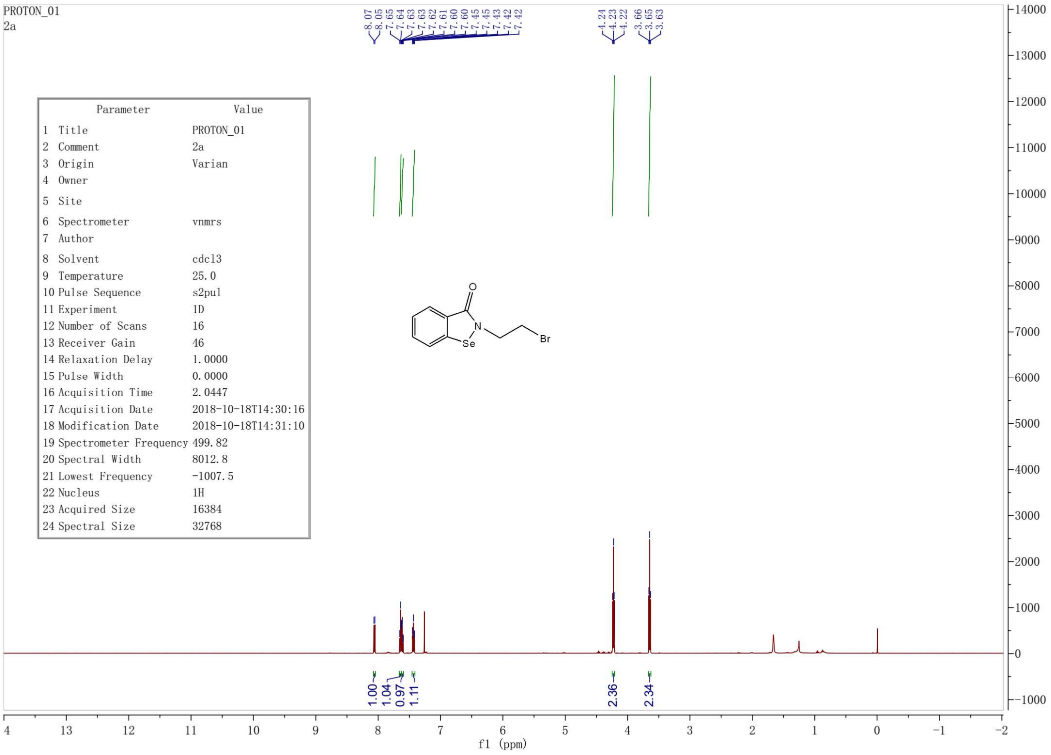

Fig. S3. ^{1}H NMR Spectrum of **2a** in $CDCl_3$ (500 MHz)

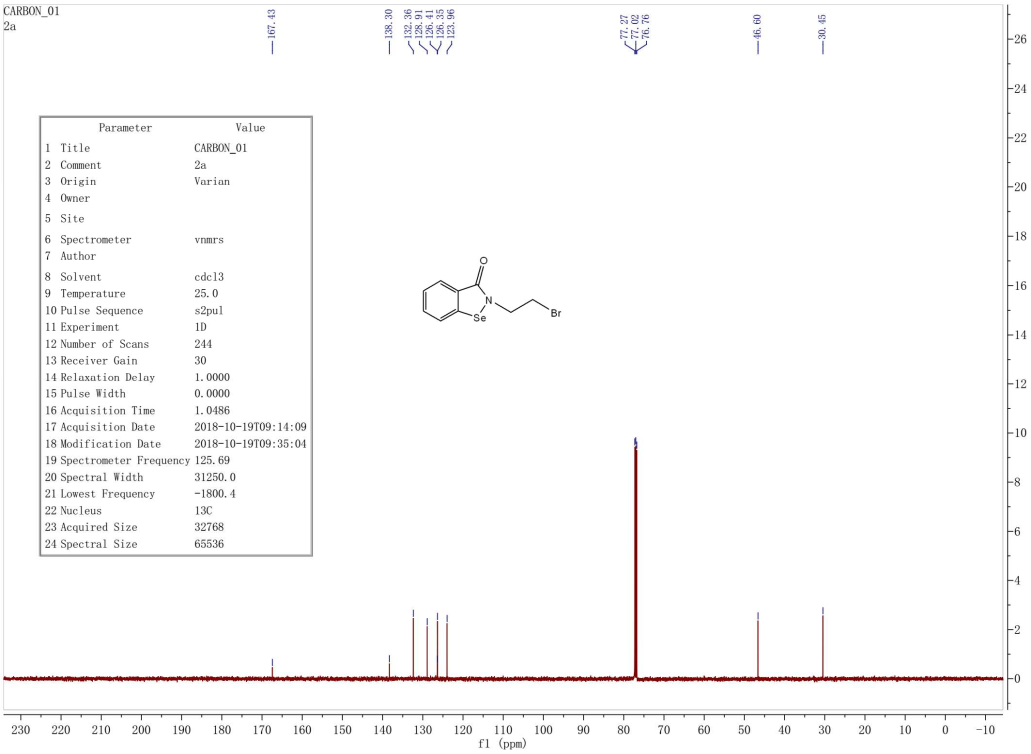

Fig. S4. ^{13}C NMR Spectrum of **2a** in $CDCl_3$ (126 MHz)

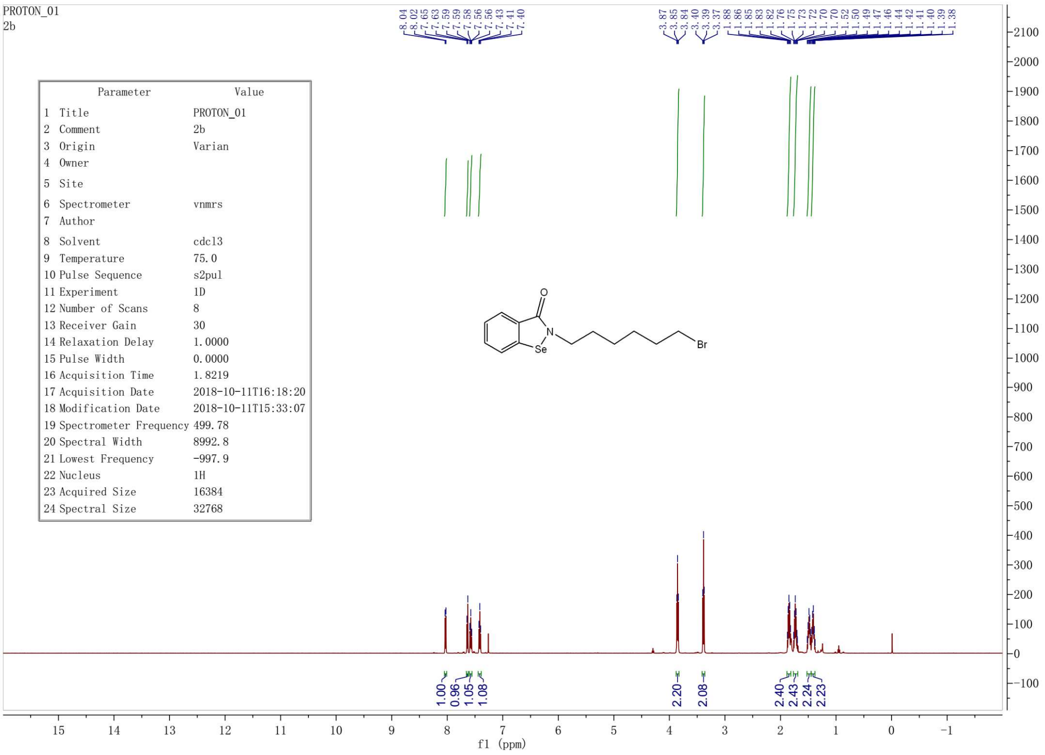

Fig. S5. ^{1}H NMR Spectrum of **2b** in $CDCl_3$ (500 MHz)

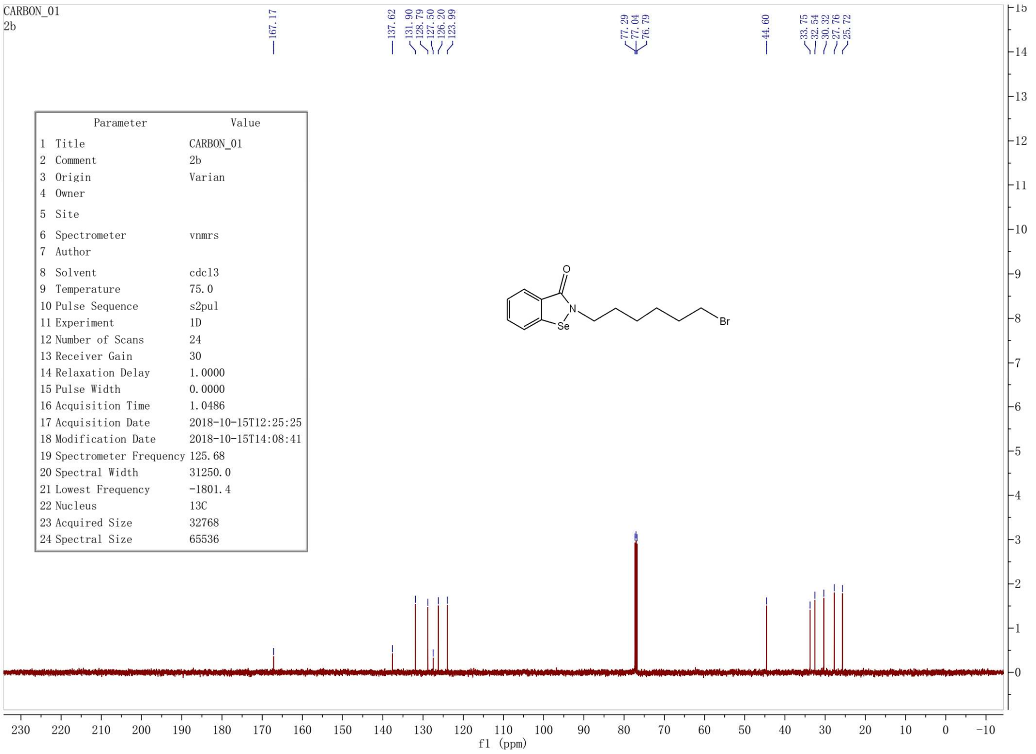

Fig. S6. ^{13}C NMR Spectrum of 2**b** in $CDCl_3$ (126 MHz)

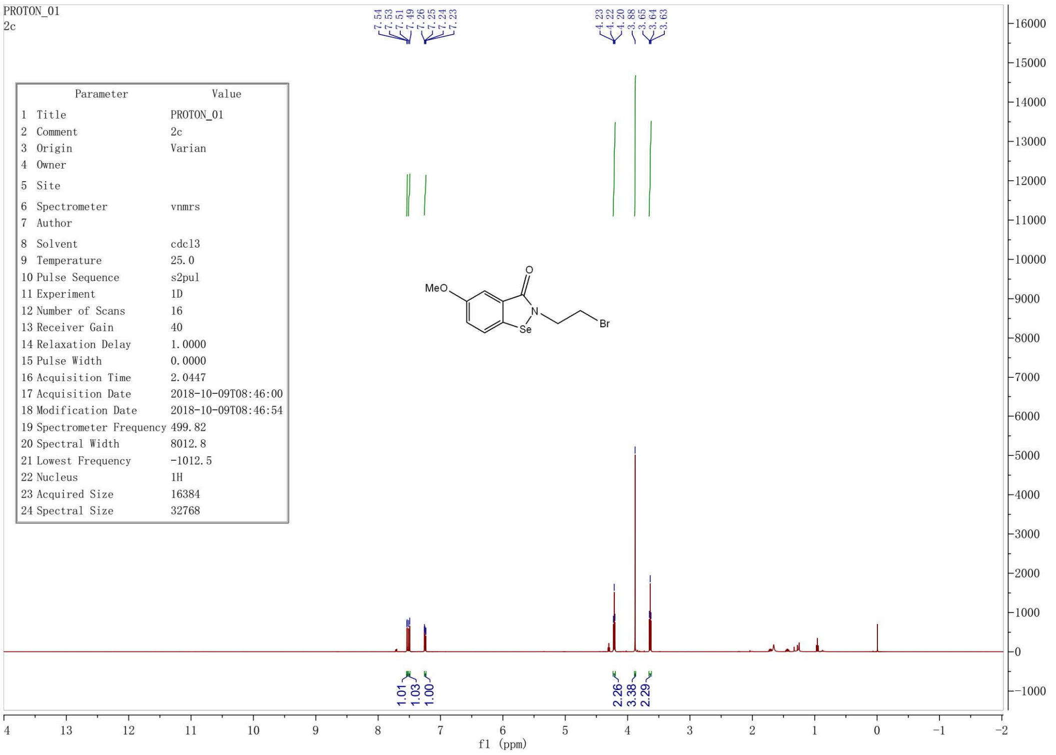

Fig. S7. ^{1}H NMR Spectrum of **2c** in $CDCl_3$ (500 MHz)

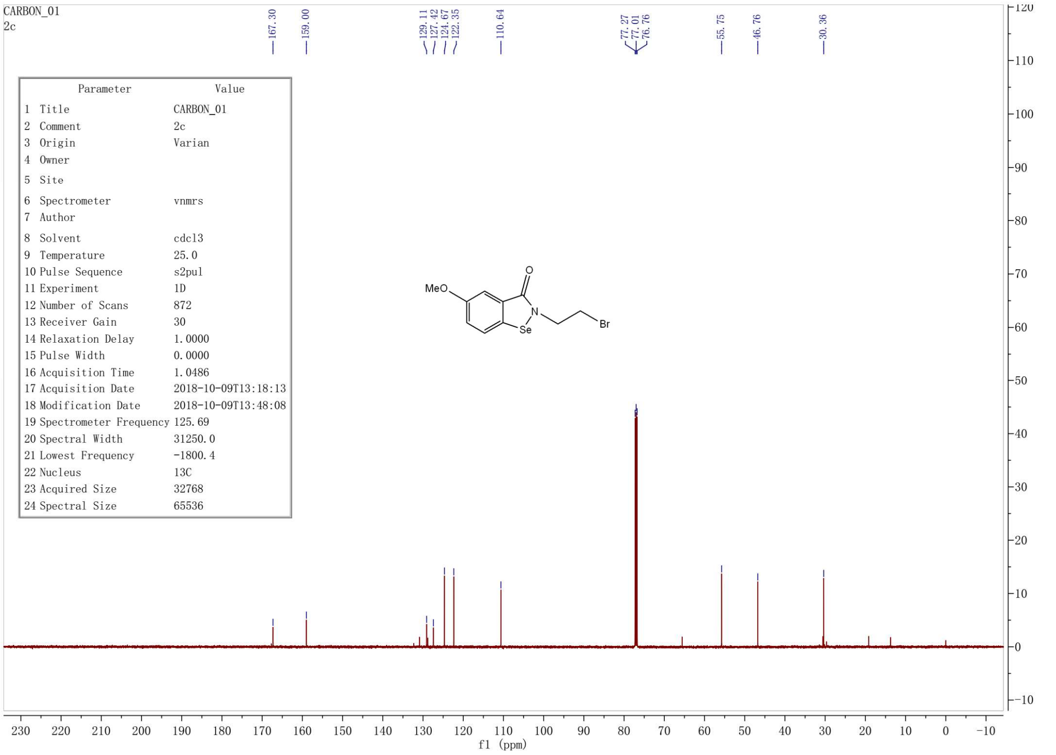

Fig. S8. ^{13}C NMR Spectrum of 2**c** in $CDCl_3$ (126 MHz)

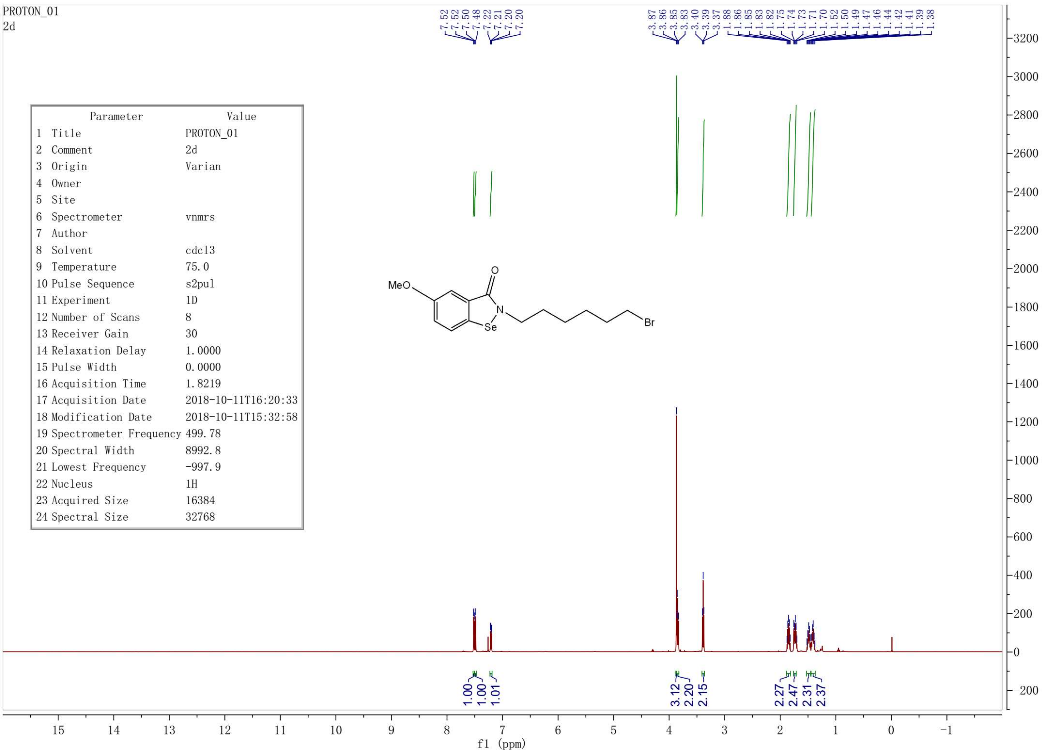

Fig. S9. ^{1}H NMR Spectrum of **2d** in $CDCl_3$ (500 MHz)

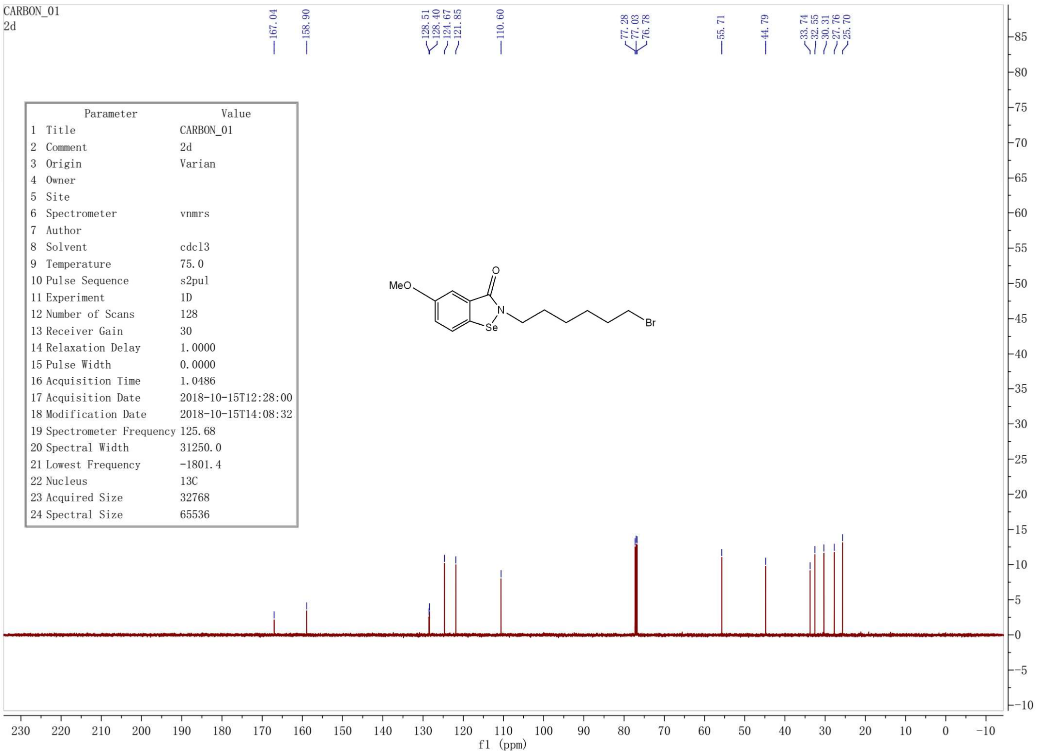

Fig. S10. ^{13}C NMR Spectrum of 2**d** in $CDCl_3$ (126 MHz)

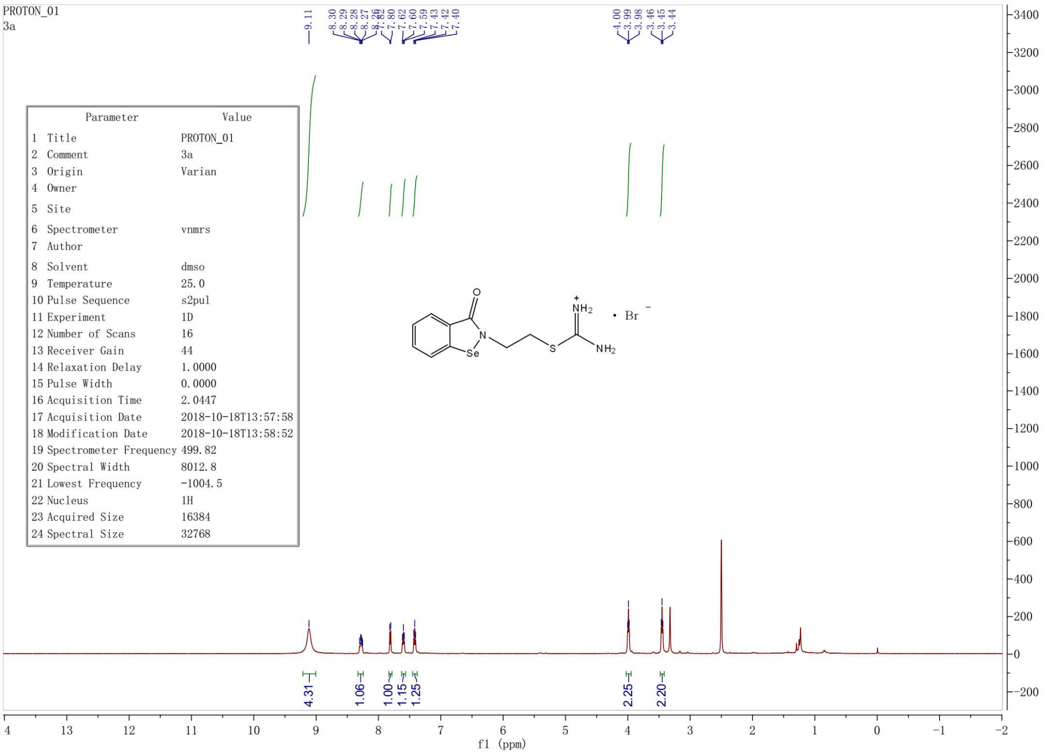

Fig. S11. ^{1}H NMR Spectrum of **3a** in DMSO-d_6 (500 MHz)

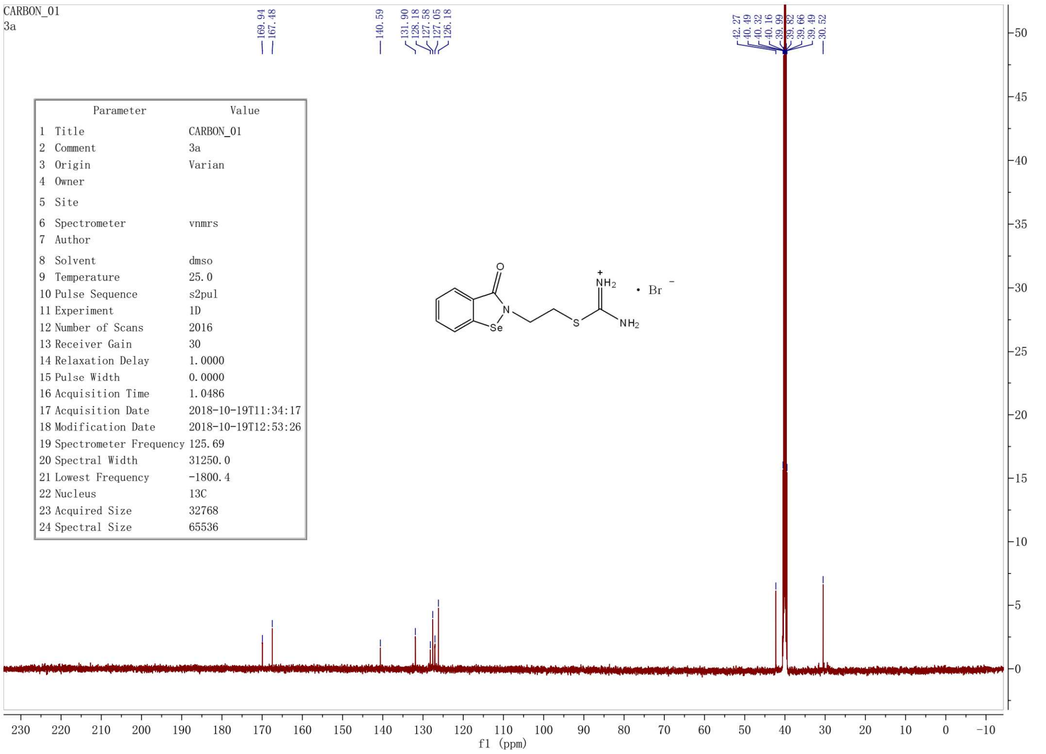

Fig. S12. ^{13}C NMR Spectrum of **3a** in DMSO-d_6 (126 MHz)

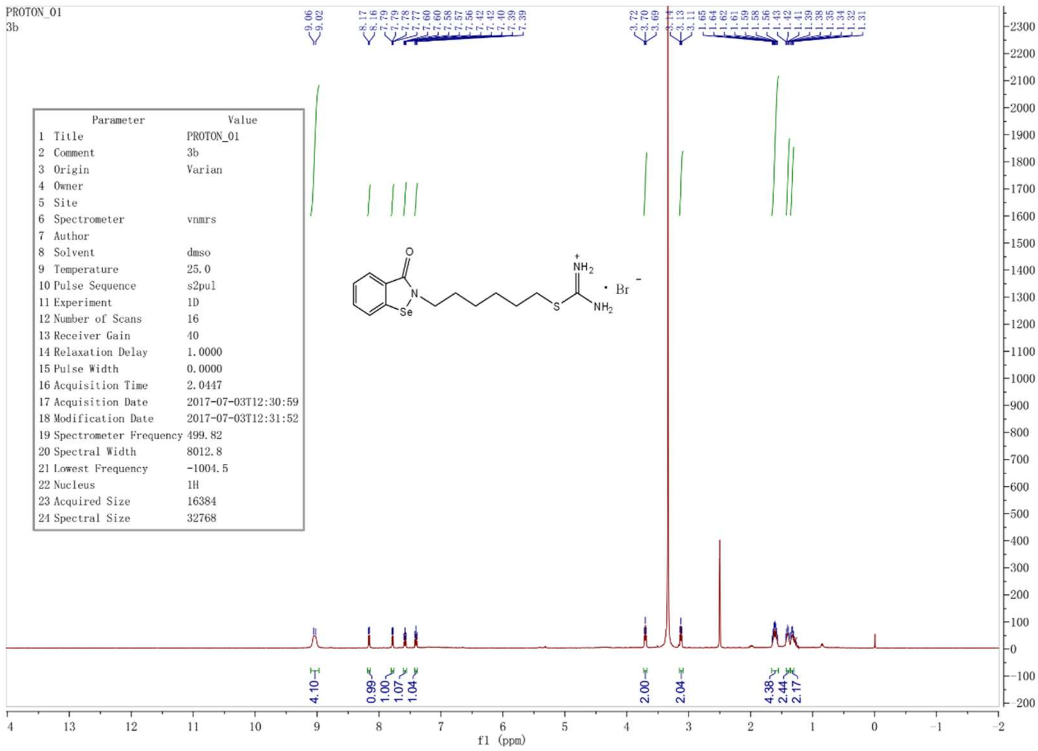

Fig. S13. ^{1}H NMR Spectrum of **3b** in DMSO-d_6 (500 MHz)

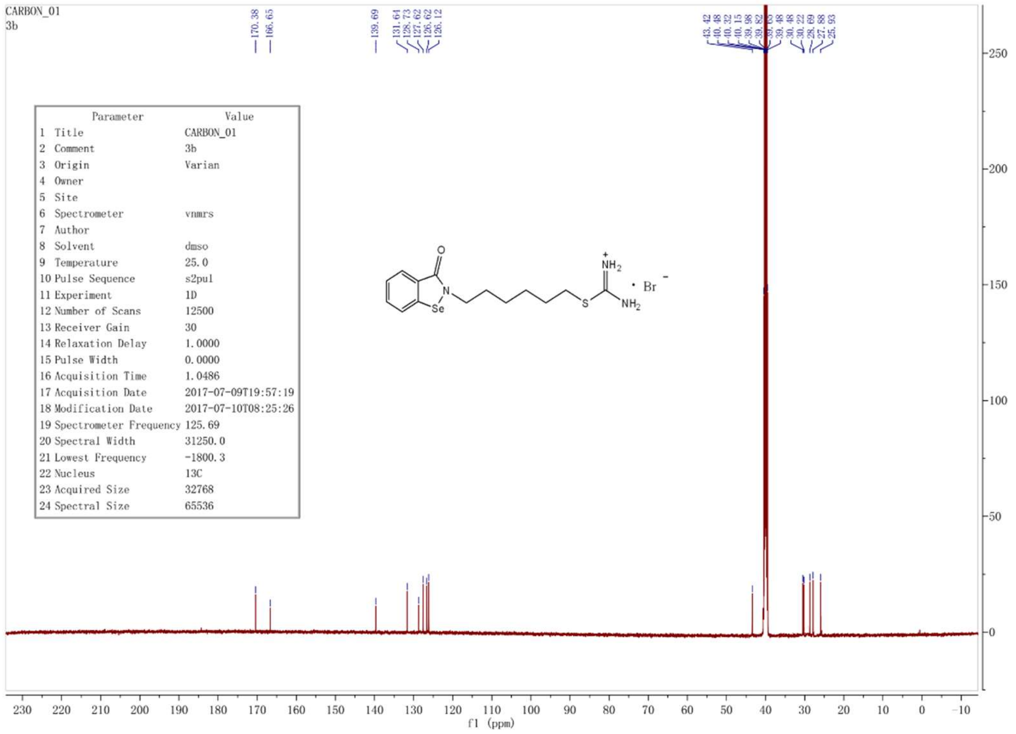

Fig. S14. ^{13}C NMR Spectrum of **3b** in DMSO-d_6 (126 MHz)

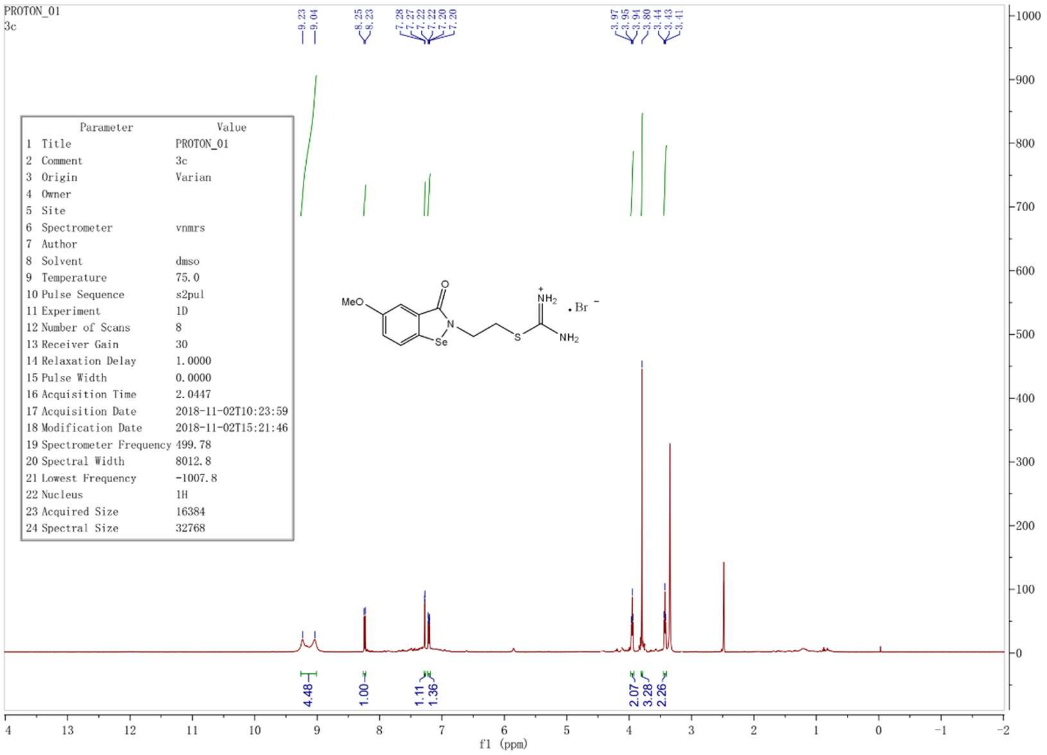

Fig. S15. ^{1}H NMR Spectrum of **3c** in DMSO-d_6 (500 MHz)

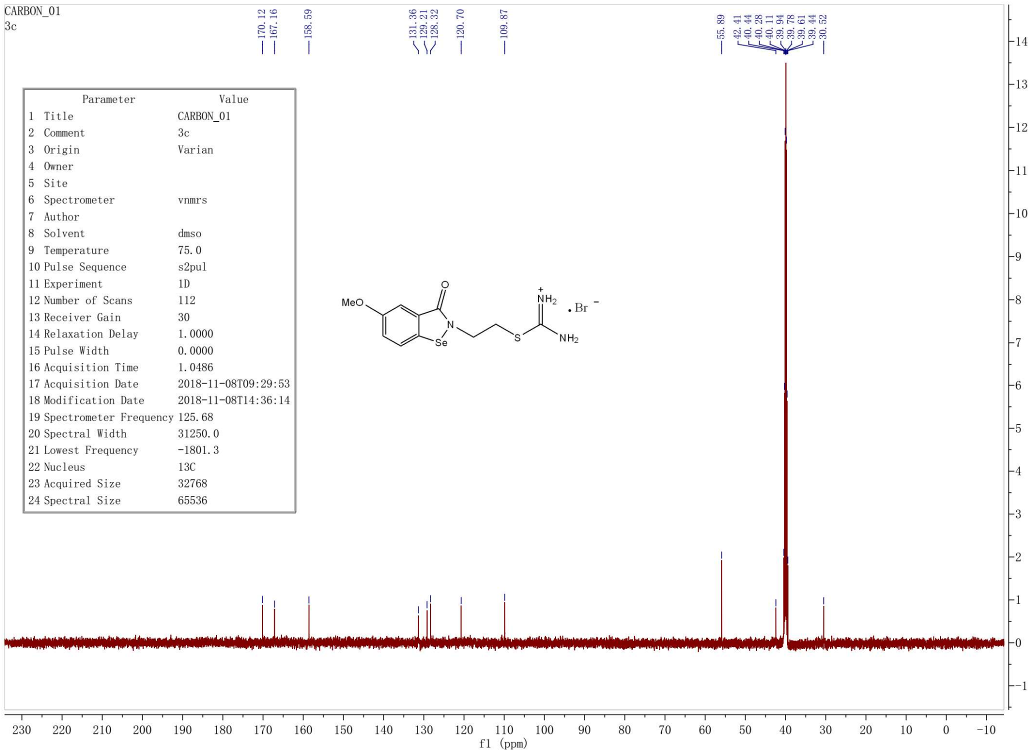

Fig. S16. ^{13}C NMR Spectrum of **3c** in DMSO-d_6 (126 MHz)

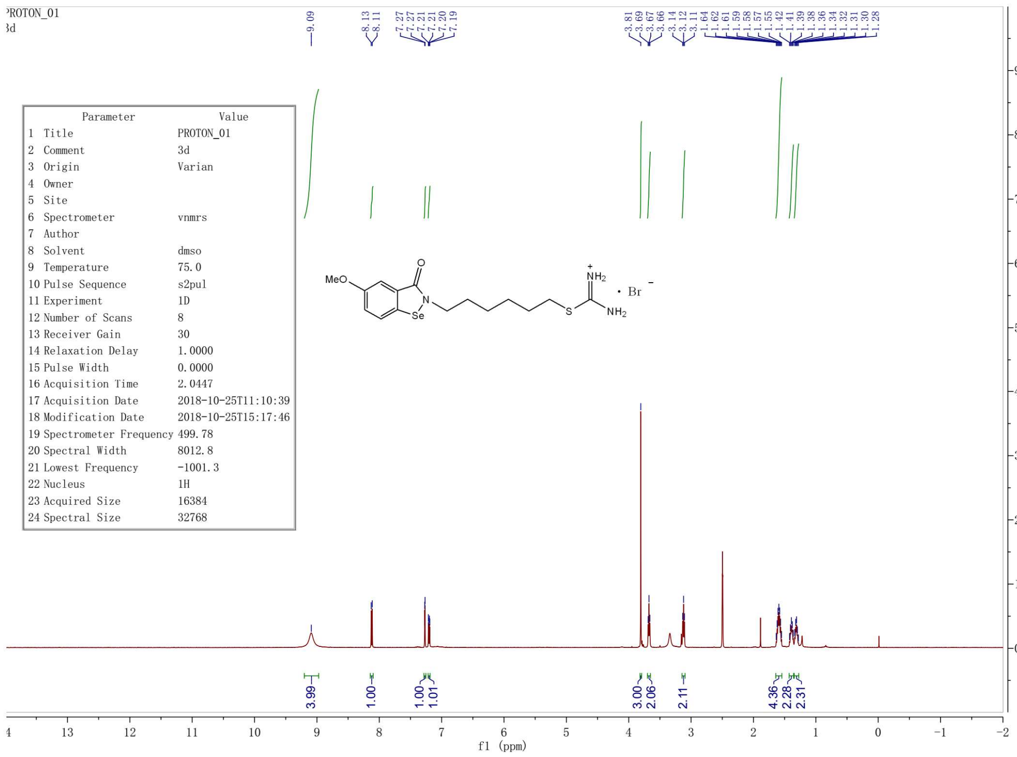

Fig. S17. ^{1}H NMR Spectrum of **3d** in DMSO-d_6 (500 MHz)

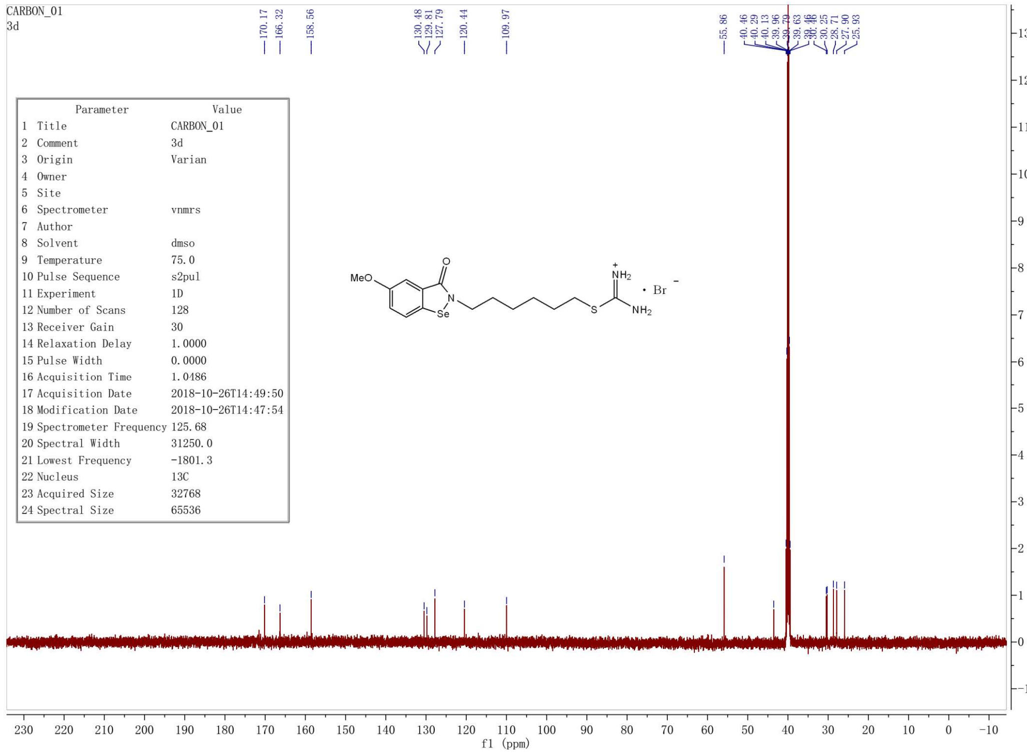

Fig. S18. ^{13}C NMR Spectrum of **3d** in DMSO-d_6 (126 MHz)

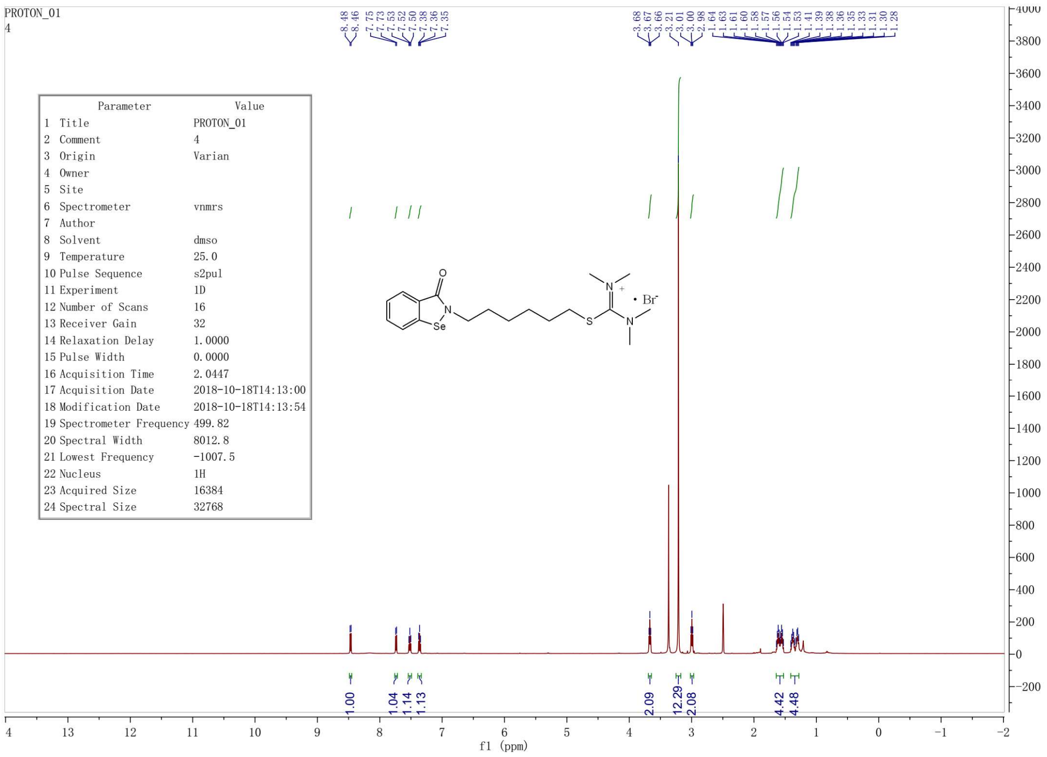

Fig. S19. ^{1}H NMR Spectrum of **4** in DMSO-d_6 (500 MHz)

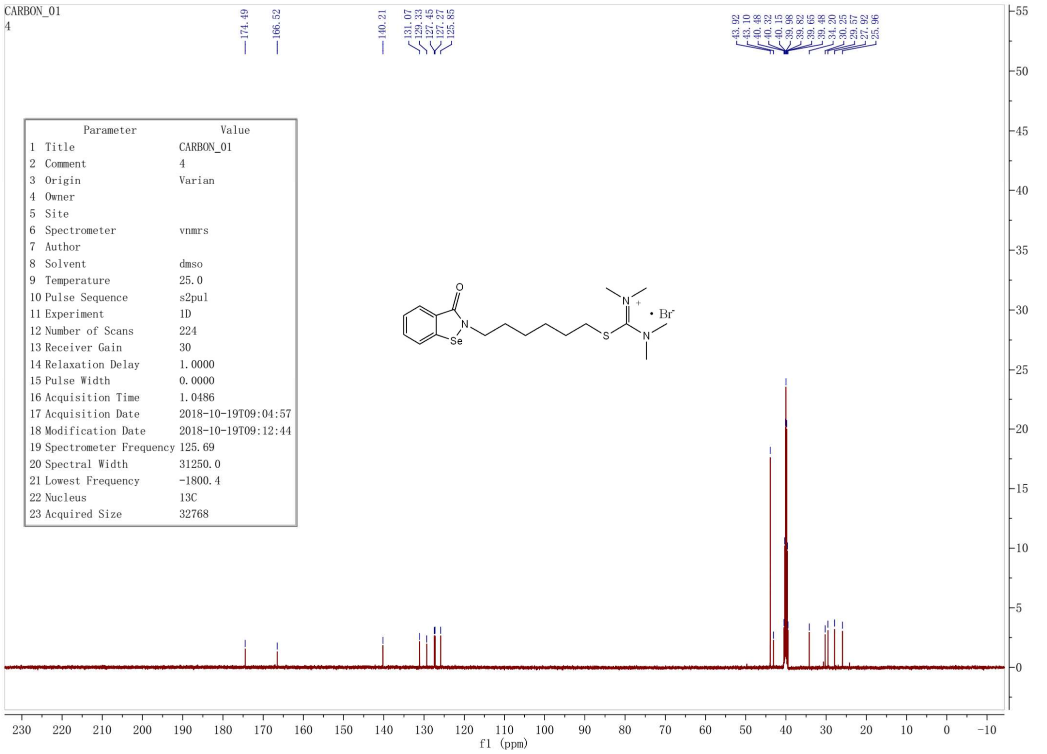

Fig. S20. ^{13}C NMR Spectrum of **4** in DMSO-d_6 (126 MHz)

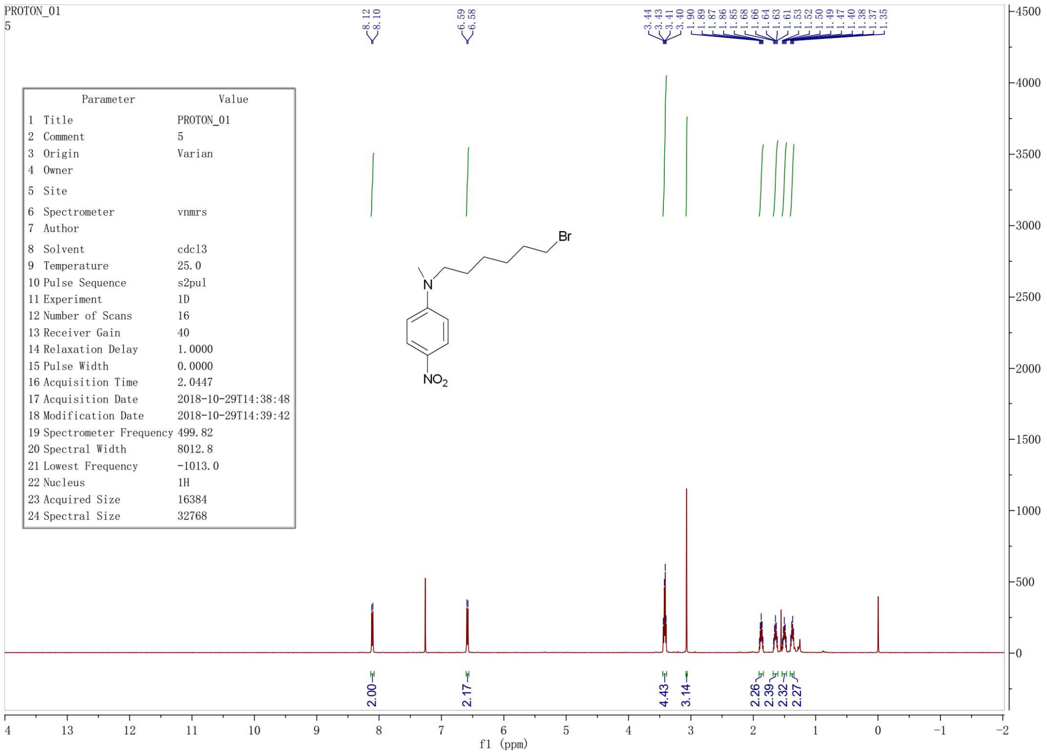

Fig. S21. ^{1}H NMR Spectrum of **5** in $CDCl_3$ (500 MHz)

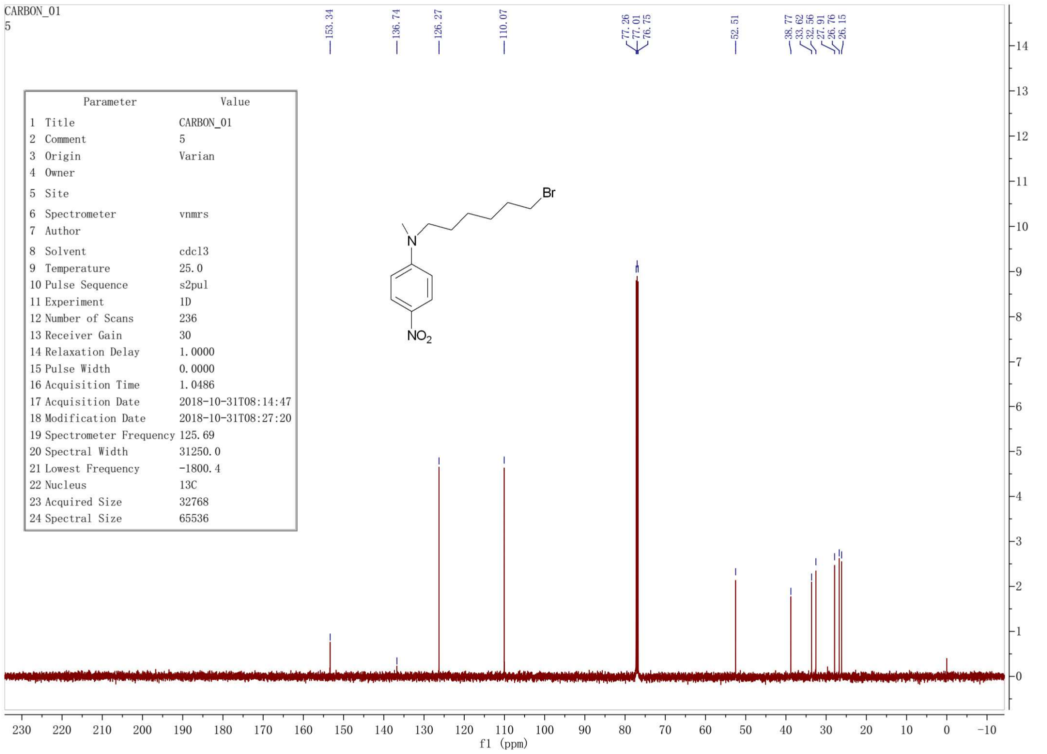

Fig. S22. ^{13}C NMR Spectrum of **5** in $CDCl_3$ (126 MHz)

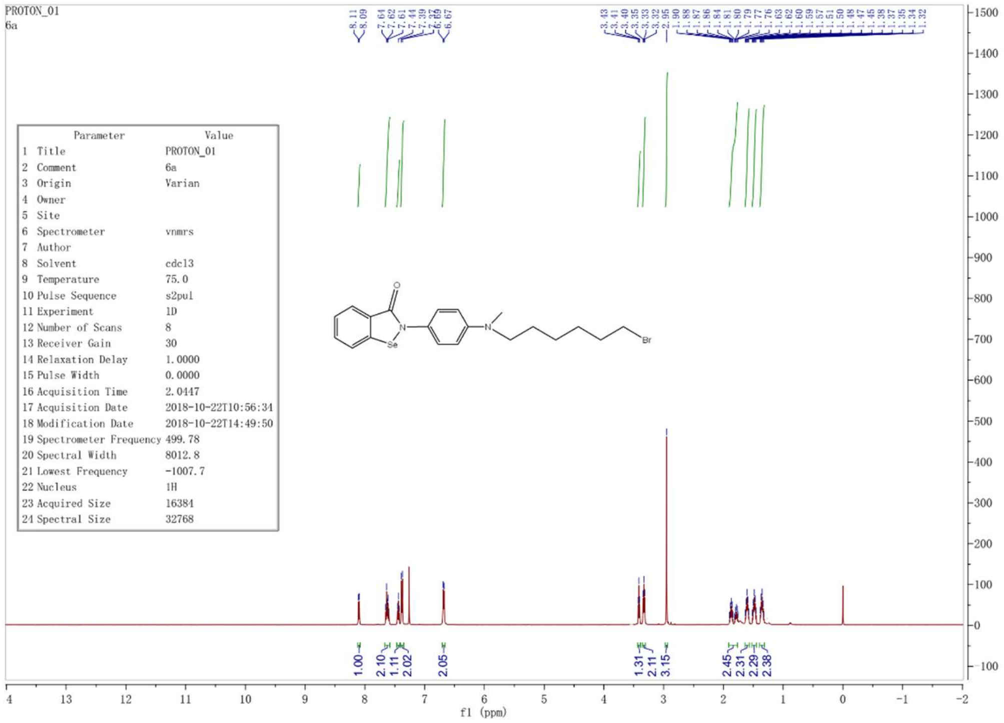

Fig. S23. ^{1}H NMR Spectrum of **6a** in $CDCl_3$ (500 MHz)

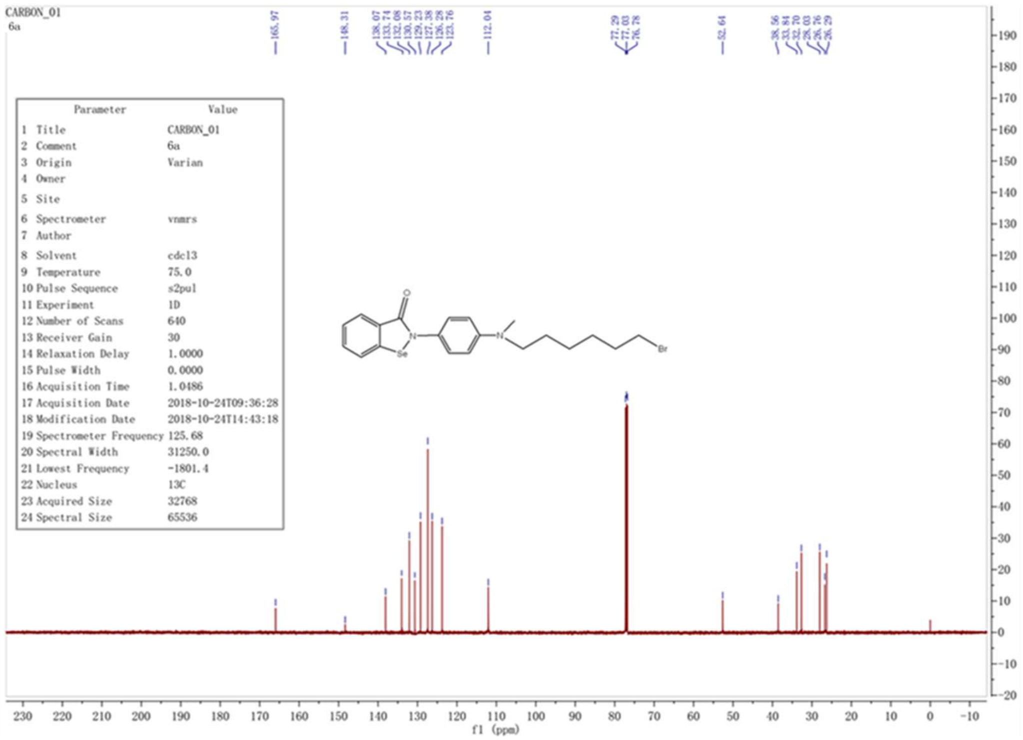

Fig. S24. ^{13}C NMR Spectrum of **6a** in $CDCl_3$ (126 MHz)

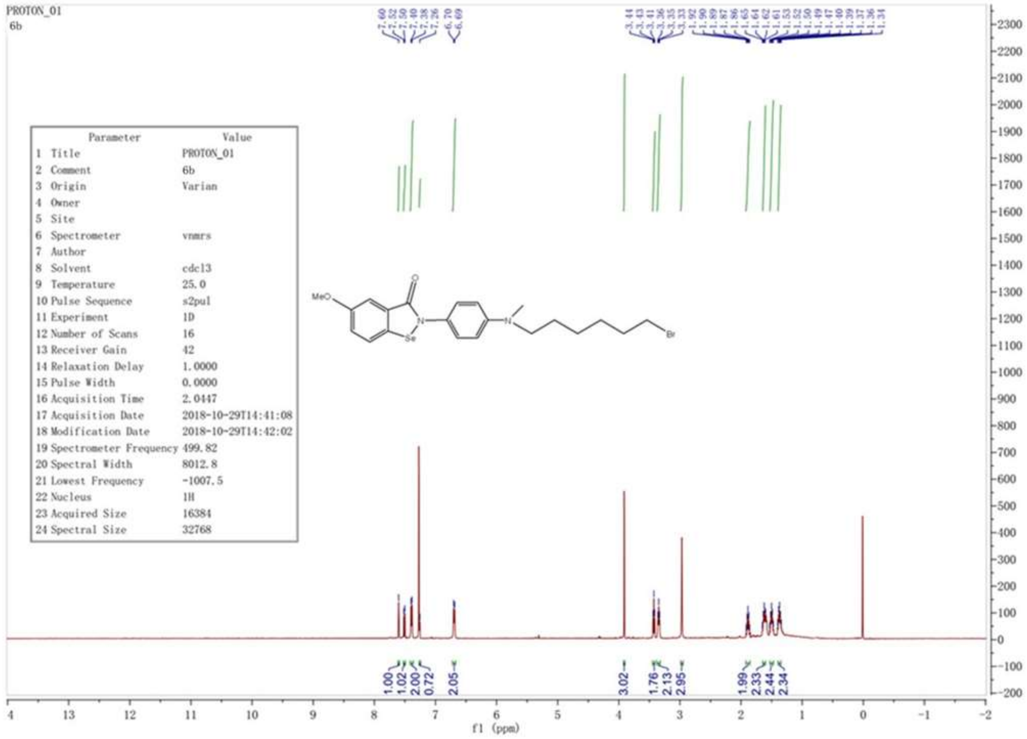

Fig. S25. ^{1}H NMR Spectrum of **6b** in $CDCl_3$ (500 MHz)

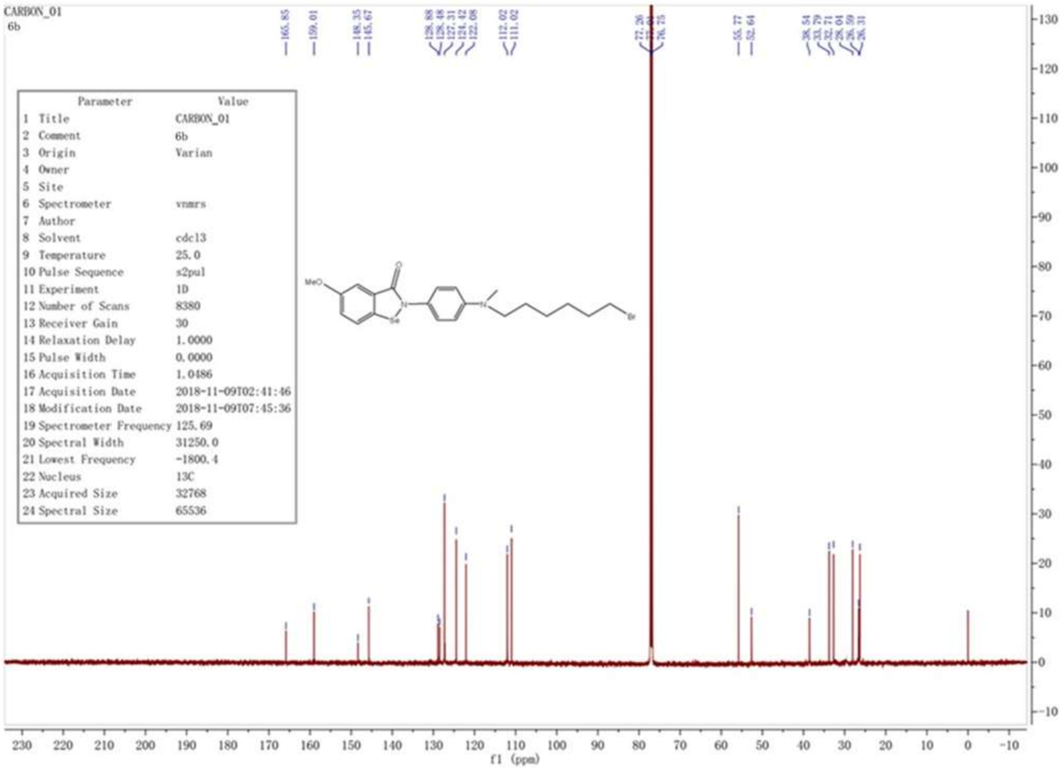

Fig. S26. ^{13}C NMR Spectrum of **6b** in $CDCl_3$ (126 MHz)

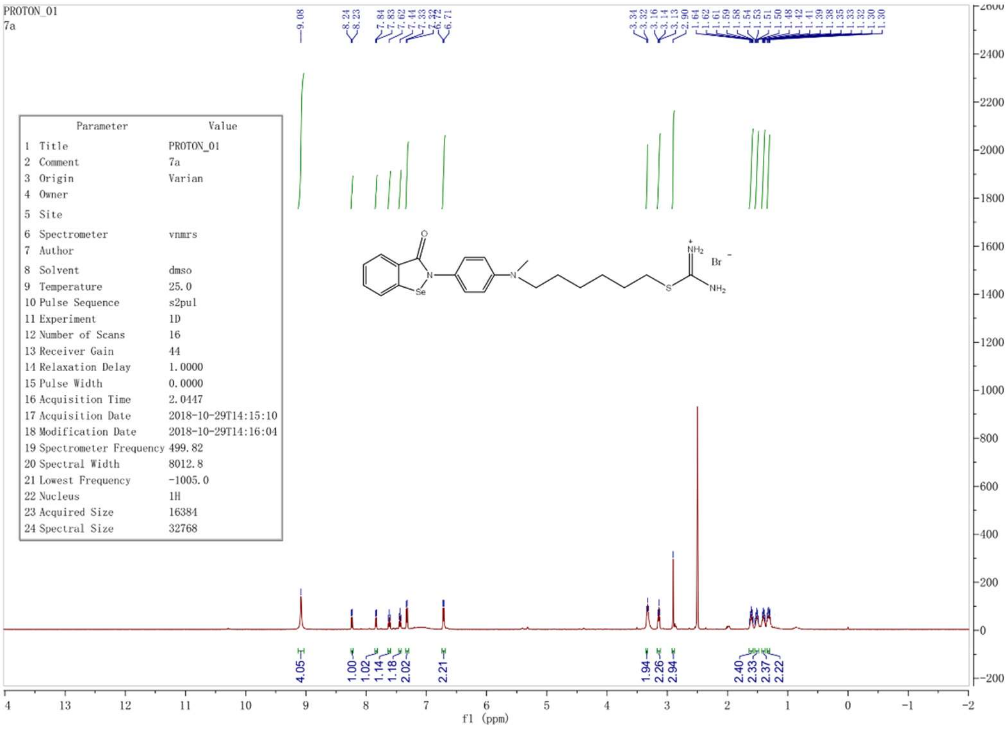

Fig. S27. ^{1}H NMR Spectrum of **7a** in DMSO-d_6 (500 MHz)

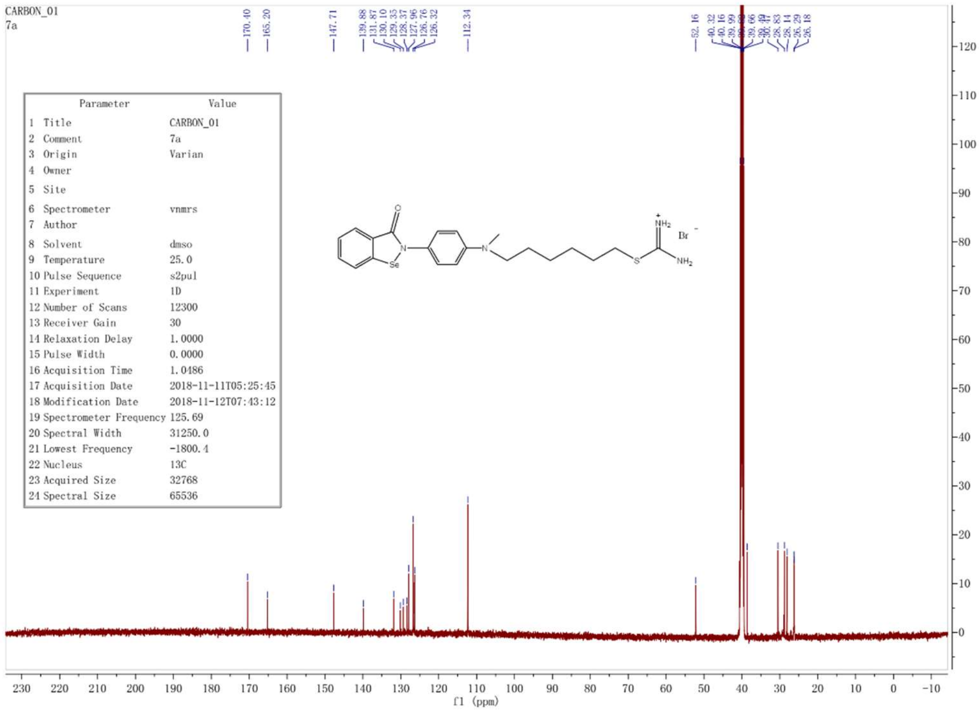

Fig. S28. ^{13}C NMR Spectrum of **7a** in DMSO-d_6 (126 MHz)

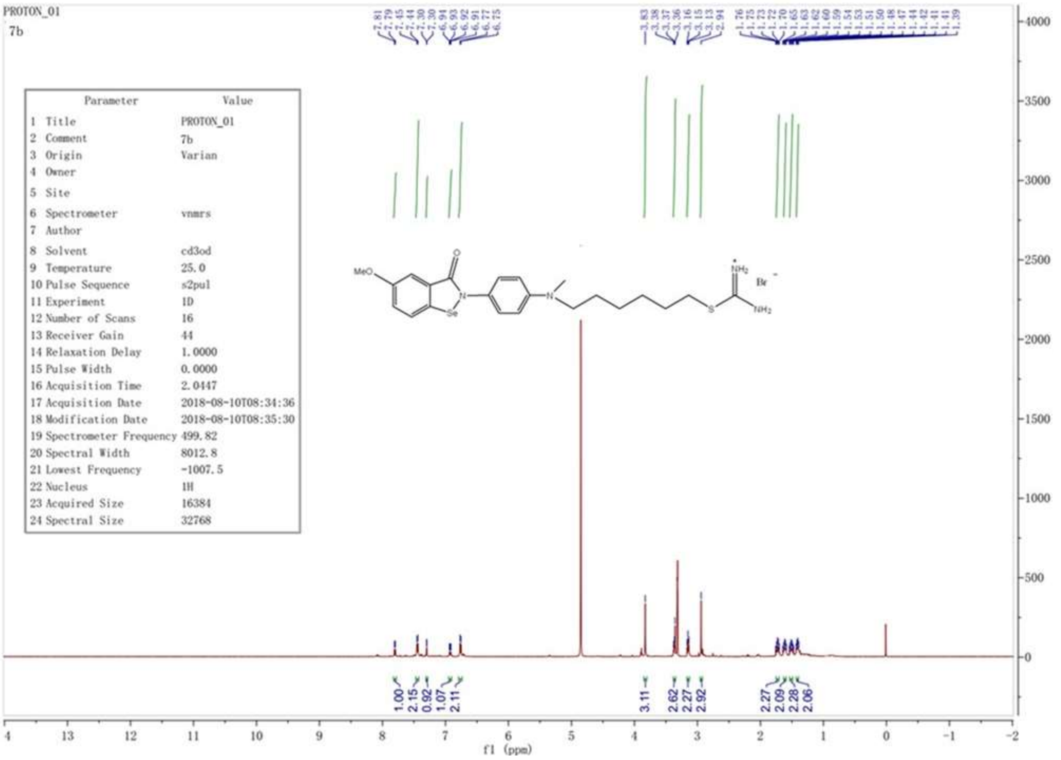

Fig. S29. ^{1}H NMR Spectrum of **7b** in CD_3OD (500 MHz)

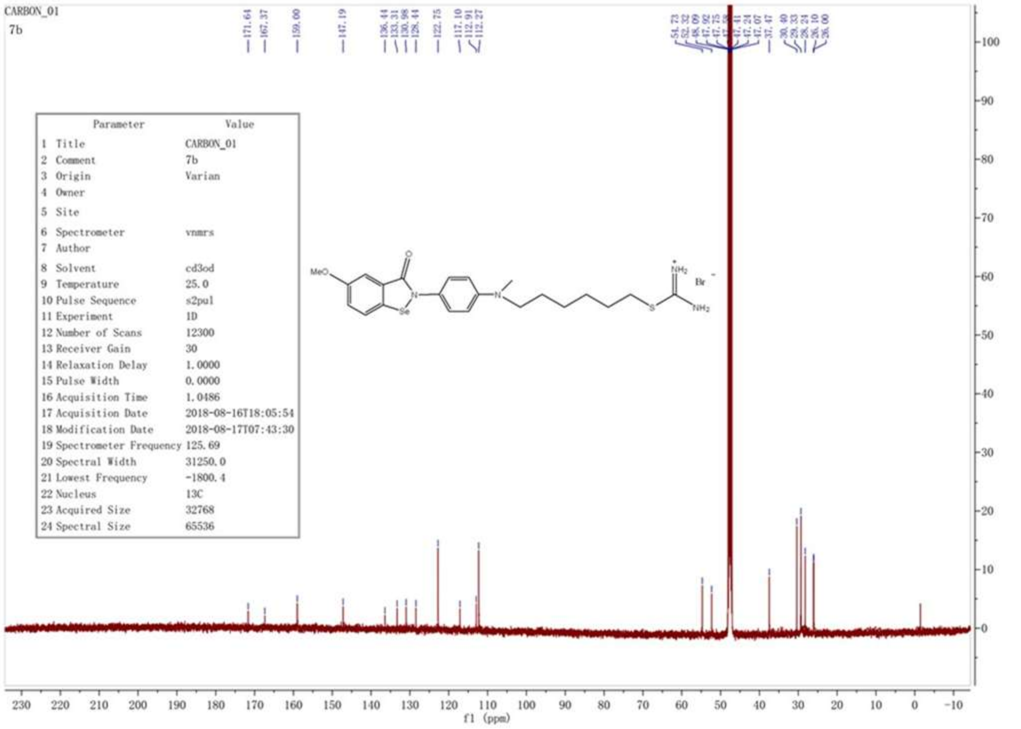

Fig. S30. ^{13}C NMR Spectrum of **7b** in CD_3OD (126 MHz)

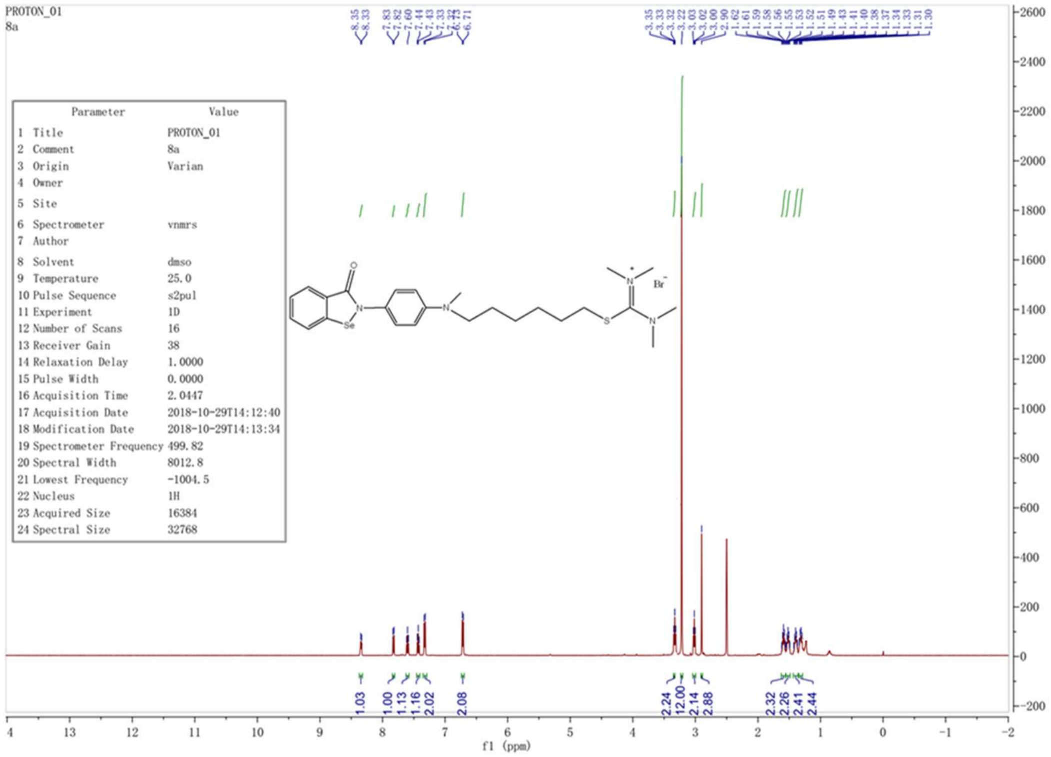

Fig. S31. ^{1}H NMR Spectrum of **8a** in DMSO-d_6 (500 MHz)

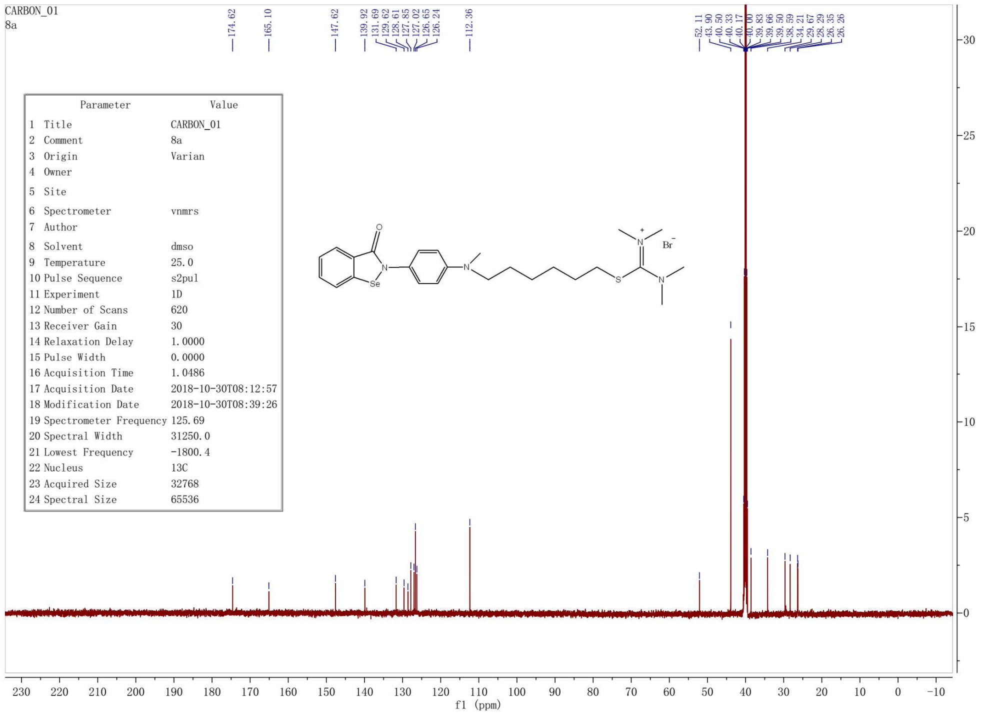

Fig. S32. ^{13}C NMR Spectrum of **8a** in DMSO-d_6 (126 MHz)

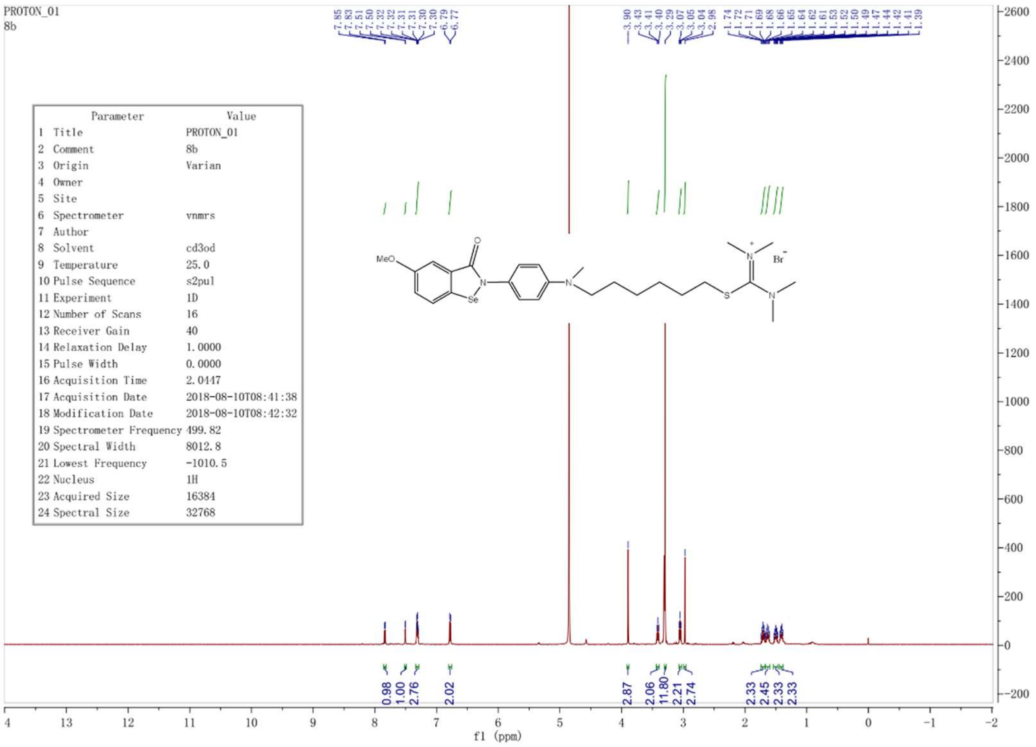

Fig. S33. ^{1}H NMR Spectrum of **8b** in CD_3OD (500 MHz)

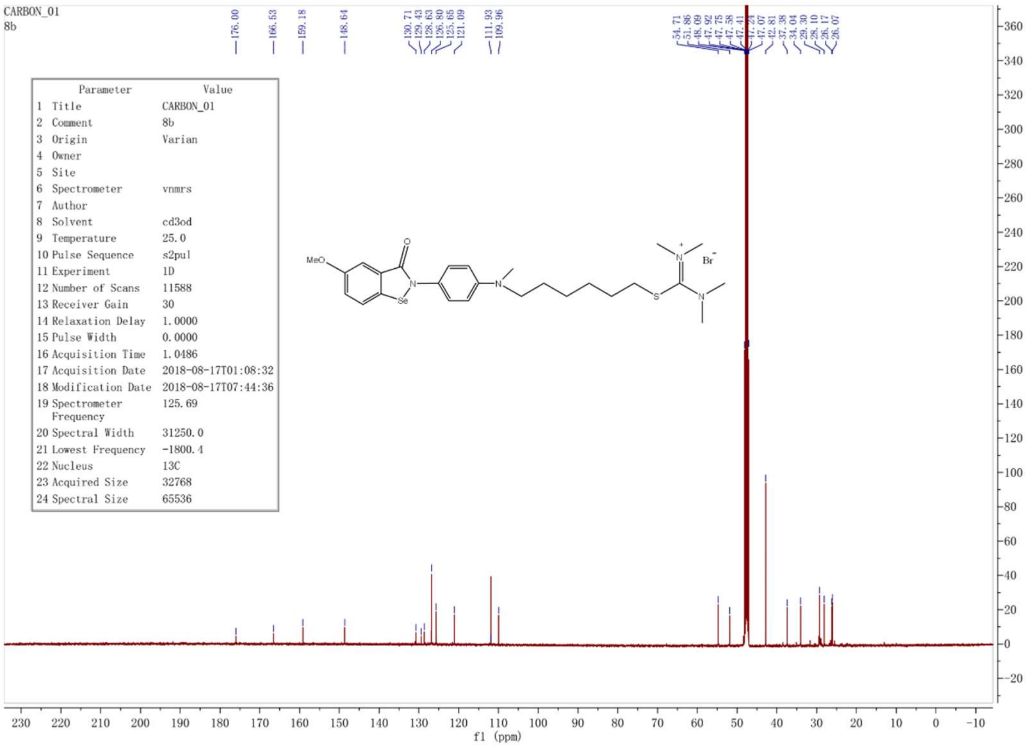

Fig. S34. ^{13}C NMR Spectrum of **8b** in CD_3OD (126 MHz)

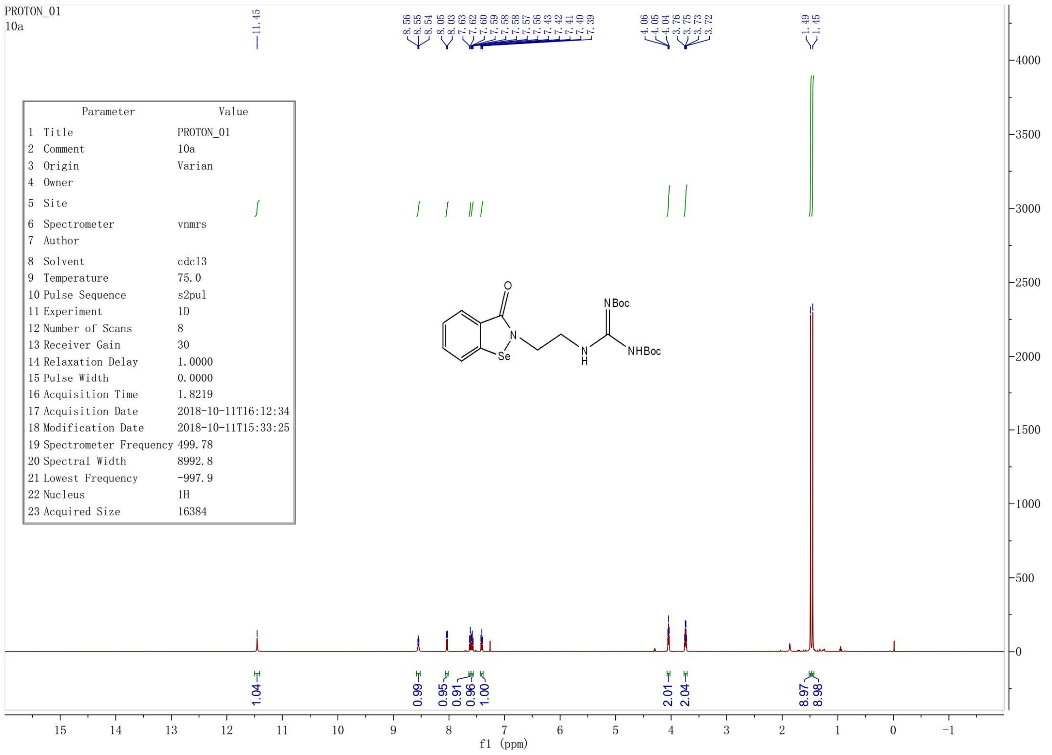

Fig. S35. ^{1}H NMR Spectrum of **10a** in $CDCl_3$ (500 MHz)

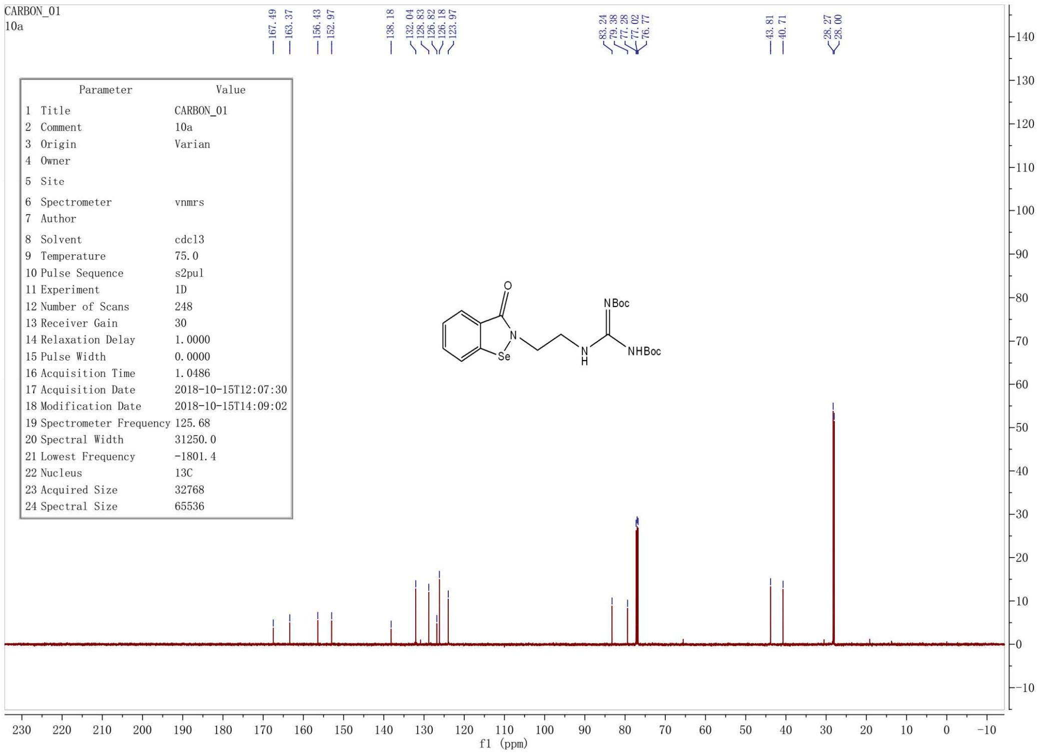

Fig. S36. ^{13}C NMR Spectrum of **10a** in $CDCl_3$ (126 MHz)

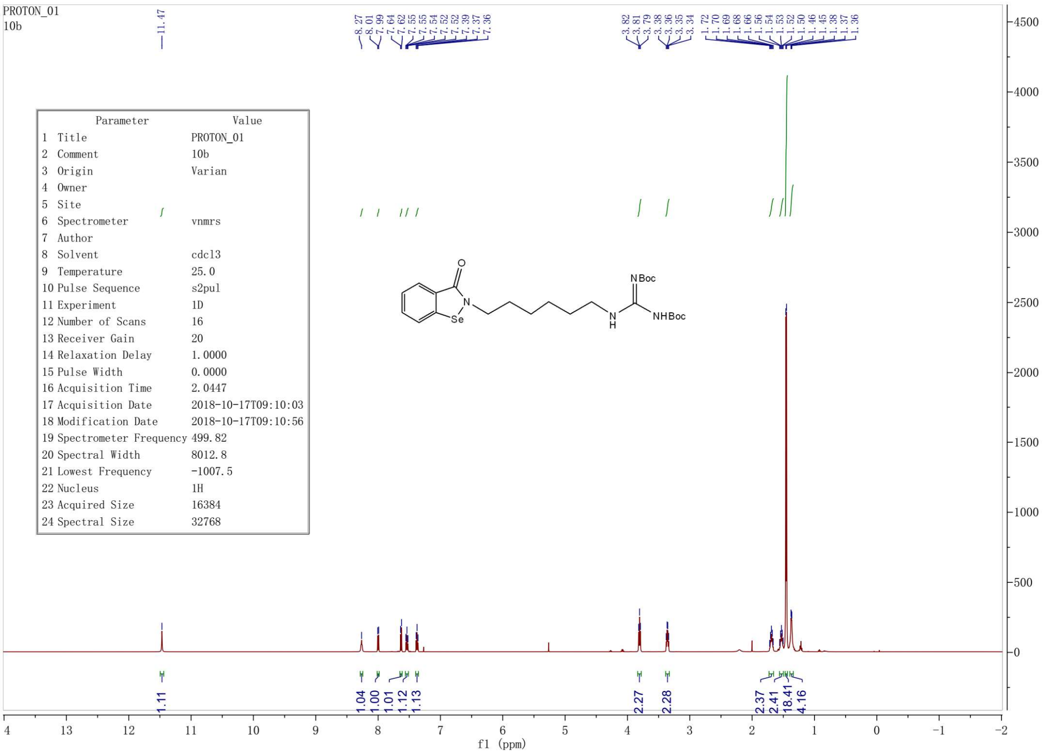

Fig. S37. ^{1}H NMR Spectrum of **10b** in $CDCl_3$ (500 MHz)

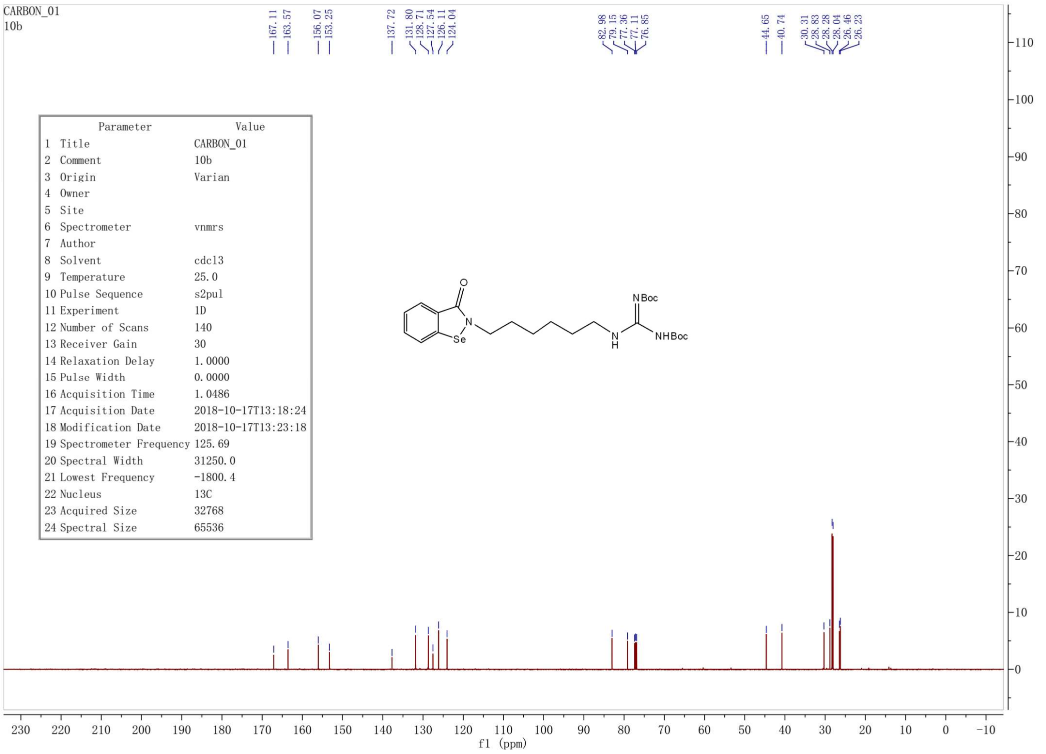

Fig. S38. ^{13}C NMR Spectrum of **10b** in $CDCl_3$ (126 MHz)

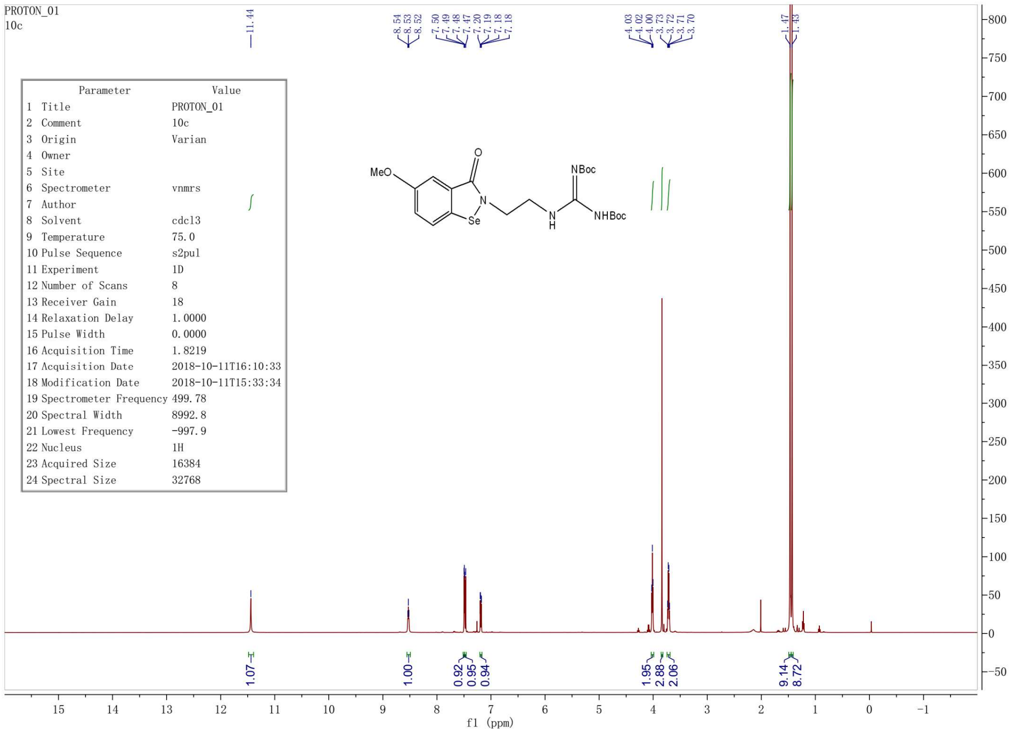

Fig. S39. ^{1}H NMR Spectrum of **10c** in $CDCl_3$ (500 MHz)

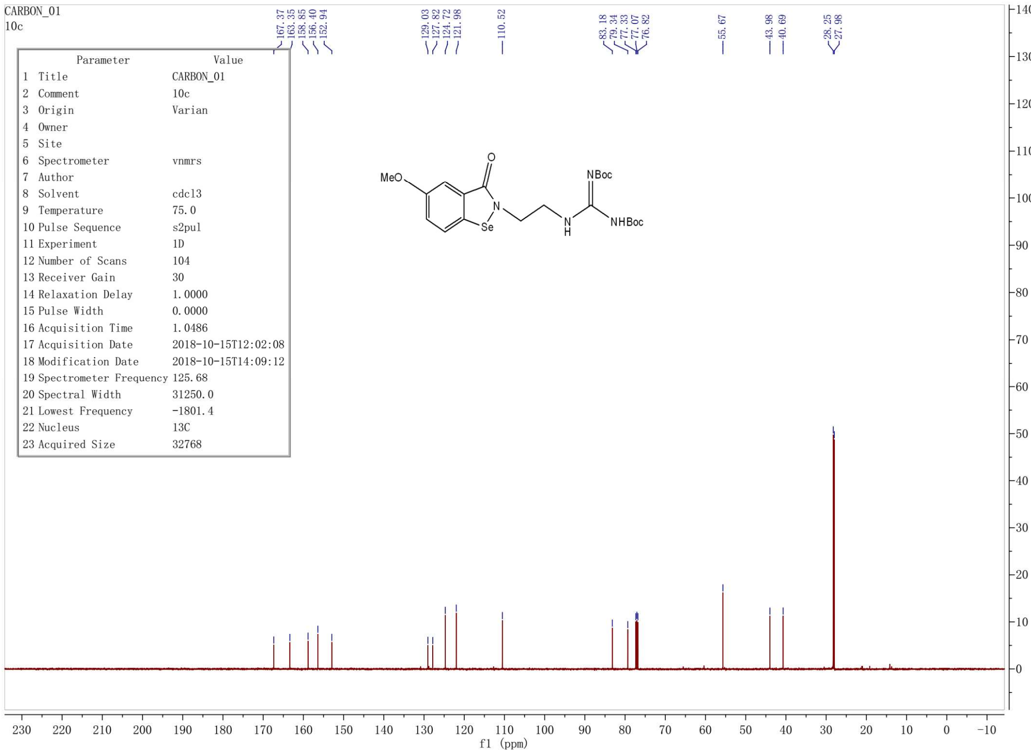

Fig. S40. ^{13}C NMR Spectrum of **10c** in $CDCl_3$ (126 MHz)

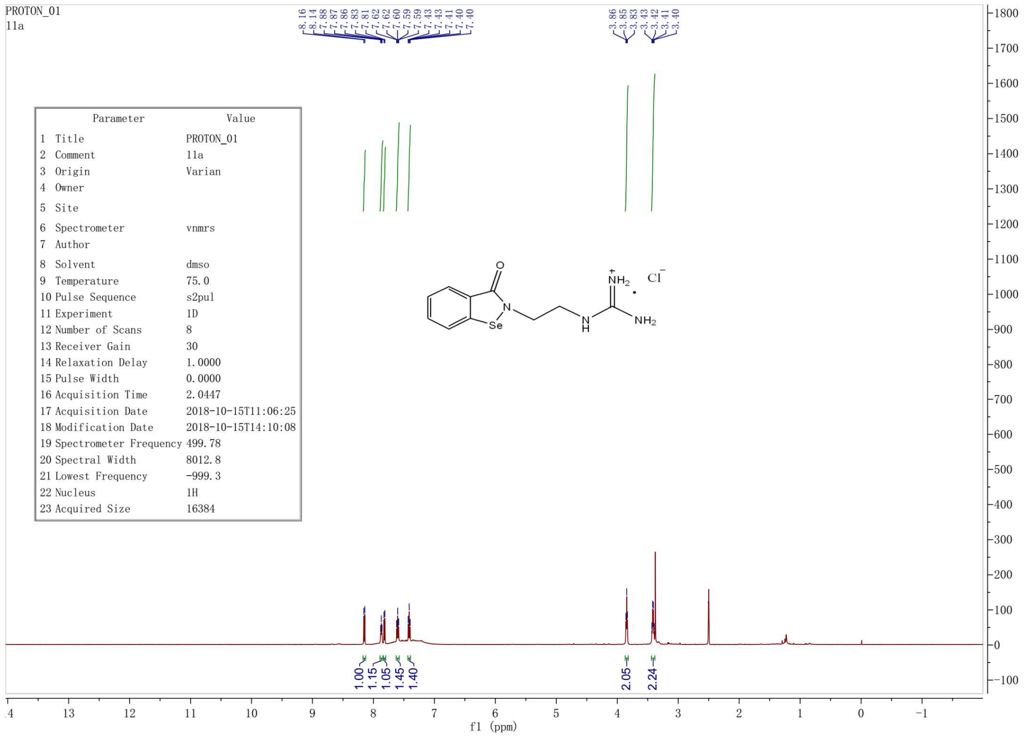

Fig. S41. ^{1}H NMR Spectrum of **11a** in DMSO-d_6 (500 MHz)

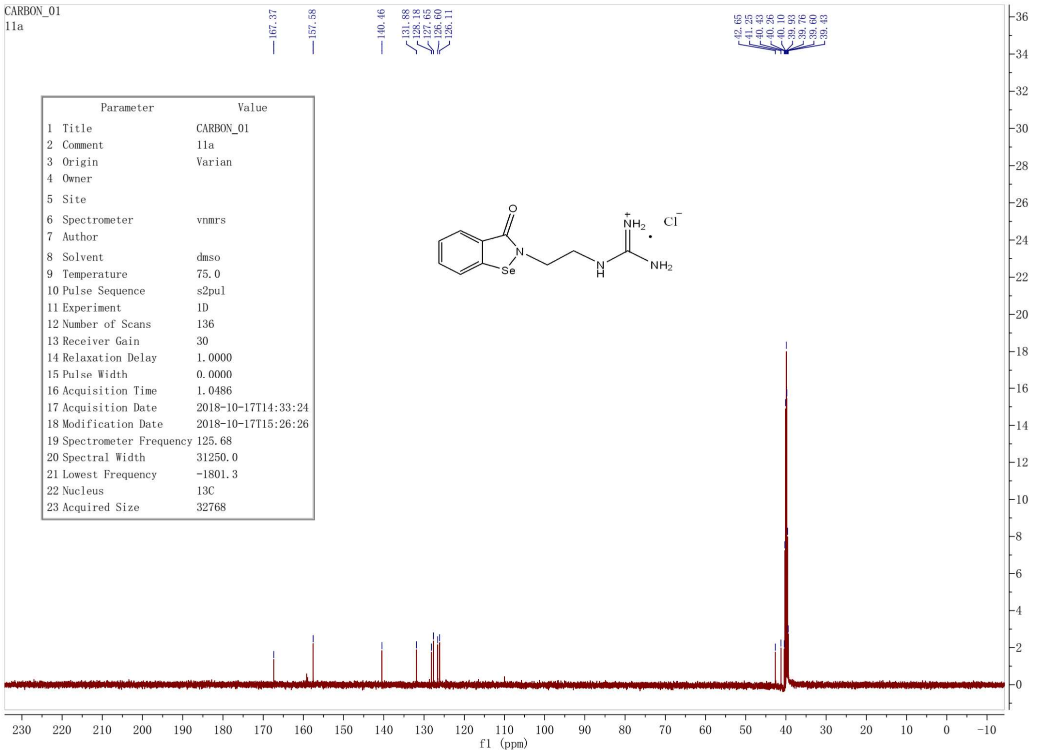

Fig. S42. ^{13}C NMR Spectrum of **11a** in DMSO-d_6 (126 MHz)

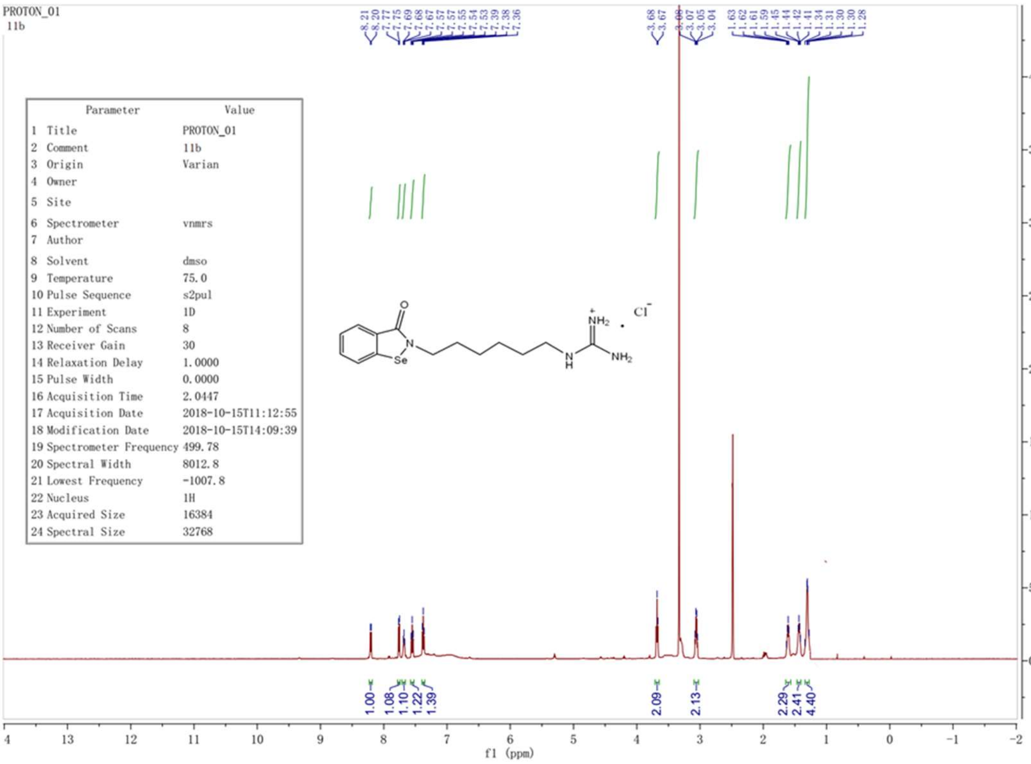

Fig. S43. ^{1}H NMR Spectrum of **11b** in DMSO-d_6 (500 MHz)

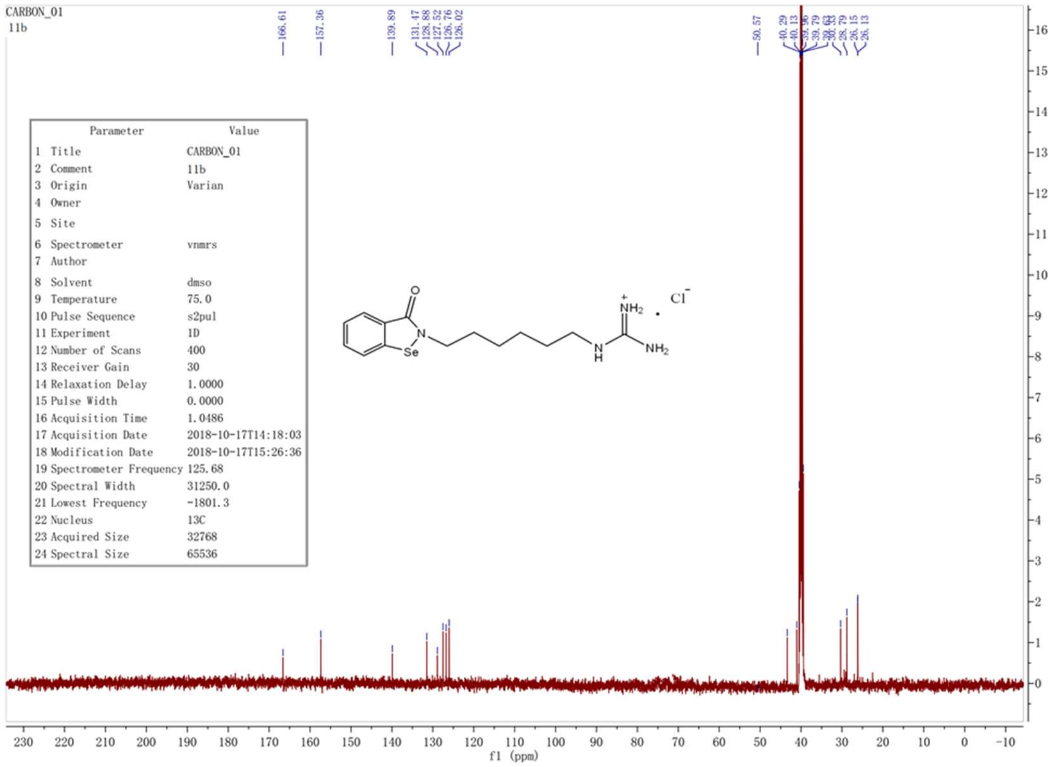

Fig. S44. ^{13}C NMR Spectrum of **11b** in DMSO-d_6 (126 MHz)

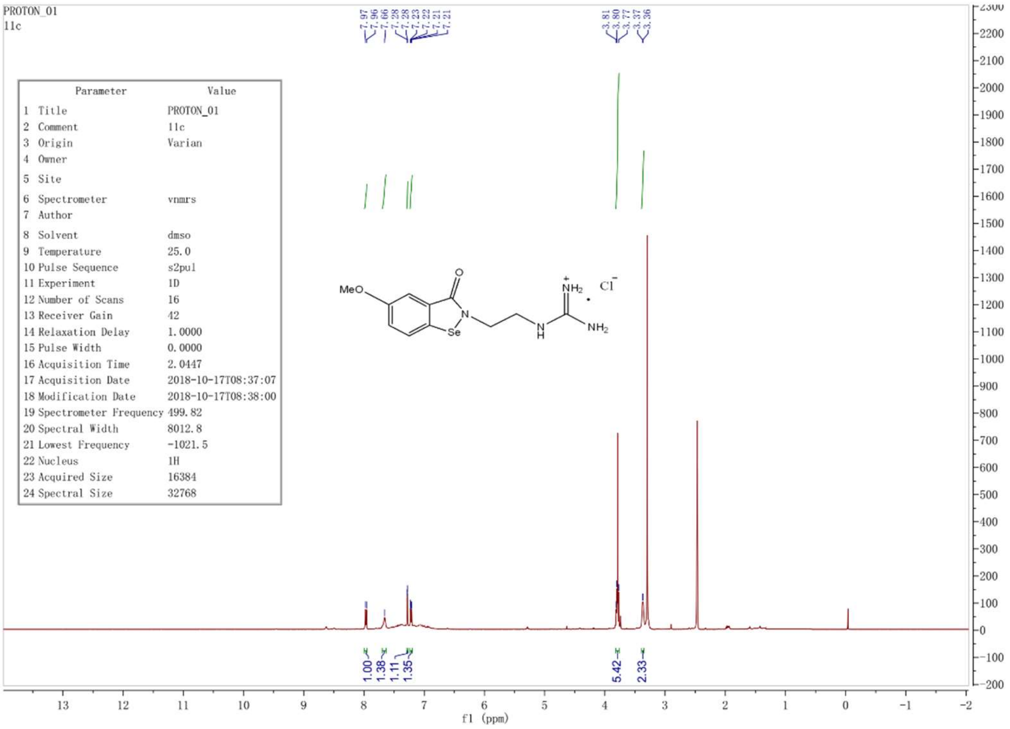

Fig. S45. ^{1}H NMR Spectrum of **11c** in DMSO-d_6 (500 MHz)

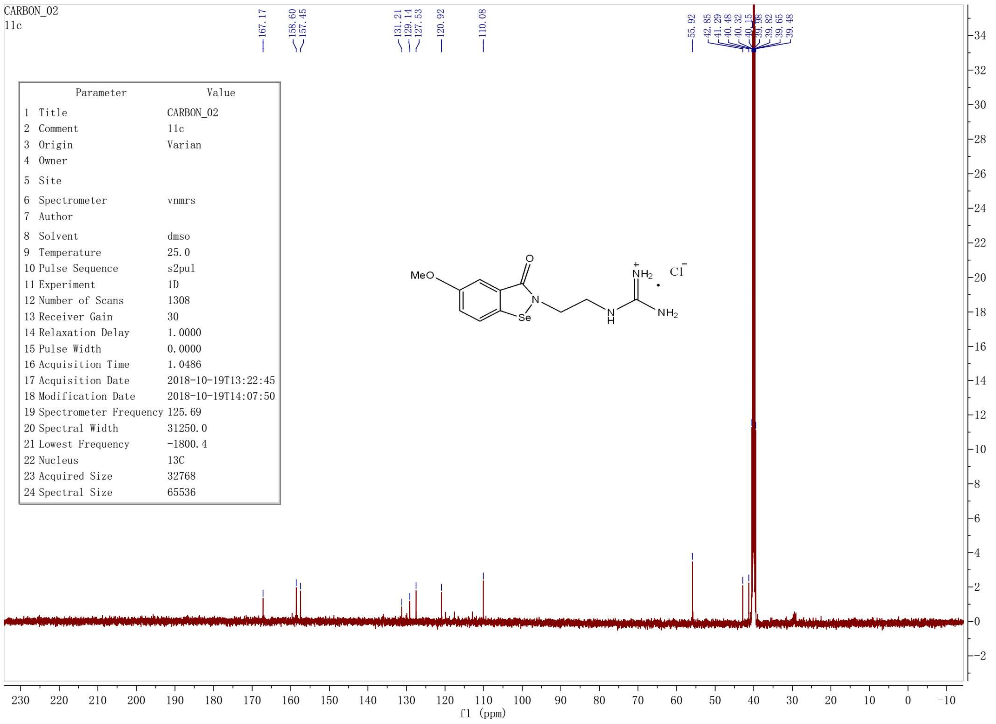

Fig. S46. ^{13}C NMR Spectrum of **11c** in DMSO-d_6 (126 MHz)

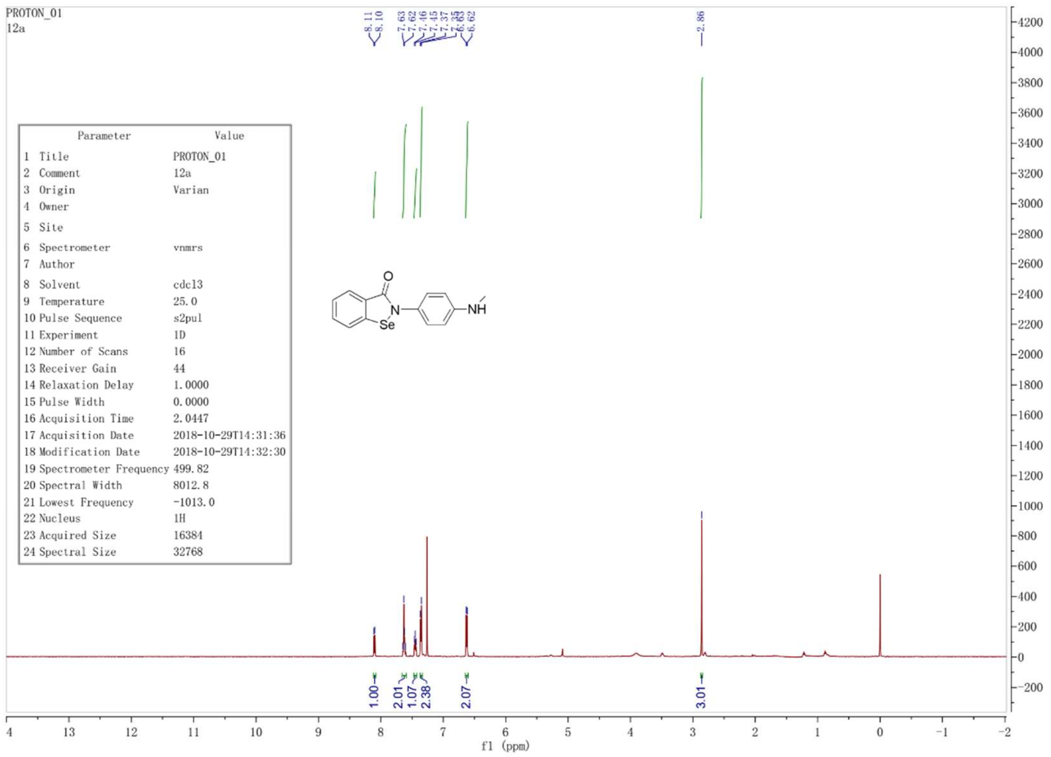

Fig. S47. ^{1}H NMR Spectrum of **12a** in $CDCl_3$ (500 MHz)

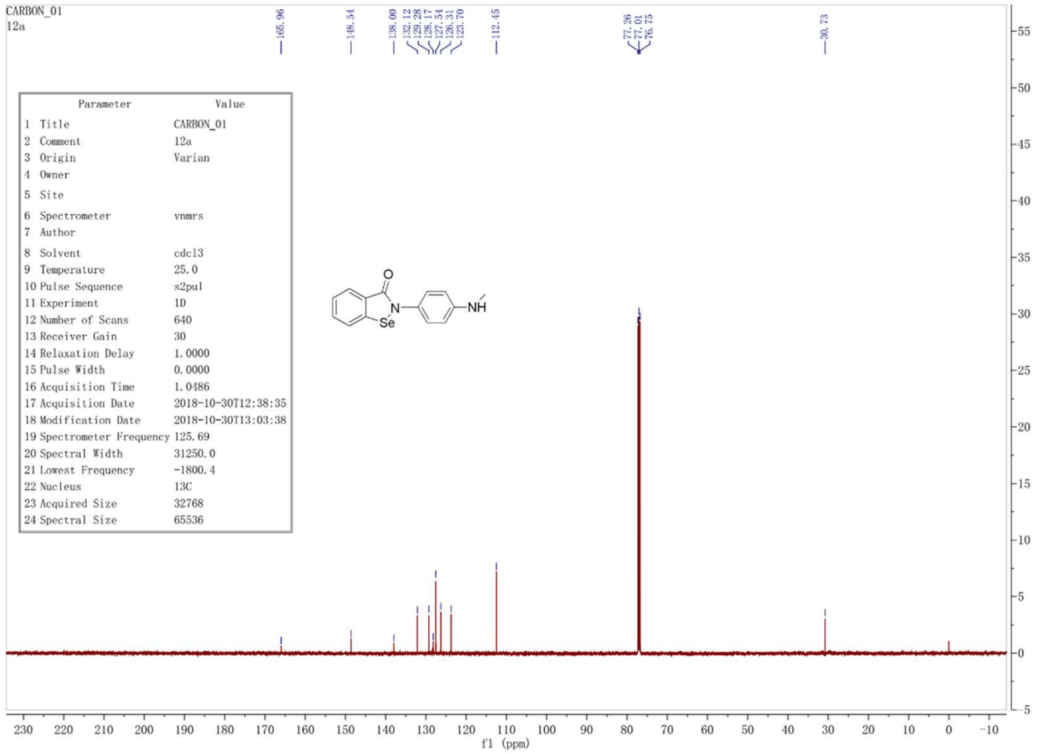

Fig. S48. ^{13}C NMR Spectrum of **12a** in $CDCl_3$ (126 MHz)

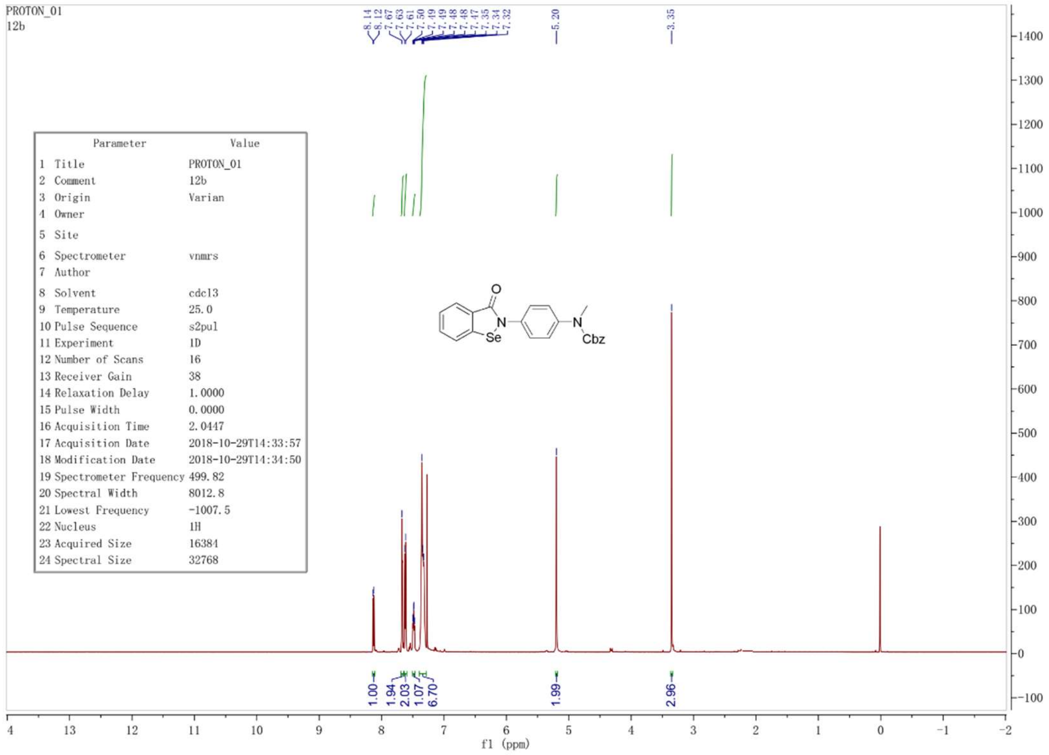

Fig. S49. ^{1}H NMR Spectrum of **12b** in $CDCl_3$ (500 MHz)

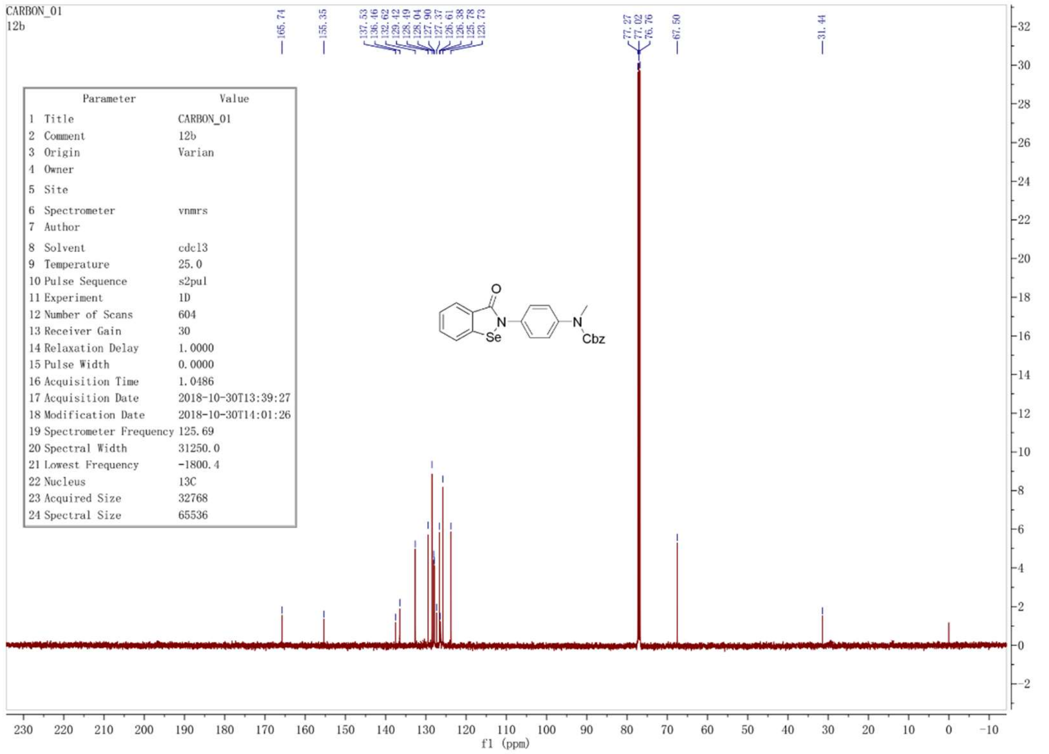

Fig. S50. ^{13}C NMR Spectrum of **12b** in $CDCl_3$ (126 MHz)

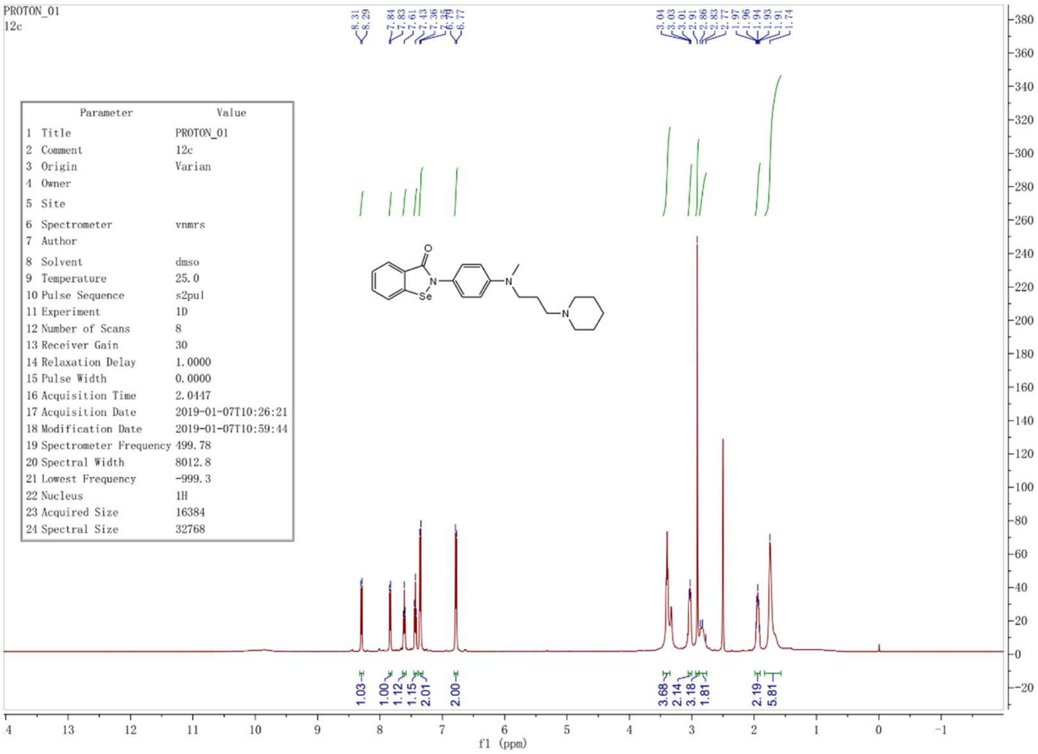

Fig. S51. ^{1}H NMR Spectrum of **12c** in DMSO-d_6 (500 MHz)

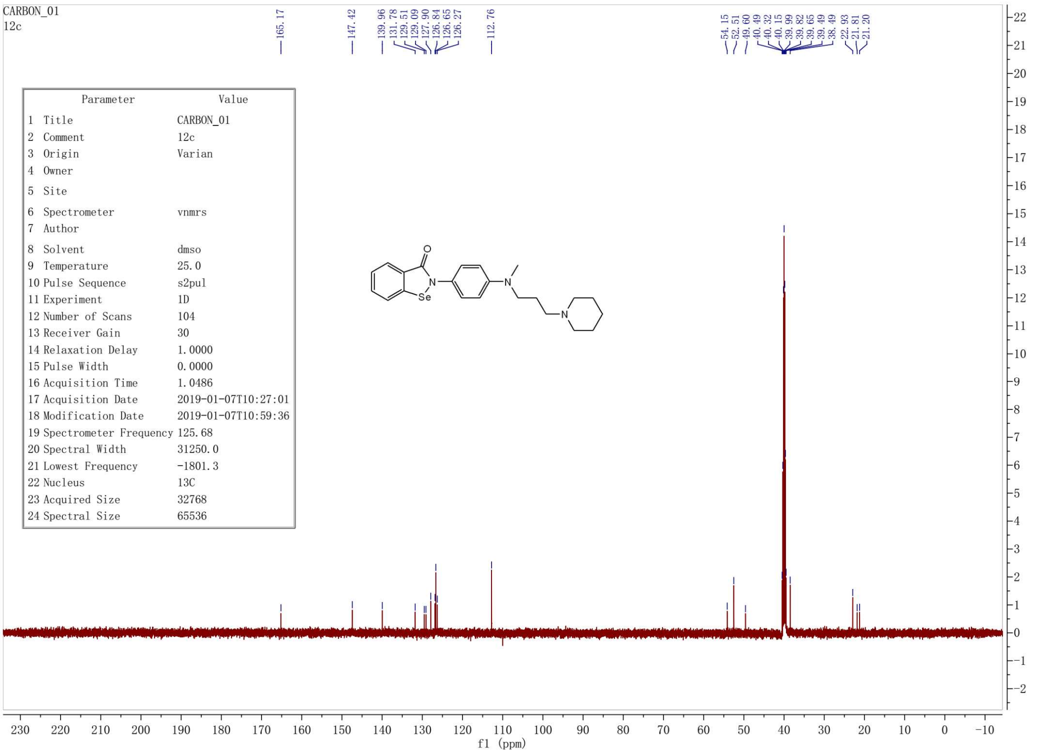

Fig. S52. ^{13}C NMR Spectrum of **12c** in DMSO-d_6 (126 MHz)

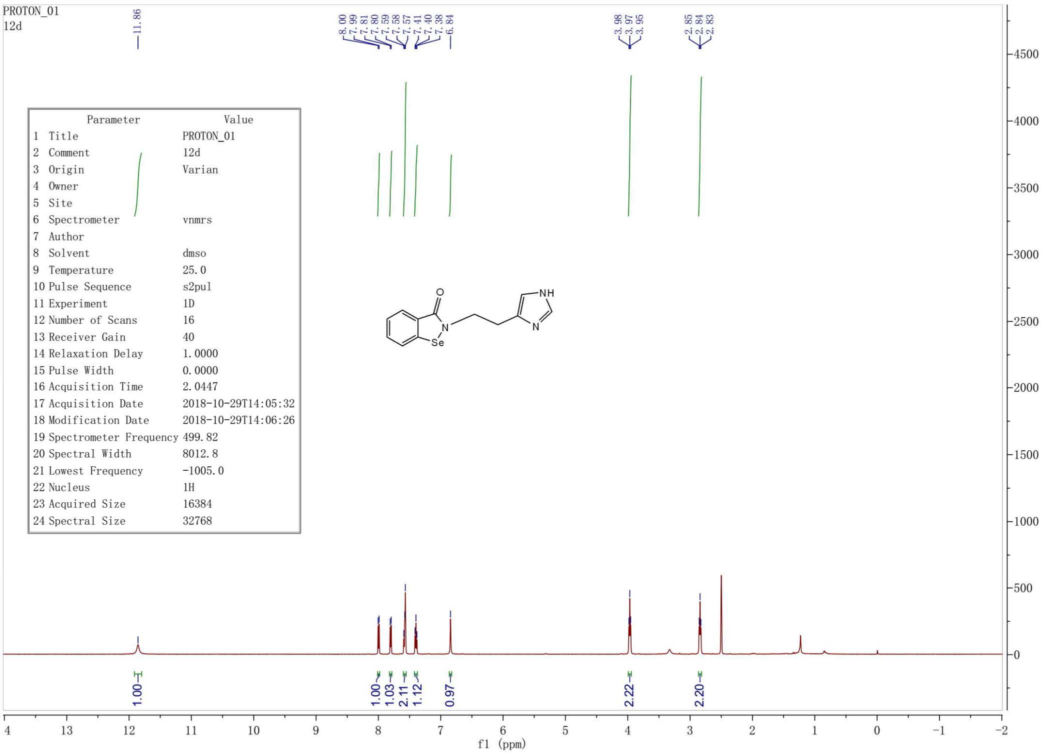

Fig. S53. ^{1}H NMR Spectrum of **12d** in DMSO-d_6 (500 MHz)

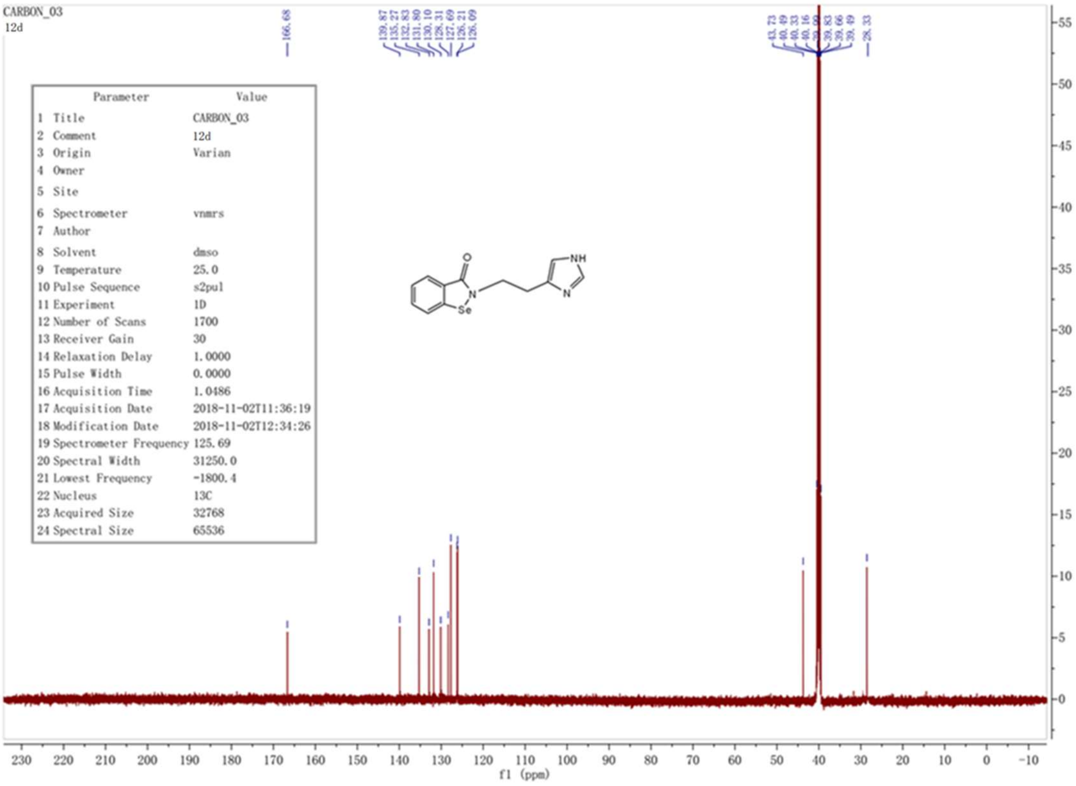

Fig. S54. ^{13}C NMR Spectrum of **12d** in DMSO-d_6 (126 MHz)
